# Combining temporal planning with probabilistic reasoning for autonomous surveillance missions

**DOI:** 10.1007/s10514-015-9534-0

**Published:** 2015-12-28

**Authors:** Sara Bernardini, Maria Fox, Derek Long

**Affiliations:** 1 Department of Computer Science, Royal Holloway University of London, Egham, Surrey TW20 0EX UK; 2 Department of Informatics, King’s College London, London, WC2R 2LS UK

**Keywords:** Automated task planning, Autonomy, UAVs, Quadcopters, Search-and-tracking, Monte Carlo methods

## Abstract

It is particularly challenging to devise techniques for underpinning the behaviour of autonomous vehicles in surveillance missions as these vehicles operate in uncertain and unpredictable environments where they must cope with little stability and tight deadlines in spite of their restricted resources. State-of-the-art techniques typically use probabilistic algorithms that suffer a high computational cost in complex real-world scenarios. To overcome these limitations, we propose a *hybrid* approach that combines the probabilistic reasoning based on the target motion model offered by Monte Carlo simulation with long-term strategic capabilities provided by automated task planning. We demonstrate our approach by focusing on one particular surveillance mission, search-and-tracking, and by using two different vehicles, a fixed-wing UAV deployed in simulation and the “Parrot AR.Drone2.0” quadcopter deployed in a physical environment. Our experimental results show that our unique way of integrating probabilistic and deterministic reasoning pays off when we tackle realistic missions.

**Electronic supplementary material:**

The online version of this article (doi:10.1007/s10514-015-9534-0) contains supplementary material, which is available to authorized users.

## Introduction

Autonomous agents (software entities that operate without direct human intervention and have control over their own actions (Wooldridge and Jennings 1995)) are increasingly used in surveillance applications. Surveillance problems are characterised by two kinds of agents: observers and targets. Observers might be mobile or fixed and targets might be aware that they are being observed, and possibly evasive, or not. In this paper, we focus on search-and-tracking (SaT), which involves searching for a mobile target and tracking it after it is found. We use SaT to demonstrate our approach, but the main features of our technique can be applied to other surveillance operations and data gathering missions (see Sect. 10 for additional details).

At the heart of a number of sophisticated surveillance problems such as SaT, intelligence gathering and hazard identification lies the need to plan complex sequences of behaviours that achieve surveillance goals, which generally consist of gathering as much information as possible given the constraints and communicating findings to human operators. Observers usually operate in unpredictable environments with little stability and rapidly changing information. They must decide what action to perform and how to coordinate with other observers almost instantaneously. They must be highly trained to react quickly, without spending too much time reasoning about alternative courses of action. At the same time observers have limited resources, e.g. fuel or battery, and need to be strategic in deciding what course to follow, looking ahead at their remaining lifespan and fitting their objectives within this time frame. Hence, surveillance missions give rise to many challenges: the management of uncertainty in an unpredictable environment, the handling of restricted resources, the right balance between reactivity and deliberation and the communication of requests and commitments between multiple heterogeneous observers, including human operators in mixed-initiative scenarios.

Although many techniques have been used to address these challenges separately, in our previous work (Bernardini et al. 2013, 2014) concerning surveillance problems with one observer and one target, we show that *automated task planning* is well suited to deal with all these requirements at the same time. Planners offer a route to crafting effective strategies for the observers to achieve their mission goals in the face of all relevant constraints, such as restricted resources, tight deadlines and uncertainty. In Bernardini et al. (2013), we introduce a plan-based approach to SaT by expressing the search phase of a SaT mission as a *deterministic* task planning problem. We use an automated temporal planning tool to solve this problem and generate robust strategies for the observer. Given the deterministic formulation of the problem, our technique leads to good policies when the target’s behaviour is predictable, but incurs inaccuracies when the target acts in a more sophisticated way, as we neglect important probabilistic information about the physical motion of the target in the environment.

In this paper, we combine our previous plan-based approach to SaT with *Monte Carlo* (MC) methods. We take our approach one step further by integrating it with probabilistic reasoning based on the target’s motion model and on the environment’s structure. In our novel *hybrid* technique, we apply MC simulation (MCS) to estimate the probable trajectories of the target and construct a fine-grained Probability Distribution (PD) map for the target location, while using planning to reason about this map and create long-term strategic plans for the observer that maximise the probability of rediscovering the target. The MCS works on the basis of historical information about the target, its physical model of movement and topological information about the area of operations. The experimental results presented in Sect. 8 show that our hybrid method outperforms other existing techniques as well as our previous approach.

The use of automated task planning for SaT missions has received little attention so far, while probabilistic approaches based on Recursive Bayesian Estimation (RBE) have been explored in more depth. Efficient solutions to SaT have been proposed under restrictive simplifying assumptions such as the search area being small (one/two square km), the temporal horizon being short (a few minutes) and the target’s motion model being simple (e.g., targets being stationary or in Markovian motion) (Stone 1975; Bourgault et al. 2006; Furukawa et al. 2006; Lavis and Furukawa 2008; Lin and Goodrich 2014). Although this purely probabilistic approach is successful for small-scale and simple SaT problems, it fails in the face of all the constraints that characterise real-world SaT operations because it becomes computationally too expensive.

In our work, we remove the typical assumptions behind state-of-the-art approaches to SaT and consider scenarios with the following features, which characterise the majority of realistic missions: (i) the target moves according to its own intentions; (ii) the target moves across a large geographical area; and (iii) the target needs to be tracked over a long period of time. Our method is flexible enough to deal with smaller areas and shorter temporal horizons too, when needed. In addition, unlike many SaT approaches that focus either on target following or on target searching, our hybrid approach deals with the entire *decision-making process* behind a SaT mission in a dynamic fashion. Alongside constructing efficient strategies for the observer to keep track of the target during both tracking and searching, we plan other types of actions for the observer that depend on the specific circumstances in which the target was lost and on the resources available to the observer at that time. For example, we can plan actions to renew depleted resources and to manage noisy sensors. Thanks to generative task planning, we craft *long-term* programmes of activity for the observer that address the constraints of the environment and the limits of the searching vehicle itself. At the same time, the plans generated are much more accurate and robust, since the planning process is now informed by probabilistic information based on a physical model of the target motion.

In this paper, not only do we describe our new approach to SaT (Sects. 2–4), we also give a full account of the *knowledge engineering process* by which we combine probabilistic reasoning with deterministic planning (Sects. 5, 6) and formulate autonomous search as a planning task (Sect. 7). For the actual planning phase, we use an off-the-shelf planner, Optic (Benton et al. 2012), that is capable of dealing with all the advanced modelling features of our domains. In so doing, we prove that generic domain-independent planning technology is mature enough to handle real-world complex missions that have not traditionally been seen as task planning problems.

## Search-and-tracking

SaT is the problem of searching for a mobile target and tracking it once it is found. Examples of SaT operations are a UAV searching for life-rafts drifting with current, a police helicopter tracking a suspected criminal over a road network and a small drone escorting a worker who performs risky tasks in a factory.

**Fig. 1:**
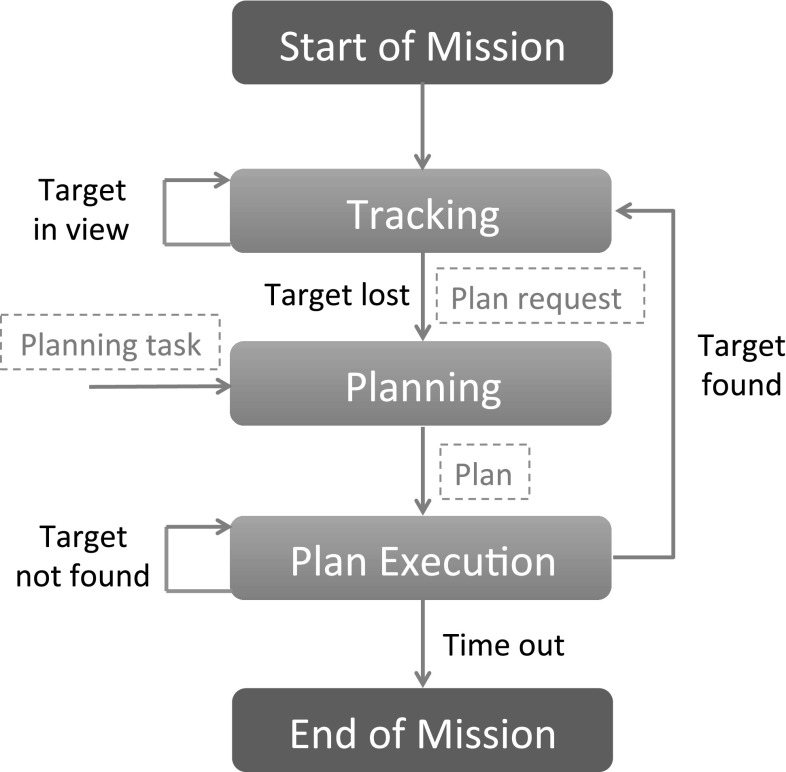
Structure of a typical SaT mission

A SaT mission aims to follow the target to its destination and proceeds in two phases, which constantly interleave (see Fig. 1): (i) *Tracking*: the observer flies over the target, observing its progress; and (ii) *Search*: the observer loses the target and flies a series of manoeuvres to rediscover it. Once the target is spotted, the observer switches back to tracking.

We are interested in land SaT operations with a single observer and a single target. The target is an object that moves according to its own intentions and proceeds at a slower pace than the observer. We assume that the target needs to reach a specific destination and chooses an efficient path to do so, without trying to evade the observer. This is a plausible assumption as the target might be cooperating with the observer or simply be unaware of its presence, but we recognise that the extension to consider evasive actions on the target’s part is important future work. The observer knows the map of the search area and is equipped with imaging systems to scan it and observe the target. Observation is susceptible to error and interference from the features in the environment.

The choice of a specific SaT hardware platform depends on the characteristics of the SaT mission. Fixed-wing UAVs are usually employed when the mission takes place in large and heterogeneous geographical areas and over long temporal horizons because UAVs are fast and have good endurance. On the other hand, Micro Aerial Vehicles (MAVs) (e.g., quadcopters) are used in scenarios that are inaccessible to large unmanned vehicles, such as cluttered outdoor settings, indoors and in proximity to people. SaT operations with MAVs are typically short and take place in small areas since they have a restricted payload, i.e. reduced computational power, noisy sensors and a short battery life.

We demonstrate our approach to SaT by using two different platforms, a fixed-wing airplane and a low-cost quadcopter. In both cases, we are interested in missions that stretch over long temporal horizons and wide areas, relative to the scale of the vehicle used. Our approach is the same for both platforms, but the planning instances that we build to represent the problem for the two scenarios are different and account for the specific physical features of these platforms.

## Plan-based approach to SaT

**Fig. 2:**
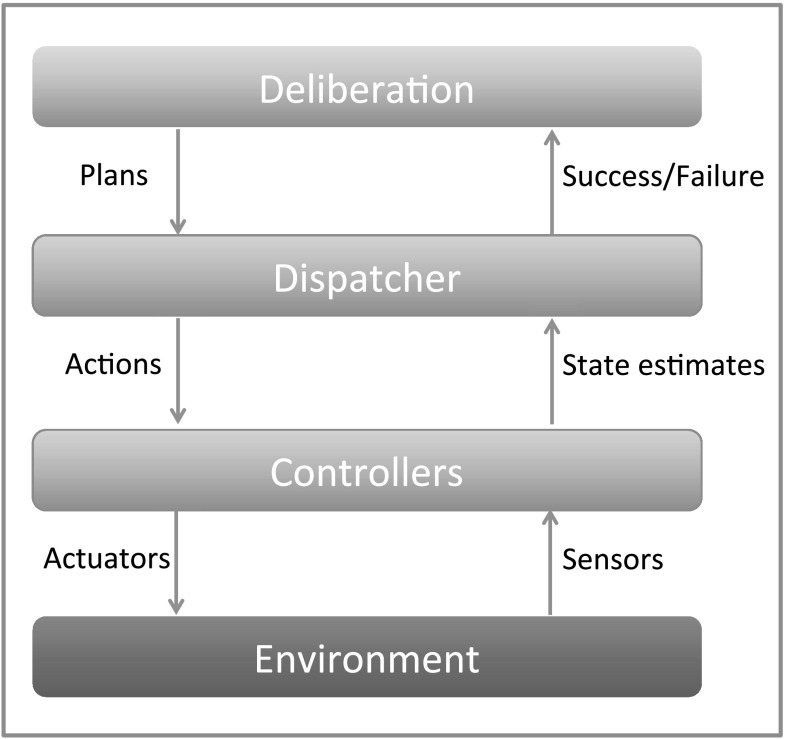
Layered architecture for autonomous SaT missions

During the tracking phase of a SaT mission, the observer follows the target, observing its progress. At first sight, tracking might appear to be a planning problem, in particular a temporally extended goal problem where the goal is to keep the target in view as long as possible. However, as we consider tracking in more depth, we see that it is in fact a reactive control problem, since the target’s intentions are unknown and the observer can only respond to the target’s movements moment by moment. It is when the target is out of view that we need to carefully plan a recovery strategy to relocate it. Our approach is therefore to track the target reactively while it is visible to the observer and to plan a recovery strategy every time it is lost by using an automated planning tool. If the observer rediscovers the target while executing the plan, the observer abandons the plan and switches back to tracking. Figure 2 shows the layered architecture that we use to support autonomous SaT missions. In this paper, we focus on the deliberation layer only. An account of how we implement the other layers can be found in Cashmore et al. (2015).

We manage the tracking phase through a *reactive controller* equipped with sensing capabilities. If the speed of the observer and the target are comparable, the observer simply flies over the target. However, if the observer flies much faster than the target and cannot hover, the flight path of the observer is a circle of fixed radius centred on the target. The radius depends on the observer’s capabilities: it cannot be greater than the imaging equipment’s range, nor can it be shorter than the observer’s turning radius at current speed. We assume that the observer flies in a mid-range circle between these extremes. As the target moves, the circle moves with it, so the observer’s flight path describes a prolate cycloid over the ground.

When the observer fails to follow the target, it must attempt to rediscover it. For a short period after losing the target, the observer simply tracks its predicted location, since the target cannot move fast enough to significantly deviate from this prediction. However, after a longer period, it is necessary to make a more systematic effort to rediscover the target by directing the search into specific places. This is when planning comes into play. We formulate the search phase as a *planning task* consisting of deciding where exactly to search for the target and what manoeuvres to use. The goal is to maximise the likelihood of finding the target while favouring manoeuvres that minimise the use of the observer’s consumable resources and, in the case of the quadcopter, generate robust sensor measurements for stable flight. We use *MC simulation* to aid the planning process in making decisions by suggesting probable trajectories that the target might follow during its course of action.

## Search operations

In line with SaT and SaR (Search-and-Rescue) international standard guidelines (IMO 2013; NATSAR 2011; CSAR 2000), we employ the following procedure to manage the search phase of a SaT mission, where each step is fully automated in our system:

1. Determine the optimal area where the search effort should be deployed, which is an area where the target is most likely to be found;
2. Divide this area into appropriate sub-areas for assignment to individual *search patterns*, which are sets of manoeuvres for surveying specified regions;
3. Generate a set of search patterns to optimally cover each sub-area and choose their orientations;
4. Select a subset of the generated search patterns for execution and sequence them over time; and
5. Execute the chosen sequence of patterns, switching back to tracking if the target is rediscovered.

**Fig. 3:**
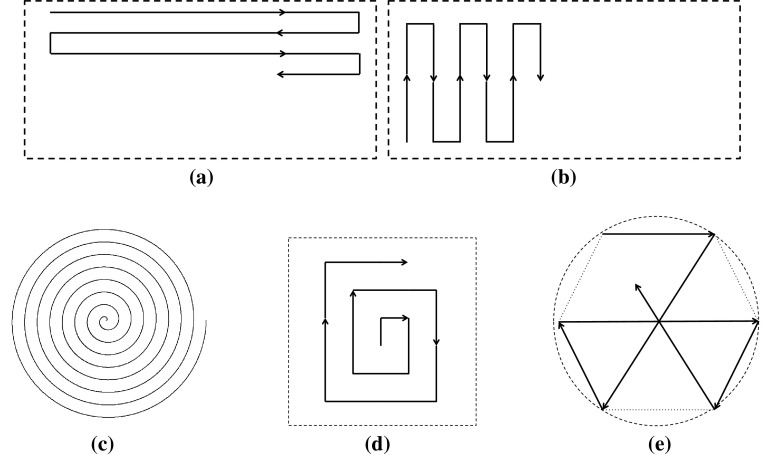
Standard search patterns used in SaT missions. **a** Parallel Track Search. **b** Creeping Line Search. **c** Spiral Search. **d** Expanding Square. **e** Sector Search

Steps 1 and 2 depend on information regarding the specific mission. In real-world SaT operations, these steps are performed based on many biasing factors: the target last known position (LKP), its intentions if predictable, its size and motion characteristics, possible hazards, results of previous searches, the terrain characteristics, the road network’s structure and the weather conditions. These features are used to make predictions on where the target might be over time and to construct a PD for the target location. In short, the outcome of Steps 1 and 2 consists of:

- a confined search area, usually a circular sector that is centred on the LKP of the target;
- a PD for the target position defined across this sector and constructed considering the above-mentioned factors; and
- a number of points within the sector that present the highest probability of rediscovering the target and on which candidate search patterns are deployed.

To calculate these three pieces of information, we perform MCS based on the target motion model. In our previous work (Bernardini et al. 2013), we construct the target PD manually by considering the features of the road network and the terrain in the search area. This calculation is very efficient, but our experiments show that the plan-based approach suffers from the use of this coarse-grained distribution. Through MCS, on the other hand, we construct a much more precise PD map for the target location because the simulation is based on the target physical model of motion and incorporates information that reflects the environment structure as well as the target’s intentions and physical characteristics (e.g. its minimum and maximum speed on roads). Our MC-based approach is very general as any new assumption regarding the target motion can be easily integrated in the simulation with the resulting PD expressing such additional information.

Building on the outcome of the previous steps, Step 3 is concerned with generating specific patterns to cover the points with the highest probability of rediscovering the target within the optimal area of operations. Again, we adhere to international guidelines (IMO 2013; NATSAR 2011; CSAR 2000) and use the following standard search patterns (see Fig. 3 for a pictorial representation of these patterns except the last one):

- *Lawnmower Search* (LS), which consists in flying along straight lines with 180$^\circ$ turns at the end. Based on the sweep direction, there are two types of lawnmowers:
  - *Parallel Track Search* (PTS), if the search area is large and level, only the approximate location of the target is known and uniform coverage is desired;
  - *Creeping Line Search* (CLS), if the search area is narrow and long and the probable location of the target is thought to be on either side of the search track;
- *Spiral Search* (SS) and *Expanding Square Search* (ESS), if the search area is small and the position of the target is known within close limits;
- *Sector Search* (SES), used similarly to the ESS, it offers several advantages: concentrated coverage near the centre of the search area, easier to fly than the ESS, and view of the search area from many angles;
- *Contour Search* (CS), used to patrol obstacles, always assumed to be polygonal in our application.

In Step 4, a subset of the candidate search patterns generated in Step 3 needs to be selected and sequenced for execution. While these tasks are usually managed manually in real missions, with the consequence of being a time-consuming and error-prone process, we formulate the problem of selecting and sequencing search patterns as a *planning problem* and use a high-performing planner to solve it. In Step 5, the plan devised in Step 4 is executed by the observer until completion. Only if the target is rediscovered during execution, the plan is abandoned and tracking is resumed. If the target is lost again during the same mission, then Steps 1–5 are performed again from scratch.

In summary, in order to implement Steps 1–5, we need to handle three main tasks:

(i) Produce a fine-grained probability map for the optimal area of operation;
(ii) Generate a set of candidate search patterns to optimally cover the area of operation, which involves deciding their position, size, orientation and type; and
(iii) Select a subset of search patterns and sequence them for execution.

The first task is managed via MC methods and is explained in Sect. 5. The second task is performed based on the PD map for the target’s location and is described in Sect. 6. The final task is performed by applying automated planning and is detailed in Sect. 7.

**Fig. 4:**
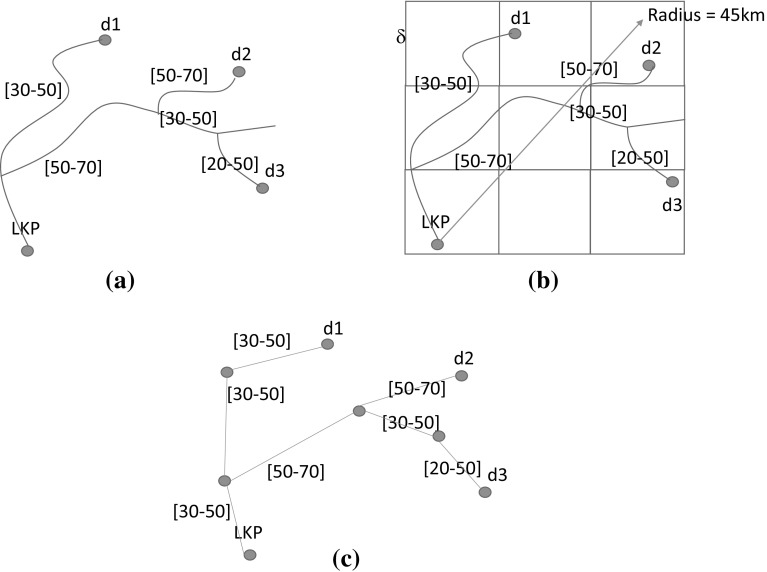
Building a graph $\mathcal {G}=\langle \mathrm {V},\mathrm {E}\rangle$ based on the road network. **a** Representing a very small RN with the target LKP and three major destinations: $d_1, d_2$ and $d_3$. Roads are labelled with the minimum and maximum speed allowed. **b** Gridding the chosen search area and the underlying RN, with the side of each square cell being $\delta$. **c** Resulting graph representation at $\delta$-granularity: $\mathcal {G}=\langle \mathrm {V},\mathrm {E}\rangle$, where $\mathrm {V}$ is the set of the cells in the grid and $(v,w) \in \mathrm {E}$ if there is a road in the RN that connects the cells *v* and *w*

## Trajectory prediction via probabilistic modelling

In SaT, the planner’s role is to select a set of search patterns and sequence them over time. To operate effectively, we need to provide the planner with an initial pool of *candidate search patterns* from which to choose those to execute. It is preferable to keep the cardinality of this set small so as to reduce the computational complexity. We perform MCS to identify points in the search area that present the highest probability of finding the target at different points in time and then create candidate patterns that have those points as their centres.

### Graph construction

We assume that the target is located in Euclidean 2-space and that this space is characterised by a *road network* (RN), where each road is a sequence of connected line segments. Roads can be of different types, where the type establishes the speed limits on the road. The target motion on each segment is assumed to follow a constant speed randomly and uniformly sampled in a interval $[\nu ^\mathrm{min}, \nu ^\mathrm{max}]$, where $\nu ^\mathrm{min}$ and $\nu ^\mathrm{max}$ are the minimum and maximum speed allowed in that segment depending on the road type.

We take a circular sector centred on the target’s LKP as the optimal search area. This sector extends outwards with its symmetry axis aligned with the target’s average bearing over the period it was observed. The radius of the sector is determined by considering both the target’s travel speed and the time period over which the search is planned to be performed (see Sect. 8 for the formula that we use in our simulation). We then superimpose a grid on this sector, with the side of each square cell being $\delta$. To represent the topology of the search area, we build a graph $\mathcal {G}=\langle \mathrm {V},\mathrm {E}\rangle$ based on the RN enclosed in the grid. $\mathrm {V}$ represents the set of cells that intersect at least a line segment within the given sector. Edges in $\mathrm {E}$ are those pairs (*v*, *w*) where *v* and *w* are adjacent cells in the grid and there exists a line segment that intersects both of them. Each edge (*v*, *w*) is labelled with the minimum $\nu ^\mathrm{min}_{(v, w)}$ and maximum $\nu ^\mathrm{max}_{(v, w)}$ speed allowed in the line segment that connects *v* to *w*. We denote by $v_0$ the cell that corresponds to the target LKP and assume that a set of target possible destinations, which we will identify with a subset $\mathcal {W}\subset \mathrm {V}$ of cells, is given. A walk-through of the process of constructing the graph $\mathcal {G}$, applied to a very simple example, is shown in Fig. 4.

**Fig. 5:**
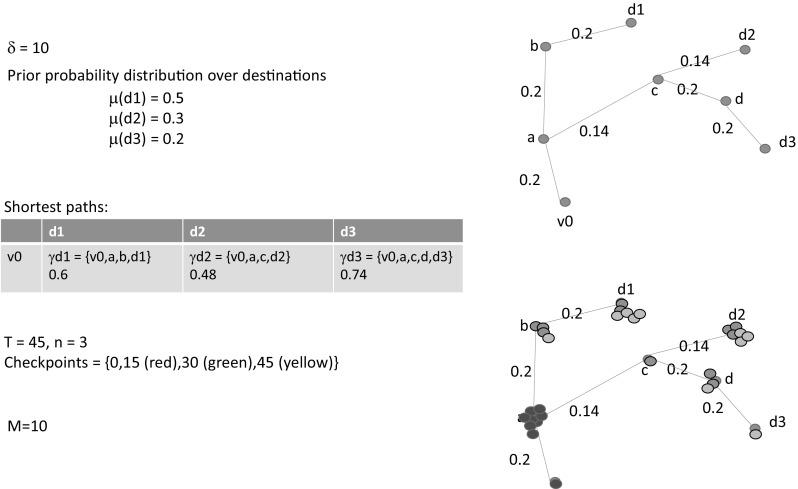
Performing Monte–Carlo simulation of the target movement. On the *left hand side*, the PD over the destinations $d_1, d_2$ and $d_3$ and the shortest paths towards them with their corresponding distances are shown for our running example (see Fig. 4). On the *right hand side*, the shortest paths are shown pictorially together with a representation of how the particles move on these paths over time. Three time check points are selected: 15, 30 and 45. The position of the particles at these times is shown by representing them in different colours: *red*, *green* and *yellow* (Color figure online)

### Probabilistic motion model

We assume that a PD over the destinations $\mathcal {W}$ is given: $\mu : \mathcal {W}\rightarrow [0, 1]$. From the graph $\mathcal {G}$, we calculate the shortest path from $v_0$ to each destination in $\mathcal {W}$ by using Dijkstra’s single-source-shortest-path algorithm, assuming that the travelling time on each edge (*v*, *w*) is $\delta /\nu ^\mathrm{max}_{(v, w)}$. Given $v_0$ and a destination $w \in \mathcal {W}$, we denote $\gamma _w = (v_0, \ldots , w)$ the shortest path from $v_0$ to *w* and *l* the length of such path. Figure 5 shows the PD over the destinations $d_1, d_2$ and $d_3$ and the shortest paths towards them with their corresponding distances for our running example (see Fig. 4), both numerically and pictorially.

The target motion is modelled as a continuous time stochastic process *X*(*t*) that takes values on $\mathrm {V}$ and is described as follows:

- The final destination cell $w \in \mathcal {W}$ is sampled according to the PD $\mu$;
- *X*(*t*) moves from $v_0$ to *w* by following the shortest path $\gamma _w = (v_0, v_1, \ldots , v_l=w)$ and by jumping from $v_k$ to $v_{k+1}$ at the random time $t_k$;
- The jumping time $t_k$’s are iteratively determined according to the following formula: $t_{k+1} - t_k = \delta /\nu _k$, where $\nu _k$ is a uniformly distributed random variable in the interval $[\nu ^\mathrm{min}_{(v_k, v_{k+1})}, \nu ^\mathrm{max}_{(v_k, v_{k+1})}]$.

### Approximation of marginal distributions

*X*(*t*) is a continuous time process, but we want to look at it only at certain time points. Given the mission time interval $[0,\mathrm {T}]$, we establish the time check points $t_0 = 0, t_1, \ldots , t_n$, where $t_{i+1}=t_i+\mathrm {T}/n$. Our goal is to estimate the marginal PD of the process *X*(*t*) on the above checkpoints $t_0 , t_1, \ldots , t_n$. We then use these marginals to generate candidate search patterns, as we will see in the next section.

Estimation of the marginal is performed through standard Monte Carlo Simulation (MCS). More specifically, we consider a set of *M* particles moving in the graph as independent realisations of the stochastic process *X*(*t*). Let $x_j(t)$ be the position of the *j*-th particle at time *t*. We define the approximated distribution of the process *X*(*t*) at time $t_k$ as $q_v^{t_k} = | \ \{j | x_j(t_k)=v \} \ | \ / M$ for $v \in \mathrm {V}$. From the law of large numbers, we know that $q_v^{t_k}$ approximates, for a sufficiently large *M*, the true marginal distributions of $X(t_k)$.

**Fig. 6:**
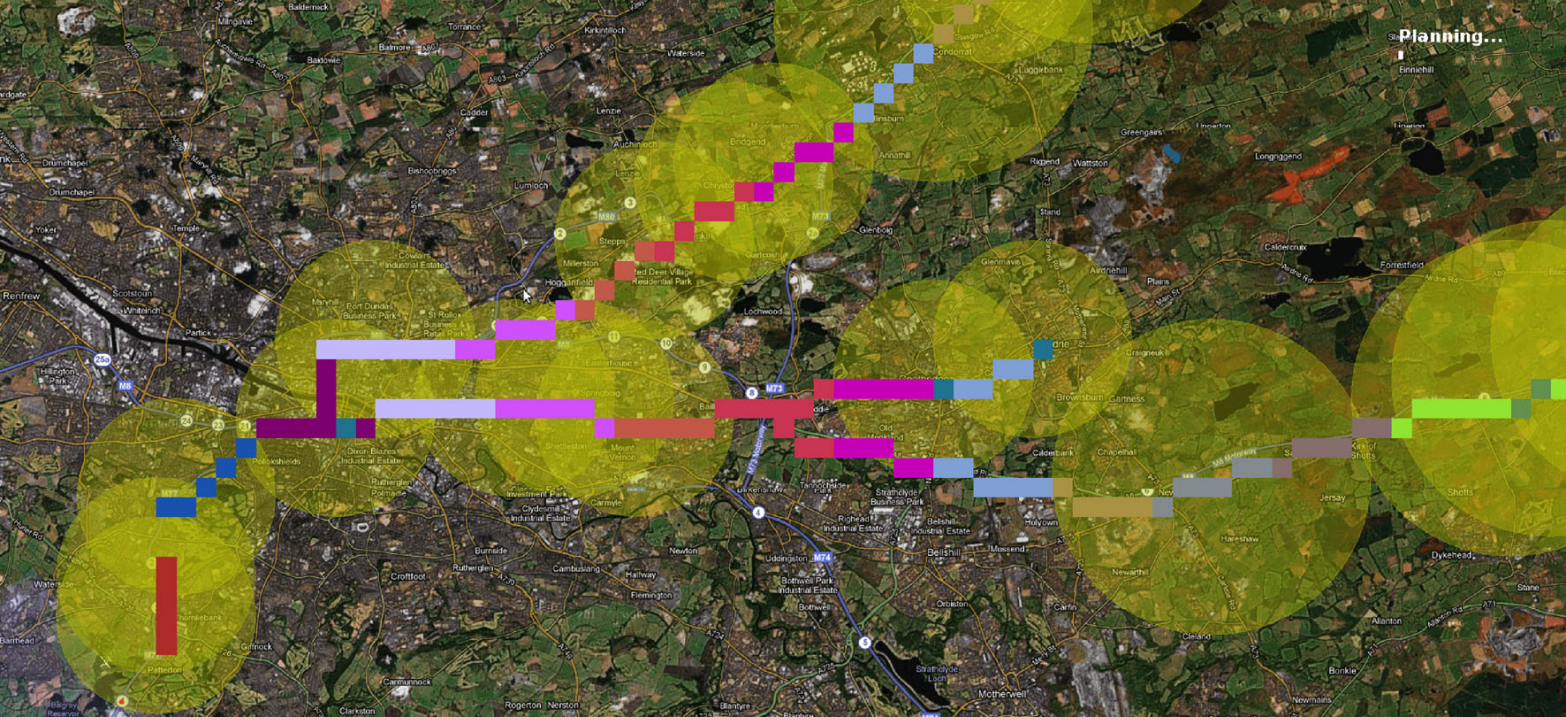
Results of the MCS and candidate pattern generation phase. In each time slice, the cells that accumulate more than one particle are visualised with the *same colour*. For each time check point, the candidate generation algorithm creates one spiral (*yellow circle*) centred around the cell with the highest probability of finding the target within that time (Color figure online)

Figure 5 shows the particles moving over the graph according to the motion model described above and their positions at three different checkpoints. It should be noted that, for the sake of simplicity, we have used a very coarse $\delta$, a small set of checkpoints and very few particles. In real instances, the grid and time discretisation are much finer and the MCS uses thousands of particles (see Sect. 8 for realistic examples).

## Generation of candidate search patterns

Thanks to MCS, our algorithm generates only candidates that bear a high probability of rediscovery, thereby allowing the planner to restrict its reasoning to a limited set of promising patterns. In particular, for each time check point $t_k$, the candidate generation algorithm selects the cell $v_k^*$ that maximises the approximated marginal probability $q_v^{t_k}$ and creates a candidate pattern centred around this cell. More precisely, the centre of the cell $v_k^*$, which we call $c^*$, coincides with the centre of the bounding box that contains the pattern. For spirals and sector search patterns, such a bounding box is a circle and the point $c^*$ is its centre. For expanding square, parallel track and creeping line searches, the bounding box is a square or a rectangle and $c^*$ is the intersection point of the diagonals.

For spirals, expanding square and sector search patterns, $c^*$ is also the entry point of the pattern. The exit point is determined by fixing the outer radius (as specified below) and the number of turns. For parallel track and creeping line searches, the entry point is the rectangle’s upper corner that is closer to the target LKP and the exit point is the bottom corner that is further from it. The number of turns and legs of the patterns are configurable parameters in our system.

We use patterns of two different sizes, small and large. Small patterns cover limited portions of the map, but they do so with great accuracy. On the other hand, large patterns give a broader coverage, but are less accurate. Our algorithm selects small patterns to cover areas in which it is more likely to find the target, i.e. closer to the target LKP, while large patterns are used further away from it. The radius of small and large circles is 2500 and 4000 m respectively and the side of small and large rectangles is 4000 and 9000 m respectively. These are also configurable parameters of our simulation.

As explained in Sect. 4, the particular type of pattern to use to cover an area depends on the specific features of the area. Since spirals, expanding square and sector search patterns provide a more focused coverage than lawnmower searches, our algorithm favours them in urban and suburban areas with a high road density. Parallel track and creeping line searches are used in rural areas or areas of lower road density, where spirals and similar patterns are likely to cover significant areas of little value in the search.

For lawnmower searches, our algorithm selects an orientation that is based on the RN and is aligned to follow the major road enclosed in the pattern.

Figure 6 shows a screenshot of our simulation immediately after the candidate generation algorithm has generated a set of spirals (yellow circles) to cover the most probable cells in the grid at the different time check points from the beginning to the end of the mission.

## Search as planning

Not all the candidate search patterns that have been identified can be executed since the observer has limited resources. The challenge is then to decide which candidates should be selected for execution, when exactly they should be executed and how many times each one should be repeated. We see the task of selecting and sequencing search patterns as a *planning problem*. We assign each pattern a time-dependent *reward*, i.e. a value corresponding to the expectation of finding the target in a search of the area that the pattern covers. Based on the patterns’ rewards, the planner can select a sequence of patterns that maximises the accumulated expectation of rediscovering the target.

This planning problem has some unusual and interesting features. Despite the inherent uncertainty in the situation, the problem is *deterministic*, since the uncertainty arises in the position of the target and, if the target is found, the plan ceases to be relevant. Therefore, the plan is constructed entirely under the assumption that the target remains undiscovered. In what follows, we first discuss why we discarded alternative formulation for our problem and, then, described in detail the knowledge engineering process that we follow to formulate both the planning domains for the UAV and the quadcopter, and the planning problems for SaT missions.

### Modelling using a non-stationary MDP

In our approach, we first use the MCS process to define a discretised reward function associated with the behaviour of the target. Having done this, we then *plan* the behaviour of the observer to accumulate as much reward as possible. Because we separate the models of the target and the observer, we can plan observer actions at a much finer granularity than was used to define the reward function.

**Fig. 7:**
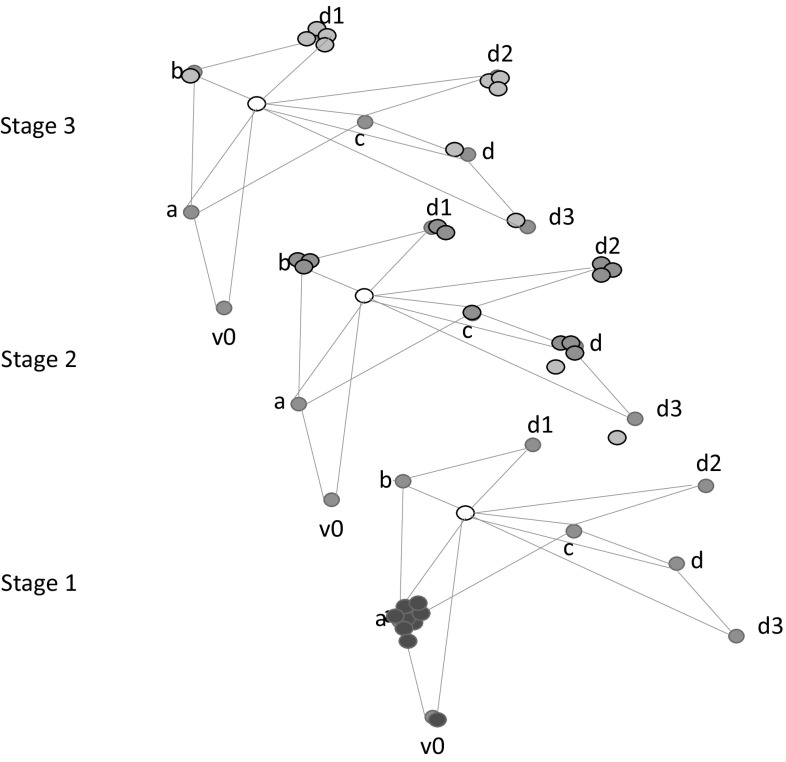
A three-stage MDP capturing our model. The central white state at each stage is the sink. The probability of entering it depends on the likely location of the target at that stage. Here, this is shown by *coloured dots* for consistency with our planning model representation (Color figure online)

An alternative approach is to model and solve a non-stationary Markov Decision Process (MDP) (Boutilier et al. 1999) in which the stages of the process are the $n+1$ checkpoints $\{0,\mathrm {T}/n,2\mathrm {T}/n$, $\ldots \mathrm {T}\}$ at which the location of the target changes. The model would require $n+1$ transition matrices to capture the $n+1$ stages of the process. By contrast with our planning approach in which the target and observer behaviours are separated, in the MDP approach, the behaviour of the two would be combined, so the granularity of the checkpoints would need to be fine enough to allow for timely behaviour of the observer. Figure 7 shows an example non-stationary model. At each stage, the transition model has been extended by the addition of a state in which the target has been found. This state attracts all the reward, so the optimal policy is to reach this state as quickly as possible. Whenever this “sink” state is reached, the search behaviour is terminated and tracking is resumed.

Every transition of the observer would lead either to a state in which a search had been performed without finding the target or to the sink state (in fine-grained models, multiple states might be chained together to represent the commitment to complete a search pattern once started). The best state to visit next is the one with the highest probability of entering the sink state in the next time step. In the MDP model, the probability of entering the sink state from a given state, at stage *t*, is equal to the probability that the target will be identified by the observer searching an appropriately located pattern at stage *t*.

In the two-stage process that we use in our planning approach, our coarse target behaviour model simply allows us to identify when the most reward is likely to be available, with reward tailing off in a Gaussian way on both sides of the peak. This allows the observer to plan to maximise reward by choosing the best times at which to start and end search patterns. To obtain this flexibility in the MDP model, the whole system must be constructed at the finest sensible granularity (e.g.: the granularity of the smallest observer operation). Over a long horizon, this would lead to an infeasible blow-up of the non-stationary model.

This is not the only way to model the search and track problem as an MDP, but the time-granularity issue will always arise in any time-discretised model of the joint observer-target behaviour. We therefore see the planning approach as advantageous in discretising the target behaviour, for convenience, while allowing the observer to plan operations in continuous time.

### Planning domain

To model the SaT domain, we use the language PDDL2.1 (Fox and Long 2003), exploiting the Temporal Initial Literal (TIL) extension of PDDL2.2 (Edelkamp and Hoffmann 2004), further extended with TIFs (Temporal Initial Fluents) (Piacentini et al. 2015).

The basic structure of the domain for the search problem is simple: there is a flight action that allows the observer to fly from one waypoint to another and there are search actions corresponding to the flight patterns. Table 1 displays an example of how search actions are modelled, showing the action doSpiral extracted from the UAV domain. These search actions have a similar form: they have an entry waypoint (?from) and an exit waypoint (?to) and the effect, other than to move the observer, is to increase the total reward (reward), which is the accumulated expectation of finding the target, by a quantity that represents the specific reward associated with that particular pattern (rewardOf ?p). The actions are durative and their duration is fixed in the problem instance to be the correct (computed) value for the execution of the corresponding search. The search patterns can only be executed when they are active (active ?p), i.e. during a window of opportunity that coincides with the period in which the target could plausibly be in the area that the pattern covers. This window is calculated by considering the distance between the pattern’s entry point and the target LKP and the minimum and maximum speeds of the target along the shortest path from the target LKP to the pattern’s entry point.

**Table 1:**
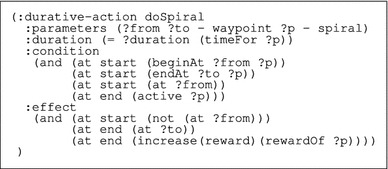
PDDL2.2 specification of the action doSpiral contained in the UAV domain

**Table 2:**
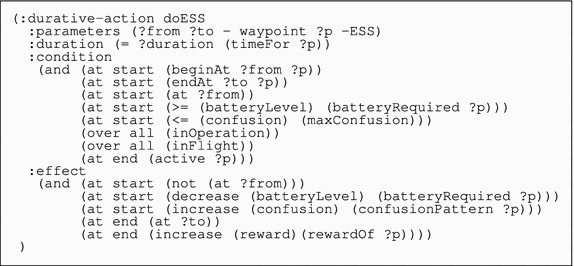
PDDL2.2 specification of the action doESS (do Expanding Square Search) contained in the quadcopter domain

### Modelling UAVs and quadcopters in PDDL2.2

The models for the UAV and the quadcopter share the same structure described above, but differ in some of the aspects relating to their different hardware characteristics.

In regard to the UAV, along with the flight action that allows it to move from one waypoint to another, we have actions corresponding to the following search patterns: spirals, small and large lawnmowers and contour searches, used to get around obstacles. All these actions have the same structure of the action showed in Table 1. Appendix reports the full domain for the UAV.

As for the quadcopter, Table 2 shows the description of the action doESS as an example, while the full domain can be found in the online Supplementary Material.

Since the quadcopter has a more limited flight autonomy and noisier sensors than the UAV, we need to model some of its physical features in the domain in order to produce effective plans for the search problem. In particular, the quadcopter domain contains the following actions: (i) taking-off; (ii) landing; (iii) hovering; (iv) flying between waypoints; (v) performing five types of patterns: parallel track, creeping line, expanding square, sector search and contour search; and (vi) performing a re-localisation procedure, planned for execution when the anticipated confusion of the quadcopter rises above a certain threshold.

The take-off and landing actions are straightforward. The only caveat is that the duration of the take-off action and the related battery consumption, pre-computed and fixed in the problem specification, need to take into account the localisation algorithm initialisation.

The search actions have a structure similar to the corresponding ones in the UAV model. However, given the specific limitations of quadcopters, we need to take care of two additional issues, one relating to the management of the battery life and the other concerning possible failures of the quadcopter’s localisation algorithm in GPS-denied environments, such as indoors and in cluttered outdoor regions. Let us consider this second issue first.

Given their cheap and noisy sensors and their limited payload, quadcopters are often unable to establish their position accurately. Usually, they have enough sensing capabilities to localise themselves in favourable conditions, but not everywhere. The localisation ability varies across the environment, with different environmental features providing different degrees of accuracy (He et al. 2008). For example, localisation based on the Extended Kalman Filter performs well in the vicinity of high-level features such as walls and corners, but the performance starts degrading in moving away from them. To deal with the problem of maintaing good localisation, we incorporate an abstract model of the quadcopter’s sensors and their measurement capabilities into the planning domain and prompt the planner to create plans that are robust to sensor limitations.

More specifically, we use the numeric function confusion to model the level of uncertainty that the quadcopter has accumulated about its state during flight. The confusion level increases when the drone moves away from the visual features required for localisation and is reset when it is close to them. We indicate the set of all search patterns with $\varSigma$ and the set of search patterns that overlap positions in the map where good localisation is ensured with $\varSigma ^{+}$. Let us call $\sigma ^{(t)}$ the pattern $\sigma$ executed at the time *t* and $S = (\sigma ^{(1)}, \sigma ^{(2)}, \ldots ,$ $\sigma ^{(\bar{t})})$ a sequence of patterns to be executed at consecutive times. The confusion function $\mathcal {C}$ for the sequence *S* at time *t* is recursively defined as follows:

1 $$\begin{aligned} \mathcal {C}_t(S) = {\left\{ \begin{array}{ll} 0 &{}\,\,\, \text {if } \sigma ^{(t)} \in \varSigma ^{+} \\ \mathcal {C}_{t-1}(S) + \mathrm{c}(\sigma ^{(t)}) &{} \,\,\,\text {if } \sigma ^{(t)} \not \in \varSigma ^{+} \end{array}\right. } \end{aligned}$$

where $\mathrm{c}: \varSigma \rightarrow \mathbb {N}$ is a function that quantifies the confusion level that results from executing each pattern $\sigma \in \varSigma$. These values are precomputed off-line based on the characteristics of the search region and are specified in the problem instance as well as a safety threshold (maxConfusion) that represents the maximum amount of uncertainty that the drone can accumulate before performing a re-localise action. This action involves flying to a location where the localisation algorithm is known to perform well and re-initialising the state-estimation procedure in that position. The safety threshold is set in such a way that, when it is hit, the quadcopter is still capable of reaching one of the re-localisation waypoints, although partially confused about its own state.

We calculate the confusion level for each candidate pattern as follows. We lay a grid over the area of operations and assign a confusion level to each cell. The confusion is zero in cells that overlap the visual structures in the environment, such as pillars, corners and walls, and increases progressively in moving away from them. In particular, let us call $\mathcal {I}_g$ the set of the cells that provide good localisation and, given a cell *i*, let $dist(i,\mathcal {I}_g)$ be the distance between *i* and $\mathcal {I}_g$, i.e. the length of the minimum path between *i* and one of the cells in $\mathcal {I}_g$. The confusion associated with a cell *i* is then $J * dist(i,\mathcal {I}_g)$, where *J* is a constant. The confusion associated with a pattern $\sigma$ is obtained by adding up the confusion levels of the cells included in the pattern: $c(\sigma ) = \sum _{i \in \sigma } J * dist(i,\mathcal {I}_g)$.

The confusion level is manipulated by the actions corresponding to the search patterns. They have a condition that checks that the current confusion level is below the threshold and an effect that increases the level of confusion to an extent specified in the problem instance for that pattern. When reasoning about possible trajectories for the drone, the planner tends to select patterns that keep the confusion level below the threshold by favouring waypoints that maximise the localisation accuracy.

In principle, our definition of confusion is related to the sensor uncertainty field introduced in Takeda and Latombe (1992), which is a mapping from locations to expected information gain, where locations with high information gain correspond to locations that generate sensor measurements that maximise the localisation accuracy of the vehicle. In our case, we estimate the information loss instead of the information gain. However, we do not use probabilistic methods to build the field, but we discretise the problem in space and construct a coarse-grained confusion map by simply attributing a level of confusion to each cell based on the distance from the cells where optimal localisation can be achieved. Our attempt to incorporate predicted measurements into the drone’s decision making is similar to the work in He et al. (2008), where a motion planning algorithm is presented that takes into consideration how well the quadcopter can localise itself along that path. Similar to our case, in Fox et al. (2012), a confusion variable is used to keep track of how far a AUV moves without crossing the edge in a patch following task. To obtain good measurements, the AUV needs to cross the edge several times while exploring a patch. The problem is finding trajectories for the AUV that are as short as possible, but keep the confusion level within a small bound.

Let us now consider the second issue relating to the battery of the drone. As energy is a critical resource for the quadcopter, we include a model of its battery in the planning domain (batteryLevel). Instead of modelling the continuous change of the battery level directly, we use discretised durative actions in combination with numeric step-function updates. The quadcopter’s manoeuvres have a condition *at start* ensuring that the available amount of energy is sufficient to perform the manoeuvre and an instantaneous effect *at start* modelling their energy consumption. The energy required for the different manoeuvres has been estimated off-line via actual testing with the quadcopter and is fixed in the problem instance.

We use two alternative domains for the quadcopter. In the first one, the quadcopter’s maximum flight time is constrained by its battery life. To establish a planning horizon for our problem corresponding to the total flight time, we use an *envelope* action operate around all the activities of the quadcopter: it must start before the take-off as well as it must finish after landing and its duration is bounded by the total flight time. In our second domain, SaT missions are subdivided in different chunks. The quadcopter can use the entire battery charge in each chunk and then recharge the battery to perform the next part of the mission. We use a discretised durative action recharge to model the recharge of the battery, which only makes new charge available at the conclusion of the action, so that the charge gained during the action cannot be exploited until after the recharging process is complete.

### Planning problem

The initial state of a planning problem contains all the candidate patterns from which the planner chooses the ones to execute. They are the objects of the planning instance together with the waypoints of interest in the map. Such waypoints are the target LKP, called origin, and all the entry and exit points of the patterns.

Each candidate search pattern is assigned the following information: (i) a fixed duration, which is the computed value for the execution of the corresponding search; (ii) an opportunity window, which specifies when the pattern is active; (iii) entry and exit waypoints; and (iv) a *reward*.

All these pieces of information are computed during the candidate generation phase based on the geometry of the pattern, the map of the environment and the motion models of the target and the observer. They are then compiled into a planning problem automatically and fed into the planner together with the domain model.

While it is straightforward to compute the first three pieces of information, the reward function is slightly more complex. The reward is the product of the detection probability $p_d$, i.e. the probability that the observer sees the target while passing over it, and the probability $p_a$ that the target enters the area being searched.

The detection probability $p_d$ is affected by several factors, such as the type of search pattern, the camera used, the direction in which the target has been travelling and the characteristics of the area traversed by the target (e.g. terrain types and RN). We assume that this probability is fixed for a particular terrain type and use the following values: 0.8 for rough terrain, 0.6 for mountainous, 0.2 for urban, 0.5 for sub-urban and 0.25 for forested. This is clearly a simplification since these values combine the effects of terrain, RN structure, traffic, speed of target and lighting conditions into a single factor that is assumed constant for the whole of a region of identical terrain. These values are configurable parameters in our simulator and can be changed if desired.

The probability $p_a$ that the target enters the search area is estimated based on the RN structure and the distance from the target LKP. In particular, let $G = \{g_0, \ldots , g_k\}$ be the cells covered by a pattern $\sigma$ and $rw: G \rightarrow \mathbb {N}$ the cell reward function defined as follows: $rw(g) = (1+rd) / (1+d)$, where *rd* is the number of roads passing through *g* and *d* is the distance between the centre of cell *g* and the target LKP. The probability $p_a$ is obtained by summing up the reward of the cells that are covered by $\sigma$ and dividing this sum by a normalisation factor *f* that maintains $p_a$ between 0 and 1: $p_a = ( \sum _{g\in G} rw(g) ) / f$.

Overall, the reward of a pattern is proportional to the number of roads and the terrain type in the pattern and inversely proportional to the distance from target. Hence, patterns that cover many roads are more rewarding and patterns that are distant from the target LKP less rewarding.

**Fig. 8:**
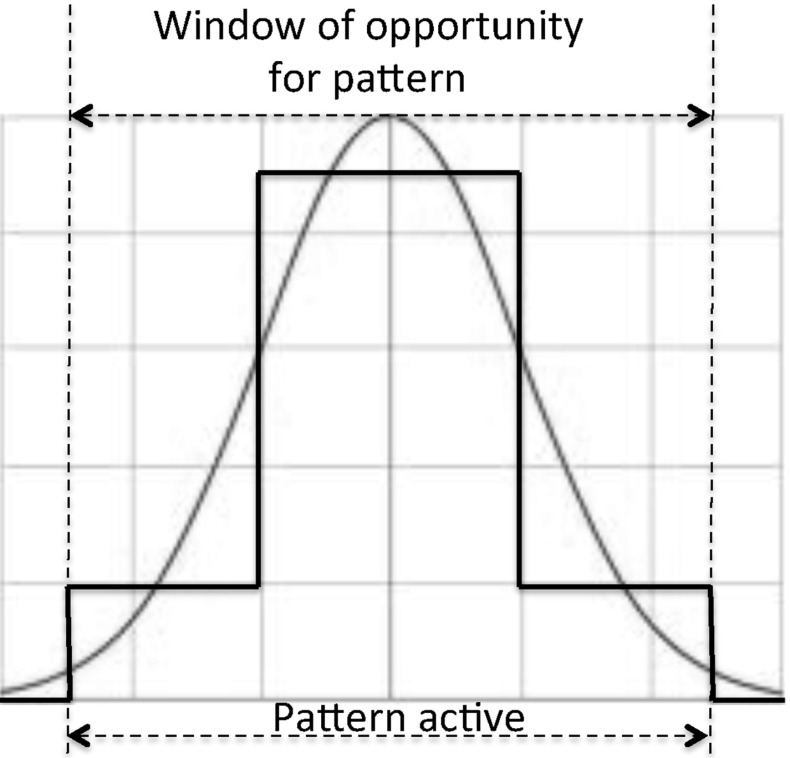
Time-dependent reward function

Since we are searching for a moving target, the reward is a time-dependent function. No reward is assigned until the target has plausibly arrived in the area covered by the pattern or after the target is deemed likely to have left the area. Between these extremes, the reward is modelled as a step function that approximates a lifted Gaussian distribution (Fig. 8). It increases when the pattern becomes active, it increases further when the target is considered most likely to be in the pattern and then it decreases until the end of the useful life of the pattern. The underlying probability of detecting the target peaks at the point where the target is assessed to be in the centre of the search pattern. We use a unimodal distribution because we assume that the target moves towards its destination using the most efficient path to reach it, never revisiting locations that it has already traversed. The variance is generated by uncertainty about the precise path, environmental conditions and precise speed of the target.

To ensure that the planner does not exploit search patterns when there is no reward associated with them, the patterns are only made active during the period when the PD is positive. To achieve this, we use Timed Initial Literals (TILs) that are asserted and retracted at the appropriate times and Timed Initial Fluents (TIFs), which are numeric assignments associated with time points specified in the initial state. Reward for a specific pattern is therefore modelled as a series of step functions, each asserting the new reward value for the interval until the next function applies, while TILs assert or retract the availability of the search pattern at the start and end of the relevant window. The number of steps in the discretisation can be increased, but we have found empirically that four step functions is a good compromise between accurate representation of the reward function and efficient management of the functions in the planner.

**Fig. 9:**
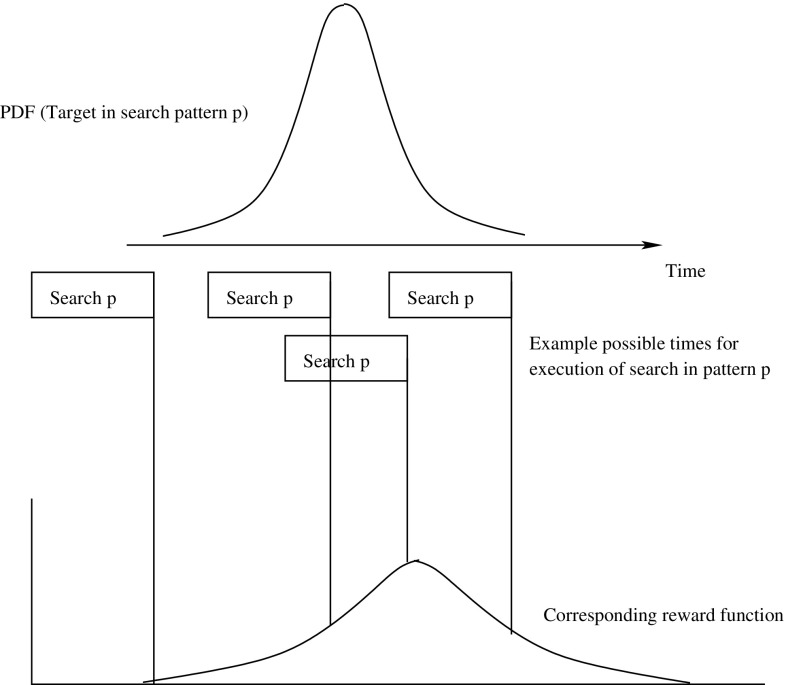
Offset between underlying probability of target being within the search pattern and the corresponding reward function

An important detail of the model is that the reward is awarded as a discrete increment at the *end* of a search action, but it is, in reality, accumulated continuously over the duration of the search action. The end point of the action adds the current recorded reward value to the accumulated total reward, which means that the value available at this time point actually represents the accumulated reward for having executed the search over the duration of the search ending at this point (it is proportional to the integral of the PDF over the interval of the search). Hence, the reward function used in our model is offset from the period over which the model predicts the target is most likely to be in the pattern area, according to the duration of the search pattern (see Fig. 9).

A fragment of the problem specification for the UAV domain is presented in Table 3.

For problems involving the quadcopter, each search pattern is also assigned a battery usage and a confusion level, which measures the localisation accuracy of the vehicle in the area covered by the pattern and depends on the physical characteristics of the area. They are both computed off-line during the candidate generation phase based on the characteristics of the vehicles and the search area as well as simulation parameters such as weather conditions. For example, for calculating the use of battery, we have two possible settings, light and strong wind.

Along with the initial state, the problem specification contains a description of the goal. In the UAV case, the goal is that the reward must be greater than zero and the plan metric measures the value of the plan in terms of the accumulated expectation of finding the target. This is specified as reported in Table 4.

**Table 3:**
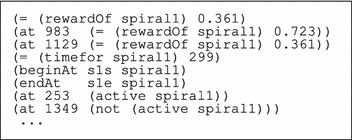
Small fragment of the initial specification for the UAV domain

Spiral 1 has a duration of 299 time units and is active between time point 253 and time point 1349, which coincides with the interval during which the target might plausibly be in the area covered by the pattern. The reward is higher than zero only during this time window. It peaks between time point 983 and time point 1129, when it is equal to 0.723, and is equal to 0.361 elsewhere

**Table 4:**
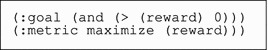
Goal specification for the UAV domain

**Table 5:**
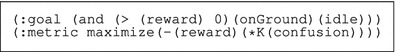
Goal specification for the quadcopter domain

In the quadcopter case, the planning problem has just one goal, specifying that the vehicle should be on the ground at the end of the operations. However, if relevant for a certain mission, one can set out a number of waypoints that the vehicle needs to visit before landing. We use a plan metric that measures the value of the plan in terms of the accumulated expectation of finding the target and the level of confusion. Clearly, our objective is to maximise the reward and minimise the confusion level, so we use the metric specified in Table 5, where the parameter K is chosen based on the desired trade-off between the reward and the confusion level.

**Table 6:**
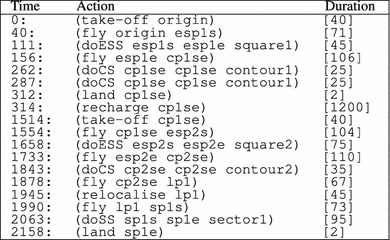
A plan generated by Optic for the quadcopter

The columns indicate: execution time, action to perform and action duration

### Planning mechanism

We exploit the period in which the observer tracks the target predicted location to perform planning. We use a temporal metric PDDL planner planner called Optic (Benton et al. 2012) (Optimizing Preferences and TIme-dependent Costs) to build plans for the observer.^1^ Optic is a temporal planner for use in problems where the cost function is not directly linked to the plan make-span, but can be expressed as a function of metric fluents. It builds on the planner popf (Coles et al. 2010) and combines grounded forward search with linear programming to handle continuous linear numeric change. Optic performs anytime, cost-improving search: it finds a first solution very quickly, since the plan with just one pattern is already a feasible solution, but it then spends the additional time improving on this solution by adding further manoeuvres to the plan or by trying different collections of manoeuvres. The search uses a weighted-$A^\star$ scheme with steadily changing weights in a tiered fashion. The plans produced are monotonically improving, so the final plan is selected for execution. We use a time-bounded search limited to 10 s because we are in a time-critical situation (although this value is also a configurable parameter). We use Optic because it is very fast at producing its first solution and provides an any-time improvement behaviour. Although the planner LPG-TD (Gerevini et al. 2004) offers some similar characteristics to Optic, it does not handle TIFs and, in consequence, cannot be used in our case.

Table 6 shows an example of a plan generated by Optic for the quadcopter domain.

All the plan that are outputted by Optic are single threaded and are dispatched via a simple controller, action by action, to the vehicle. When the target is found or at the conclusion of the plan, the observer abandons the mission.

## Experimental results

We demonstrate the viability of our hybrid approach to SaT by providing two different implementations of our technique: one, in simulation, features a fixed-wing UAV involved in a complete SaT mission (Sect. 8.1) and the other uses the Parrot AR.Drone2.0 quadcopter in the search phase of a SaT mission (Sect. 8.2).

### Simulation experiments

The UAV simulator was built in consultation with our industrial collaborators at BAE Systems (Bernardini et al. 2013) and is intended to provide an appropriately abstracted view of the problem. The main abstraction is that we assume the control problem for the UAV solved.

The simulated UAV is equipped with imaging systems that allow the target to be observed and that is susceptible to error. The detection probability on each observation cycle (which can be considered as a ‘frame capture’ by the imager) depends on: terrain, speed, discrepancy between anticipated and actual target positions and the imaging system mode. As explained in Sect. 7.4, this detection probability is compiled into our reward function. The imager has two modes: wide-angle, used to increase the area being scanned when the target is not currently observed at the cost of a lower probability of successfully observing the target, and narrow-angle, used during tracking, in which the viewing area is reduced, but the probability of detecting the target is higher. The effect of terrain is to reduce the probability of spotting the target in urban, forested and mountainous areas, while in rough or open rural areas the probability is higher.

The area of operations is a part of Scotland about 100 kilometres square. Terrain types were defined by hand, along with an approximate RN for the major roads and rural minor roads. The target follows a path acquired using Google Maps, using a selected (configurable) origin and destination. This information is also used to decide what speed is appropriate for the target, based on distance between waypoints in the route proposed by Google Maps and terrain type.

#### Simulation steps

**Fig. 10:**
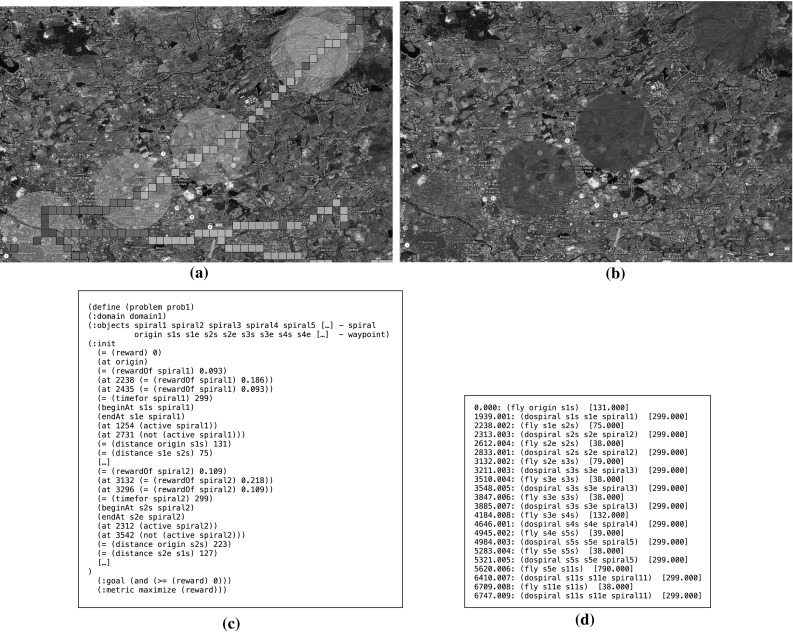
Screenshot **a** shows a fragment of the initial state: *circles* are the spirals that the planner will consider. Spirals are constructed around the cells that carry the highest probability of rediscovering the target. *Small squares* indicate the cells in the grid where the particles accumulates during the MCS. *Different colours* represent particles at different time check points. Screenshot **b** shows a few of the search patterns that have been selected by the planner for execution. The intensity of *red* used in the plan indicates repetitions of a search pattern (more intense *red* implies more executions).  **c** and **d** shows fragments of the problem and the plan specifications respectively (Color figure online)

During the tracking phase of the SaT mission, the UAV simulator follows the target by spiralling over it. Once the target has been lost and during the 10 s in which the UAV tracks the predicted location of the target at low confidence, the simulator runs MCS and synthesises a set of candidate search patterns (Fig. 10a). Then, it runs the planner to generate a plan (Fig. 10c, d), which is then dispatched to and executed by the UAV (Fig. 10b).

The goal of our SaT simulation is to demonstrate how planning can successfully underpin autonomous SaT missions. Given that, in this work, we do not tackle dispatching, monitoring, executing and replanning issues. Our dispatcher simply initiates the next action in the plan in simulation. We assume that there are no failure in the control and execution of the actions, so no replanning due to failures is needed. The interested reader can refer to our previous work (Cashmore et al. 2015) to see how dispatching, monitoring and executing tasks can be integrated with planning within a ROS framework.

As for the MCS, we follow the procedure presented in Sect. 5, but incorporate in it additional assumptions that are specific to our simulator. We assume that the RN is characterised by three types of roads: motorways (the fastest roads), A roads (the main routes between towns) and B roads (the smallest of the three). The minimum speed on all these roads is: $\nu ^\mathrm{min} = 20$ mph. The maximum speed $\nu ^\mathrm{max}$ is 70 for motorways, 60 for A roads and 30 for B roads. After running MCS, the simulator visualises the most probable cells in the map at the different time check points (Fig. 10a). In each time slice, the cells that accumulate more than one particle are visualised with the same colour. Different colours correspond to different time slices. As expected, the particles follow the main roads and cluster around cities.

After experimenting with different granularities, we now adopt a grid square size of $\delta = 500$ m. The graphs extracted from the RN have around 30,000 nodes and 18,000 edges, on average. We choose as the set of possible destinations the first 15 most populated cities in Scotland and assign them equal probability. We generate $M = 10,000$ particles and, since the total mission time for our application is about 1 h, we consider 17 time check points spaced 150 s apart. Considering that the rate of approximation in the MCS is of the order $1/\sqrt{M}$ (because of the central limit theorem), and that the maximum value of the marginal distribution of each $X(t_k)$ will be above 1 / 15, $M = 10,000$ is a sufficiently large ensemble for the simulation.

We have found that, on average, Optic produces around 6 plans in its 10 second time window per problem instance, and the last of these plans is always selected for execution. The plans contains on average 15 patterns and 15 fight actions to route the UAV from one pattern to the next one.

The simulation tool offers various opportunities for interaction, including redirecting the target, repositioning the observer, speeding and slowing the simulation and modifying the parameters that govern spotting probabilities, flight dynamics and the target behaviour.

#### Results and discussions

We conducted a series of experiments to assess the performance of our hybrid approach to SaT by using the UAV simulation. As we have seen in Sect. 7.1 and will further discuss in Sect. 9, it is difficult to directly compare our approach against purely probabilistic methods, as the assumptions behind them are very different from ours. Here, then, we assess our strategy against two comparable ones: a fixed policy, which is used as a baseline for evaluating the benefits of a plan-based approach, and a plan-based approach that does not use MCS.

**Fig. 11:**
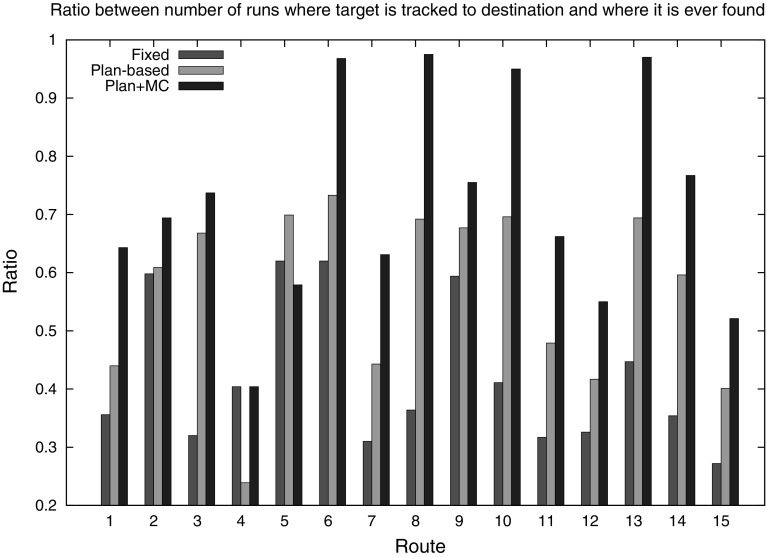
Proportion of successfully tracked targets

**Fig. 12:**
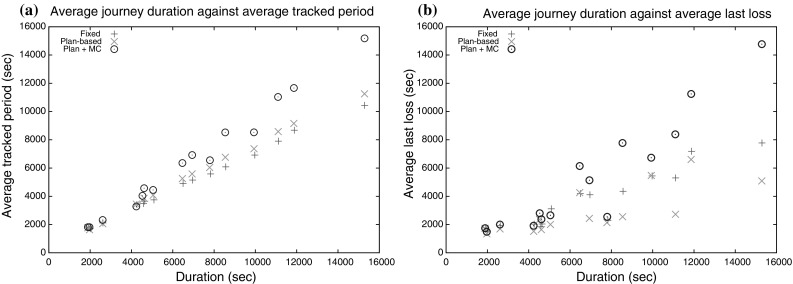
**a** Plots the average time over which the target was tracked to destination against the average journey length for the 15 routes and the three strategies. **b** Plots the average time of the last loss of the target against average journey length for the 15 routes and the three strategies

Our industrial collaborators proposed the fixed policy, which is used in several real-world applications. When the UAV loses the target, it tracks its predicted location for 3 min. If it has not found the target, it executes a fixed sequence of patterns: first a spiral around the target LKP and then a large lawnmower pattern (20 km square).

Though broadly similar, the plan-based policy (Bernardini et al. 2013) differs from our strategy in one crucial way: the target PD is not generated via MCS, but it is constructed by hand based on the features in the environment, without taking into account the target motion model. In particular, the PD is based on the density of roads across the search area, which is measured by using a fine-mesh grid and counting the number of significant roads within each grid cell, the terrain type and the distance from the target LKP. The distribution decays linearly with distance from the origin and is weighted by values for terrain type and RN density. Plan-based SaT may find good policies for problems with predictable target behaviours, but it struggles with serious inaccuracies when the target acts in a more sophisticated way by neglecting its physical motion in the environment.

Both for our strategy and for the plan-based one, we use a configuration that tracks the predicted location of the target for the same period as the fixed policy, before planning and executing a search plan. We generated 15 routes and executed the simulation on each route 1000 times (the simulation has a non-deterministic spotting model and target behaviour), for each of the 3 strategies (a total of 45.000 runs). The simulation begins with the target undetected, but in the search arc of the observer. In a small number of runs the observer fails to detect the target in the very early stage. Our simulation does not use a search plan in this first stage, so failure at this point leads to an early abort. We discount these runs (less than $0.5\,\%$) in our analysis.

Figure 11, which shows the proportion of runs in which the target was tracked to its destination, demonstrates that our hybrid strategy is consistently better than the other strategies. The fixed policy has an overall success rate of around $42\,\%$, the plan-based policy without MCS has overall success rate of around $56\,\%$, while the plan-based policy with the MCS yields better than $72\,\%$ success rate. Our hybrid approach performs particularly well when the target drives towards destinations far away from the origin as the MCS allows us to make better predictions than the other strategies.

Figure 12a shows the average time that the observer tracks the target plotted against journey duration for the three strategies. It can be seen that the hybrid technique produces the best performance, while the static policy is generally the weakest. Figure 12b shows the average time at which the target is lost for the last time, plotted against the duration of the journey. When the plan-based policy is used in combination with the MCS, the time at which the target is lost for the last time increases with the increase of the duration of the journey. This is because, on average, our strategy is capable of tracking the target for a longer time.

Our results clearly demonstrate the benefit of using planning in combination with MCS both with respect to fixed policies, currently being employed in real-world scenarios, and with respect to a plan-based approach in which the PD maps are not accurate enough to allow planning to express its full power.

### Real-world experiments

As a proof of concept that our approach can be successfully deployed in a real robotic platforms, we demonstrated it on a quadrotor helicopter, the Parrot AR.Drone. This demonstration is extensively described in Bernardini et al. (2014), but we include a summary here for completeness. The AR.Drone is a low-cost and light-weight quadcopter that has been increasingly popular in research organisations as an affordable test platform (Bills et al. 2011; Engel et al. 2012; Graether and Mueller 2012). It is composed of a carbon-fibre tube structure, plastic body, removable hulls, high-efficiency propellers, four brushless motors, sensor and control board, a high-resolution front facing camera and a bottom facing camera used for stabilisation.

To allow the AR.Drone to carry out a SaT mission autonomously, we combined the abstract deliberative skills illustrated in Sect. 7 with low-level control and vision capabilities. In particular, for the tracking phase, we implemented tag recognition (we assume that our target is identified by a specific tag) and tag following. For the search phase, since the AR.Drone provides built-in low-level control for robust flight, we focused on high-level control only. Our implementation provides capabilities for localisation, autonomous navigation and compensation for drift. The navigation system that we use is composed of: a monocular SLAM implementation for visual tracking, an Extended Kalman Filter (EKF) for data fusion and prediction, and a PID controller for pose stabilisation and navigation.

**Fig. 13:**
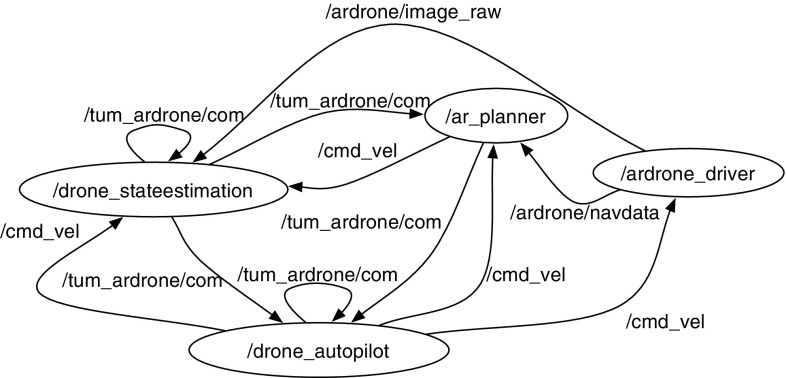
ROS nodes and topics for the search phase of a SaT mission

We implemented our application within the Robot Operating System (ROS) framework (Quigley et al. 2009) (Fig. 13). We exploited existing packages for implementing our SaT application, in particular: ardrone_autonomy,^2^ which is a ROS driver for the Parrot AR.Drone, and tum_ ardrone 3 (composed of drone_autopilot and drone_ state estimation), which implements autonomous navigation and figure flying. We implemented the ROS node ar_planner, which wraps the Optic planner and allows us to integrate it with the rest of the system. This node is invoked by the tag following node when the target is lost. Based on real-time sensory data (current yaw, pitch, roll, altitude, battery state and 3D speed estimates) and information on the target motion model, the ar_planner: (i) runs MCS and builds a planning problem; (ii) calls the Optic planner with a time limit of 10 s; (iii) upon receiving the plan, translates the actions of the plan into corresponding commands specified by using the scripting language provided by the ROS node drone_autopilot; and (iv) sends the translated plan to the node drone_autopilot, which then imparts the commands directly to the ardrone_driver node.

#### Flight tests

We conducted a series of real-world experiments to assess whether the planner is capable of generating effective plans for the drone and whether the drone is able to fly them accurately to completion. Flight tests were performed indoors in a room of dimensions 20.5 m (l) $\times$ 8.37 m (w) $\times$ 5.95 m (h). The experiments pertain to the search phase of a SaT mission only. They were carried out by using the ROS architecture shown in Fig. 13 and the AR.Drone 2.0.

We employed the following procedure to run our tests: we manually generated several planning problems and fed each problem, together with the drone’s domain model, into the ROS architecture shown in Fig. 13. Manually writing the planning problems was a time-consuming task. The initial state describes the candidate search patterns by assigning them the following information: the time and battery needed for flying the pattern, the drone’s degree of confusion after executing the pattern, a time window in which the pattern is active and a reward step function within this window. To write realistic initial states, we manually created several search patterns for the drone with suitable dimensions for our room and ran extensive flight tests of such patterns to gather reliable information about their duration and the battery usage. We created several versions of each pattern with different dimensions, ran each pattern ten times and annotated the average time and energy consumed by the pattern. The results obtained for one version of each patterns are reported in Table 7. The take-off (1 m) and PTAM map initialisation take approximately 40 s and 10 % of battery, while landing (1 m) takes 5 s and 2 % of battery.

**Table 7:** Characteristics of patterns PTS-1, CLS-1, ESS-1 and SS-1

	PTS-1	CLS-1	ESS-1	SS-1
Area (m*m)	4*3	1*4	4*4	3*3
Perimeter (m)	19	9	20	29
Time (s)	50	40	45	95
Battery (%)	10	15	5	10

Since we run our experiments indoors and in a rather limited space, we did not have issues with the localisation algorithm and, in consequence, we used the values of $J=1$ and $K=0$. In this case, although the objective function does not take confusion into account, confusion remains important in effectively solving the problem because it needs to stay below the threshold for a pattern action to be applied. If the confusion level drops under the threshold, re-localisation is performed. If it fails, as well as if any other failure occurs at execution time, the UAV abandons the mission and a new plan is created from scratch. This choice reflects our focus on planning in this work, rather than on dispatching, execution and replanning. Our previous work can be consulted for these topics Bernardini et al. (2014), Cashmore et al. (2015).

As for the planning phase, on average, Optic produces 8 plans per problem instance in its 10 second window.

**Fig. 14:**
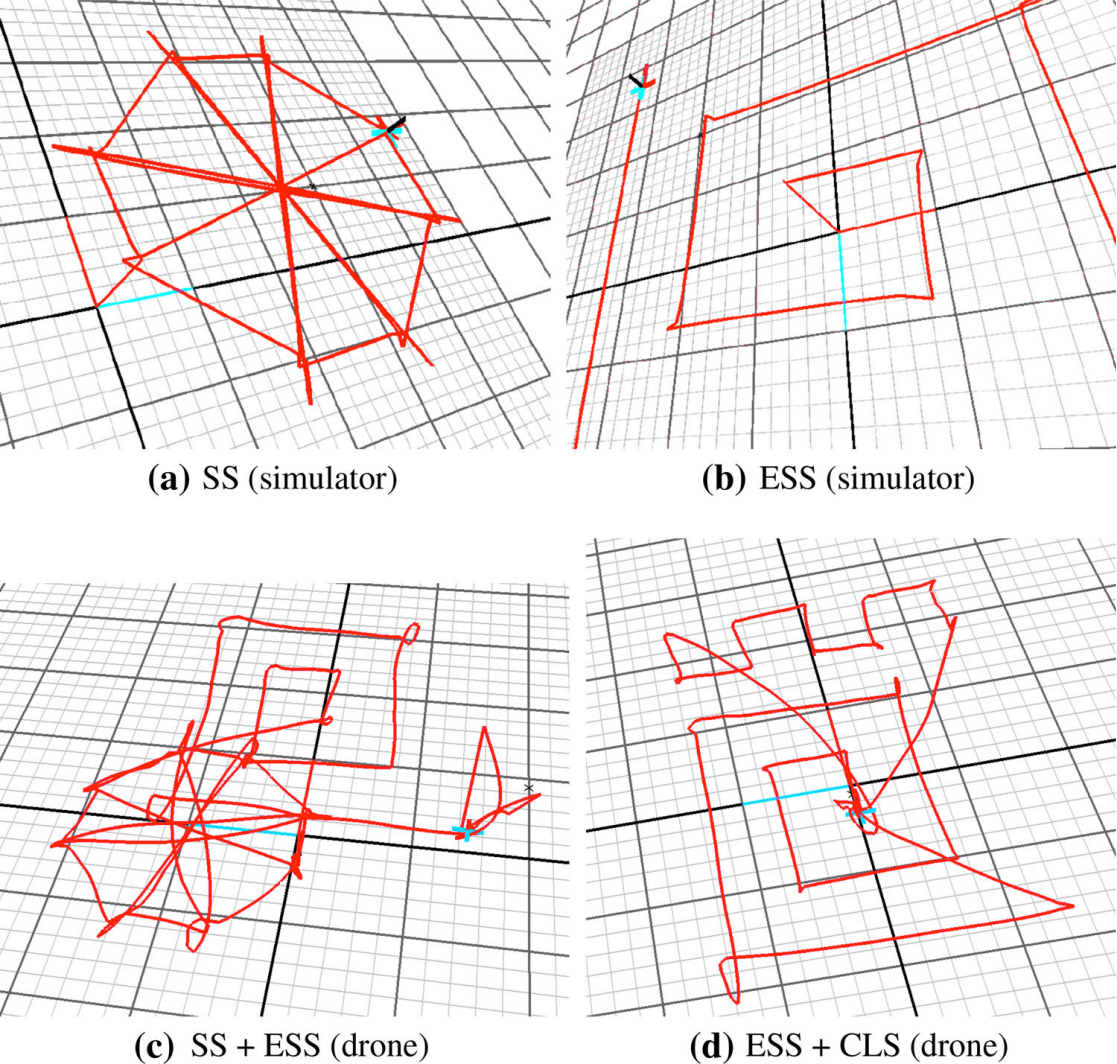
A comparison between the figures in the *above* and *bottom rows* shows that our drone is able to execute the plans generated by the planner very accurately. **a** SS (simulator). **b** ESS (simulator). **c** SS + ESS (drone). **d** ESS + CLS (drone)

When we made the drone execute the plans produced by the planner, we observed that the drone is able to fly all the figures very accurately. Figure 14 shows, in the top row, the execution of a SS pattern and an ESS pattern by the drone’s simulator and, in the bottom row, the execution of two fragments of a plan involving combination of patterns by the real drone. By comparing these pictures, it appears that the drone can execute the planned manoeuvres very precisely.

## Related work

SaT has been extensively studied over the course of many years and by different research communities. In this section, we give an overview of the main approaches to SaT.

### Theory of optimal search and SAROPS

he first practical issues relating to search for a lost target were posed by B. Koopman in the US Navy during World War II (Koopman 1946) and revolved around providing efficient methods of detecting submarines. Search theory techniques were then used by the US Navy to plan searches for objects such as the H-bomb lost in the ocean near Palomares, Spain, in 1966 and the submarine Scorpion lost in 1968 (Richardson and Stone 1971). In the same years, theory of optimal search emerged as branch of operations research and focused on stationary targets (Stone 1975).

Since then, there has been extensive work in theory of search and Coast Guards around the world currently use tools based on it to plan SaR efforts. In particular, the systems CASP (Computer Assisted Search Planning) (Richardson and Discenza 1980) and its successor SAROPS (Search and Rescue Optimal Planning System) (Kratzke et al. 2010) have been used by the US Coast Guard since 1974 for SaR operations involving lost objects at sea and are based on theory of optimal search as developed by Stone (1975).

SAROPS is composed of two main subsystems: (i) the simulator, and (ii) the planner. The simulator produces a time-dependent PD for the target location using MC particle filtering. The system fuses information about the object in distress, provided by human operators, and environmental data, provided by the Environmental Data Server, which gathers environmental data from government and private sources concerning winds, currents, cloud cover, drifters, weather and visibility conditions. Based on the target PD and a collection of available search and rescue units (SRUs), the planner assigns one rectangular search pattern (lawnmower) to each SRU, which then proceeds to execute the pattern. Each SRU executes only one pattern and there is no routing of a vehicle from one pattern to another. The planner seeks to maximise the probability of discovering targets by placing the rectangles intelligently: using an iterative strategy, it tries different combinations of rectangles for the SRUs until a pre-determined period of time has elapsed and, then, reports the best solution. If no SRU finds the target, the simulator generates a new PD by incorporating information about motion (drift) and about the previous unsuccessful search and then the planner generates a new set of rectangles. This loop is not automated, but it involves the presence of a human who always coordinates the two systems and supervises the entire process.

The SAROPS system has a number of similarities with our technique. As in SAROPS, we also use MCS to develop a prior PD for the target’s location, although we do not regenerate the PD at each step. In contrast to our approach, the SAROPS planner works with a one-step lookahead horizon, has no notion of time-dependent reward and does not incorporate any long term strategic reasoning. While SAROPS provides a method for carefully orientating the patterns to cover the search area, we concentrate on organising a sequence of patterns to be explored over time. It would be interesting to compare our strategy against SAROPS, but at the time being this comparison cannot be performed since SAROPS is not in the public domain. However, we^4^ conjecture that, for stationary targets, the careful positioning of patterns provided by SAROPS would pay off. On the other hand, when only a limited number of vehicles are available, which need to be routed from one pattern to another, and when the probability of detection over time needs to be maximised, our approach would outperform SAROPS as it benefits from ordering patterns over a long horizon and from time-dependent rewards.

### Probabilistic approaches

Originally, the two areas of searching and tracking were considered separately. Over the last 10 years, however, the field of probabilistic SaT has evolved rapidly and a unified approach to SaT has emerged (Furukawa et al. 2006).

The probabilistic approach to SAT relies on the use of Recursive Bayesian Estimation (RBE) techniques that recursively update and predict the PD of the target location over time, under the assumption that the prior distribution and the probabilistic motion model of the target are known (Bourgault et al. 2006). Although Bourgault et al. (2004) discuss a number of possible constraints that can impact the target motion model (obstacles, force fields and terrain), the target is usually assumed to be subjected to external disturbances and not to move on the basis of its own intentions.

RBE techniques work in two stages, the update and the prediction. The update stage calculates the posterior distribution of the current state given a prior estimation of the state and a new observation. The prediction stage calculates the PD of the next state using the posterior distribution and the target’s motion model. Since the implementation of these two stages is computationally expensive, several approaches have been explored to compute them efficiently, including grid-based methods (Bourgault et al. 2006), particle filters (Chung and Furukawa 2006), element-based techniques (Furukawa et al. 2007) and hybrid particle-element approaches (Lavis and Furukawa 2008).

The search control problem is solved in a greedy fashion over a very short planning horizon (typically, a one-step lookahead). This myopic planning approach is used to control the computational cost of the technique, which quickly becomes intractable as the number of lookahead steps, the size of the search area, or the number of dimensions of the search space, increases. A unified objective function is used for both search and tracking, allowing a vehicle to switch from one mode to the other while maintaining information gained during the previous phases.

Probabilistic-based SaT has proven successful for problems involving stationary targets or targets moving in small geographical areas, simple motion models, static search spaces and short-term missions. However, when these assumptions are not satisfied, RBE techniques perform poorly due to the high computational cost of accurately maintaining a large state space that includes all the possible positions of the moving targets.

Lately, modern approaches to planning with incomplete state information, which are based on Partially Observable Markov Decision Process (POMDP), have been applied to SaT, both for single targets (Hsu et al. 2008) and multiple targets (Bertuccelli and How 2006). In He et al. (2010), the authors present an online, forward search, planning-under-uncertainty algorithm for the road constrained target-tracking problem. In this work, the agent’s belief of each target’s pose is represented as a multi-modal Gaussian belief and this parametric belief representation is exploited to compute the distribution of posterior beliefs after actions are taken. This analytic computation allows the planner to search deeper by considering policies composed of multi-step action sequences. Deeper searches are beneficial as they result in keeping the targets well-localised for longer periods. This technique has proven successful for small geographical areas, but has not been tested yet on larger regions.

Considerable amount of work has been devoted to devising efficient path-planning methods for UAVs involved in search-and-rescue operations. In Lin and Goodrich (2014), for example, the authors consider the problem of wilderness search and rescue where mini-UAVs are used to locate missing persons. They propose a new family of path-planning algorithms that use a spatial representation, the task difficulty map, to model the sensor detection probability and reason about that during planning as well as a new heuristic, the mode goodness ratio, to prioritise search sub-areas that present an higher probability of rediscovering the target. This work has a number of similarities with our approach. Both techniques aim to produce efficient flight manoeuvres for the UAV to maximise the probability of finding the target in the face of sensor limitations and environmental constraints. We use a spatial representation for the confusion level that is similar in spirit to the task difficulty map and we also exploit probabilistic reasoning to guide the planner to visit most promising sub-regions first. However, our approach is not devised specifically for path-planning, but it tackles the entire decision-making process that underpins the drone’s behaviour. As well as trajectories, we plan additional actions for the drones, such as performing search patterns, refilling depleted resources and avoiding localisation failures. In addition, we consider moving targets in wide spaces (hundreds of square kms), while in Lin and Goodrich (2014) the target is treated as stationary and assumed to be within a limited space.

### Orienteering problem

From a theoretical point of view, our formulation of SaT as a planning problem resembles the *Orienteering Problem with Time Windows* (OPTW) (Kantor and Rosenwein 1992). In a classical orienteering problem (OP), a set of vertices with associated rewards is given as well as a starting and an ending vertex. For all the vertices, the amount of time $t_{ij}$ needed to travel from vertex $v_i$ to vertex $v_j$ is known. Finally, a maximum time budget $T_{max}$ is established. The goal of the OP is to determine a path that visits a subset of the vertices in the available time $T_{max}$ in order to maximise the total collected reward. In the *time-window* variant of the OP, each vertex is associated with a time window and a visit to that vertex can only start during that window. The OPTW is a hard combinatorial problem because three types of decisions are involved in it: (i) allocation of vertices to be visited; (ii) sequencing of vertices to be visited; and (iii) scheduling of the visits to the chosen set of vertices. Considering our planning problem, the set of search patterns corresponds to the set of vertices of the OPTW problem, whereas the time slots in which the search patterns are active correspond to the OPTW time windows. As in the OPTW, we also want to maximise the total reward in the available amount of time (limited by the window of the latest possible search). However, in our case and differently from the OPTW, the planner can choose to visit each location more than once and, when a location is covered by more than one pattern, it also needs to decide which search pattern to use.

In the context of planning, the OP has been used to provide suitable heuristic advice on the goals and the goal order that should be considered by a planner that deals with over-subscription problems (Smith 2004).

## Conclusions and future work

In this paper, we have presented a hybrid approach to autonomous SaT that combines temporal planning with Monte Carlo simulation. This approach affords a number of benefits. Thanks to the use of sequences of search patterns, which individually can be of any size and orientation, we are able to inspect large and heterogeneous geographical areas. At the same time, since we use automated planning to generate these sequences of patterns, our approach is capable of building mission plans over long temporal horizons. Our method can be easily adapted to a variety of target behaviours and environments. This is because the generation of the initial set of candidate search patterns from which the planner chooses those to execute is based on the target PD, which is obtained by running MCS. Whereas the planning mechanism remains the same, several hypothesis can be incorporated in MCS to reflect different characteristics of the target motion model and the environment, which in turn will produce different initial distributions. Our method can also be easily extended to deal with multiple searchers and multiple targets. In these scenarios, the initial PD will represent probable locations of all the targets and the planning mechanism will take care of assigning different patterns to different searchers in order to maximise the probability of rediscovering the targets. For the sake of planning, search patterns performed by one search unit can be treated as obstacles by the other search units so as to avoid collisions between the searchers. Finally, another advantage of a plan-based approach to SaT is that the behaviour of the observer is predictable and well understood. A plan can be used as a common medium of exchange between the drones and the human observers, allowing safer interaction between the drones and other air traffic.

Our approach to SaT can be easily modified to support other robotics applications. In particular, it can be employed to tackle any problem under uncertainty in which Monte Carlo methods can help in formulating and weighting a set of initial hypotheses and planning can be leveraged to prove or disprove the validity of such hypotheses in an efficient and robust way. Several surveillance operations present this structure and can benefit from our method, for example intelligence gathering, in which observers are mobile and the targets correspond to interesting sites to be found, recorded and communicated, as well as hazard identification, in which observers are a mixture of mobile and fixed assets and targets are physical flaws in components being observed, or environmental readings that exceed safety levels. In addition, our approach is useful not only in the aerial domain, which is the focus of this paper, but also in different domains, such as for example underwater operations. Currently, in collaboration with SeeByte Inc. and in the context of the project “Planning Distributed Search Operations” funded by the Dstl ASUR (Autonomous Systems Underpinning Research) Programme,^5^ we are building on the SaT method illustrated here to devise a hybrid technique for underpinning the autonomous behaviour of multiple cooperative underwater vehicles involved in reconnaissance tasks.

In future work, we intend to construct a different formulation of the planning model, which will allow us to take into consideration the results of unsuccessful searches when choosing the sequence of patterns to execute. In particular, we would like to be able to change the rewards of the patterns based on information concerning which patterns have failed to rediscover the target in the past. In addition, we plan to implement a mixed-initiative framework in which a human operator can modify or recommend a plan or interfere with the execution of a plan. In such a framework, our approach could also be used to provide estimates of success for user generated plans. Finally, we would like to run more extensive experiments to compare our approach with other methods, such as SAROPS.

### Electronic supplementary material

Below is the link to the electronic supplementary material.

Supplementary material 1 (zip 45620 KB)

## Appendix

In what follows, we show the full specification of the UAV domain in PDDL2.2. The quadcopter domain together with examples of the problem specifications for both the UAV and the quadcopter can be retrieved in the Supplementary Material online, which also contains information about the map and the destinations used in the experiments reported in Sect. 8.

## Footnotes

1
Optic is open source and can be downloaded at: http://sourceforge.net/projects/tsgp/files/OPTIC/optic.tar.bz2/download.

2
http://wiki.ros.org/ardrone_autonomy.

3
http://wiki.ros.org/tum_ardrone.

4 Personal communication with Lawrence D. Stone.

5 Dstl Project Number: DSTLX-1000079686.
