# Supplementary material for: Combining temporal planning with probabilistic reasoning for autonomous surveillance missions
Source: Auton Robots. 2015 Dec 28;41(1):181–203. doi: 10.1007/s10514-015-9534-0 (PMC7175604; doi:10.1007/s10514-015-9534-0)
Supplement: Supplementary file 1 — Supplementary material 1 (zip 45620 KB) [file 10514_2015_9534_MOESM1_ESM.zip › SupplementaryMaterial/Quadcopter/ROSPackagesForParrotArDrone.pdf]

ROS Packages for Parrot Ar.Drone

We have used the following packages to implement our plan-based approach to SaT in the Parrot Ar.Drone:

Optic: http://sourceforge.net/projects/tsgp/files/OPTIC/optic.tar.bz2/download
This is the planner that we use to solve the planning problem.

ROSPlan: https://github.com/KCL-Planning/ROSPlan
It is a framework developed by King's College London for embedding a generic task planner in a ROS system. Together with the planner, ROSPlan is the crucial element of our architecture and provides tools to:

- automatically generate the initial state for the planner from the knowledge parsed from sensor data and stored in a knowledge base;
- automate calls to the planner, then post-process and validate the plan;
- handle the main dispatch loop, taking into account changing environment and action failure; and
- match planned actions to ROS action messages for lower level controllers.

ardrone_autonomy: ROS driver for Parrot AR-Drone quadrocopter. This driver is based on official AR-Drone SDK version 2.0 and supports both AR-Drone 1.0 and 2.0. This package is a fork of ardrone_brown driver (see below) and has been developed in the Autonomy Lab of Simon Fraser University. The driver has been tested on both ROS "Electric" and "Fuerte"1. The main features of the package are the following:

- Allow to access almost all sensor readings, debug values and status reports sent from the AR-Drone
- Information received from the drone will be published to the ardrone/navdata topic
- The navdata message also returns the special tags that are detected by the Drone's on-board vision processing system
- Allow to send commands to the drone (in order to fly the drone after takeoff, you can publish a message of type geometry_msgs::Twist to the cmd_vel topic)
- Other services (eg. Flight animations)

falkor_ardrone: ROS package that uses the "ardrone_autonomy" package to implement autonomous control functionality on an AR.Drone. The software is not documented at all, however this is what I think it does:

- The package should work on "fuerte"
- It launches the ardrone_driver from ardrone_autonomy to communicate with the AR.Drone
- Track target identified through a tag
- Based on OpenCV
- It implements an Haar feature-based cascade classifier for object detection

- The cascade detection is augmented with optical flow in case in a subsequent frame the tag gets missed by the detector
- PID controller using Navdata vx/vy provided by the ardrone_driver

tum_ardrone: ROS package which uses the "ardrone_autonomy" package to implement autonomous navigation in previously unknown and GPS-denied environments. The code works for both the AR.Drone 1.0 and 2.0 on ROS Fuerte. It has been developed by the Computer Vision Group of TUM Technische Universität München. The main features of the package are the following:

- Autonomous flying using videos from frontal camera for pose estimation and navigation
- Make the drone fly in a stable way to a given waypoint
- Get drone's exact pose in real time
- Make the drone hold a position
- Make the drone fly figures with absolute scale
- No obstacle avoidance or object recognition implemented yet
- 3 main nodes:
- drone_state-estimation: state estimation, including PTAM (Parallel Mapping and Tracking) and visualization
- drone_autopilot: drone controller, requires drone_state-estimation
- drone_gui: GUI for controlling the drone (with a joystick or KB) and for controlling the autopilot and the state-estimation node

tum_simulator: This ROS package contains the implementation of a Gazebo simulator for the Ardrone 1.0 and 2.0. It works on ROS Fuerte and on Gazebo 1.0.2 (ROS Fuerte plugin). It uses the "ardrone_autonomy" package. It has been developed by the Computer Vision Group of TUM Technische Universität München building on the package tu-darmstadt-ros-pkg, which contains ROS packages related to robotic research at the Technische Universität Darmstadt. The drone is controlled by a joystick or keyboard. Commands for observing the sensor data coming from the drone are also provided.

brown-ros-pkg: collection of software resources for supporting quick development of robotic application on ROS developed at Brown University. The packages work on ROS Electric only. Relevant packages:

- - **ardrone_brown:** a ROS driver for the AR-Drone 1.0. The new package ardrone_autonomy has been developed on the basis of this driver.
- - **ar_recog:** ROS vision node that allows for recognition of ARTags and their location/transformation in image-space. This package is based on ARToolKit, a software library for building Augmented Reality (AR) applications.
- - **nolan:** PID controller that, when integrated with ar_recog, implements tag following.
