# Supplementary figures and images for: Combining temporal planning with probabilistic reasoning for autonomous surveillance missions (part 2 of 2)
Source: Auton Robots. 2015 Dec 28;41(1):181–203. doi: 10.1007/s10514-015-9534-0 (PMC7175604; doi:10.1007/s10514-015-9534-0)

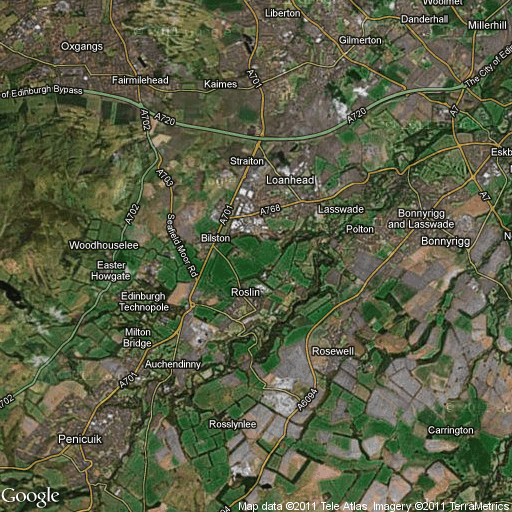

Supplement: Supplementary file 1 — Supplementary material 1 (zip 45620 KB) [file 10514_2015_9534_MOESM1_ESM.zip › SupplementaryMaterial/UAV/maptiles/atile_8_1.png]

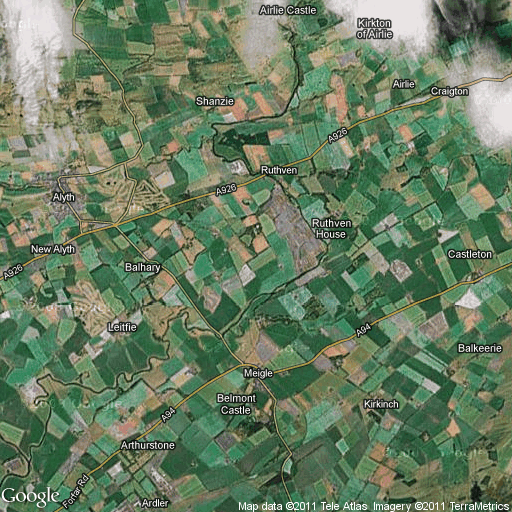

Supplement: Supplementary file 1 — Supplementary material 1 (zip 45620 KB) [file 10514_2015_9534_MOESM1_ESM.zip › SupplementaryMaterial/UAV/maptiles/atile_8_10.png]

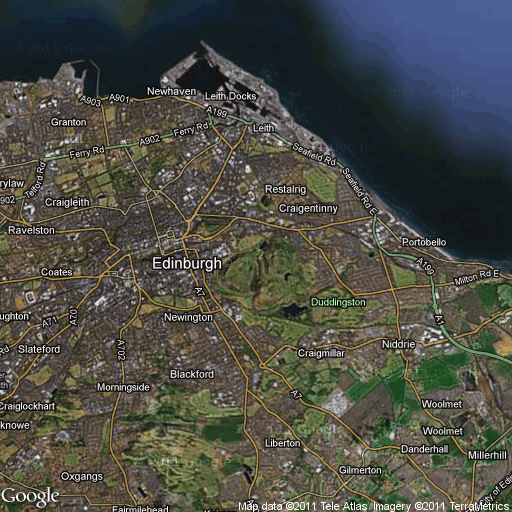

Supplement: Supplementary file 1 — Supplementary material 1 (zip 45620 KB) [file 10514_2015_9534_MOESM1_ESM.zip › SupplementaryMaterial/UAV/maptiles/atile_8_2.png]

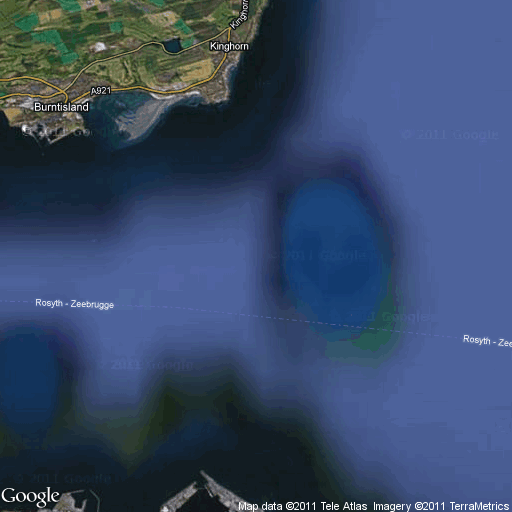

Supplement: Supplementary file 1 — Supplementary material 1 (zip 45620 KB) [file 10514_2015_9534_MOESM1_ESM.zip › SupplementaryMaterial/UAV/maptiles/atile_8_3.png]

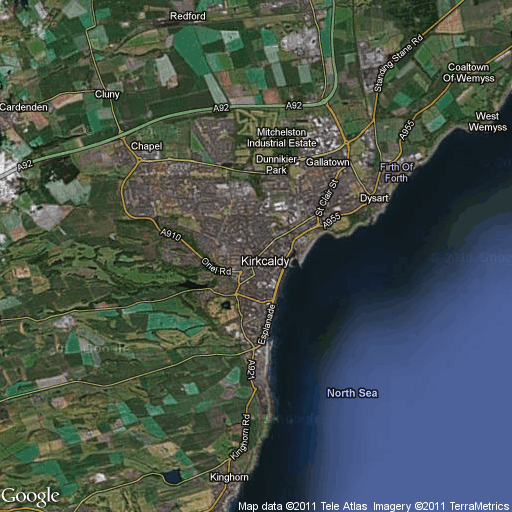

Supplement: Supplementary file 1 — Supplementary material 1 (zip 45620 KB) [file 10514_2015_9534_MOESM1_ESM.zip › SupplementaryMaterial/UAV/maptiles/atile_8_4.png]

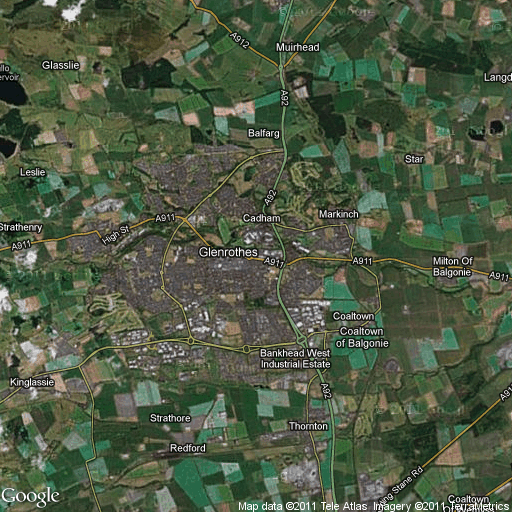

Supplement: Supplementary file 1 — Supplementary material 1 (zip 45620 KB) [file 10514_2015_9534_MOESM1_ESM.zip › SupplementaryMaterial/UAV/maptiles/atile_8_5.png]

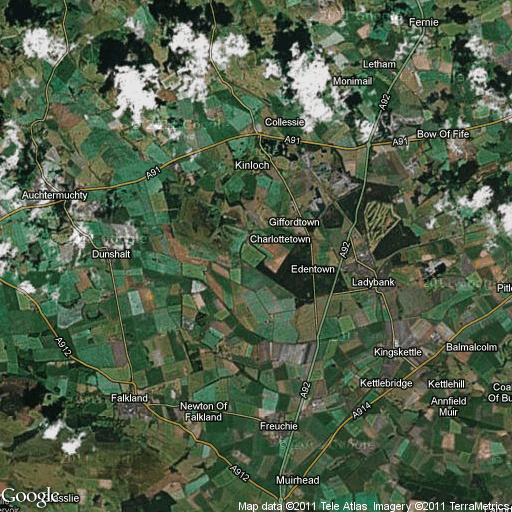

Supplement: Supplementary file 1 — Supplementary material 1 (zip 45620 KB) [file 10514_2015_9534_MOESM1_ESM.zip › SupplementaryMaterial/UAV/maptiles/atile_8_6.png]

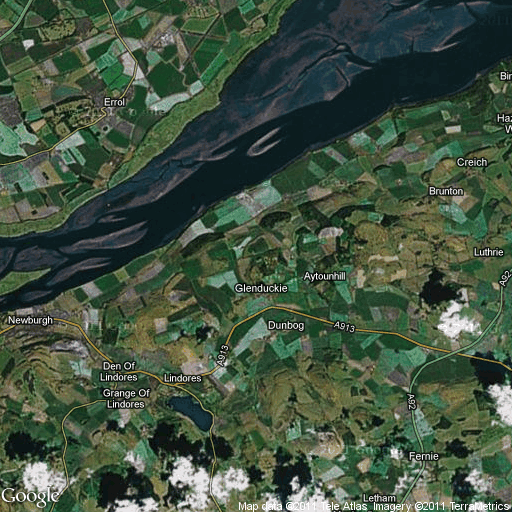

Supplement: Supplementary file 1 — Supplementary material 1 (zip 45620 KB) [file 10514_2015_9534_MOESM1_ESM.zip › SupplementaryMaterial/UAV/maptiles/atile_8_7.png]

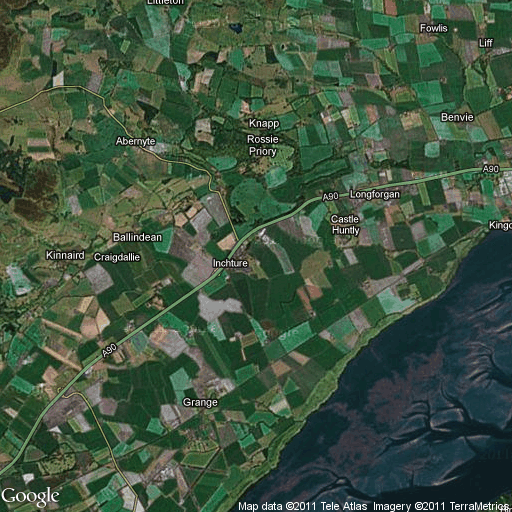

Supplement: Supplementary file 1 — Supplementary material 1 (zip 45620 KB) [file 10514_2015_9534_MOESM1_ESM.zip › SupplementaryMaterial/UAV/maptiles/atile_8_8.png]

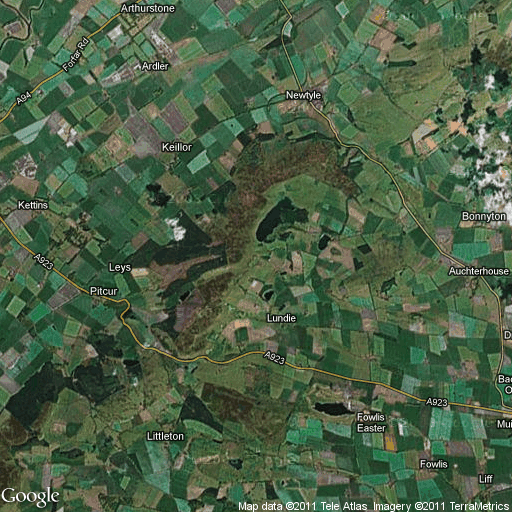

Supplement: Supplementary file 1 — Supplementary material 1 (zip 45620 KB) [file 10514_2015_9534_MOESM1_ESM.zip › SupplementaryMaterial/UAV/maptiles/atile_8_9.png]

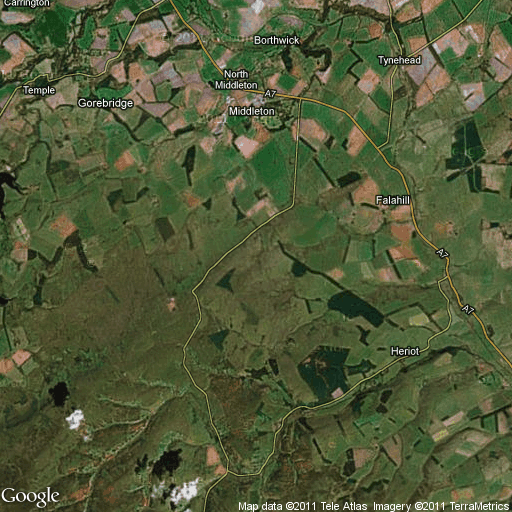

Supplement: Supplementary file 1 — Supplementary material 1 (zip 45620 KB) [file 10514_2015_9534_MOESM1_ESM.zip › SupplementaryMaterial/UAV/maptiles/atile_9_0.png]

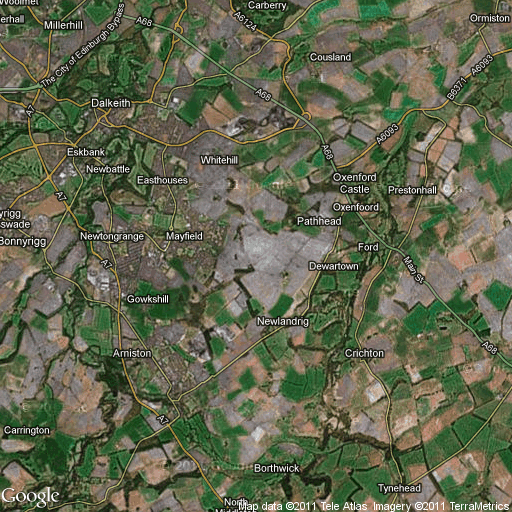

Supplement: Supplementary file 1 — Supplementary material 1 (zip 45620 KB) [file 10514_2015_9534_MOESM1_ESM.zip › SupplementaryMaterial/UAV/maptiles/atile_9_1.png]

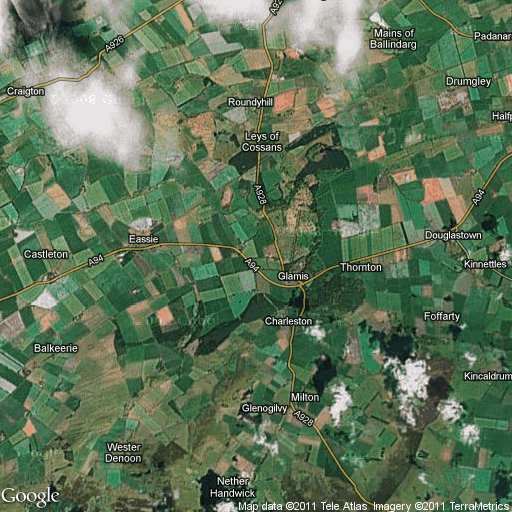

Supplement: Supplementary file 1 — Supplementary material 1 (zip 45620 KB) [file 10514_2015_9534_MOESM1_ESM.zip › SupplementaryMaterial/UAV/maptiles/atile_9_10.png]

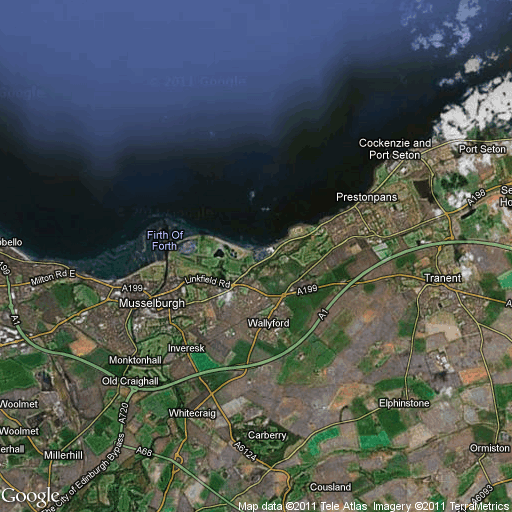

Supplement: Supplementary file 1 — Supplementary material 1 (zip 45620 KB) [file 10514_2015_9534_MOESM1_ESM.zip › SupplementaryMaterial/UAV/maptiles/atile_9_2.png]

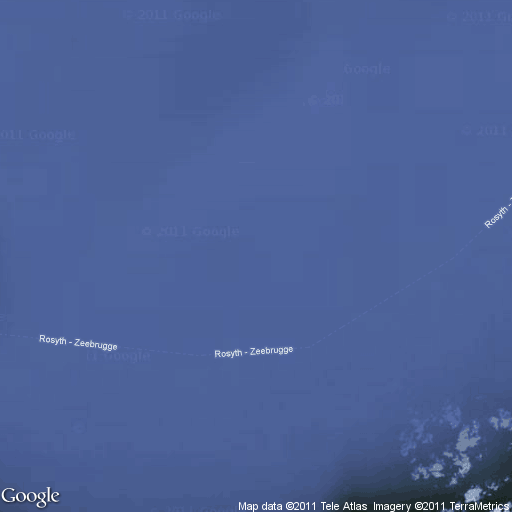

Supplement: Supplementary file 1 — Supplementary material 1 (zip 45620 KB) [file 10514_2015_9534_MOESM1_ESM.zip › SupplementaryMaterial/UAV/maptiles/atile_9_3.png]

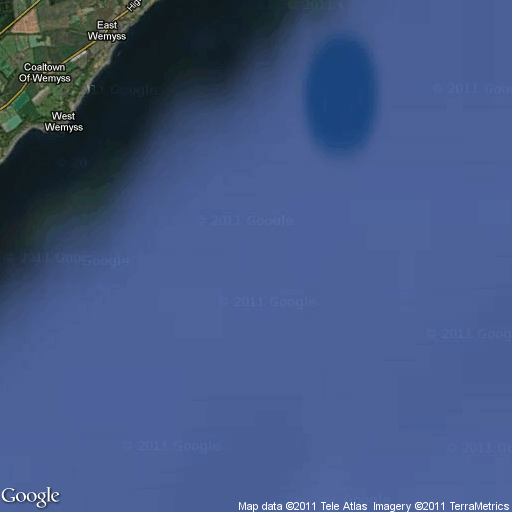

Supplement: Supplementary file 1 — Supplementary material 1 (zip 45620 KB) [file 10514_2015_9534_MOESM1_ESM.zip › SupplementaryMaterial/UAV/maptiles/atile_9_4.png]

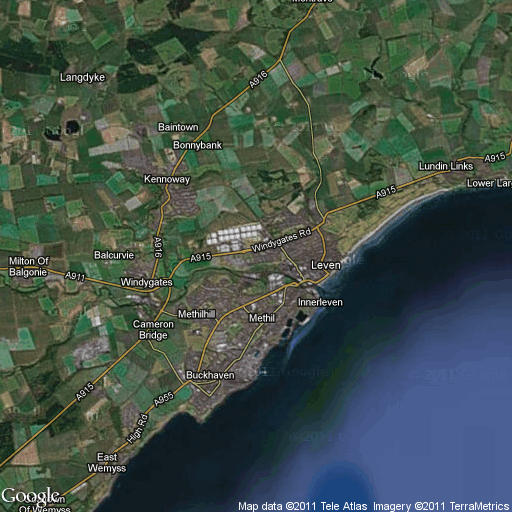

Supplement: Supplementary file 1 — Supplementary material 1 (zip 45620 KB) [file 10514_2015_9534_MOESM1_ESM.zip › SupplementaryMaterial/UAV/maptiles/atile_9_5.png]

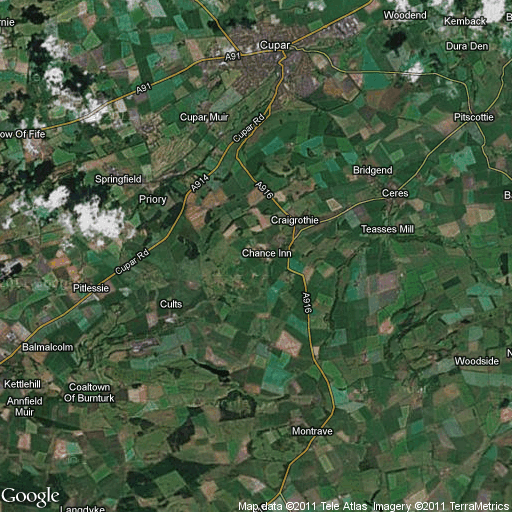

Supplement: Supplementary file 1 — Supplementary material 1 (zip 45620 KB) [file 10514_2015_9534_MOESM1_ESM.zip › SupplementaryMaterial/UAV/maptiles/atile_9_6.png]

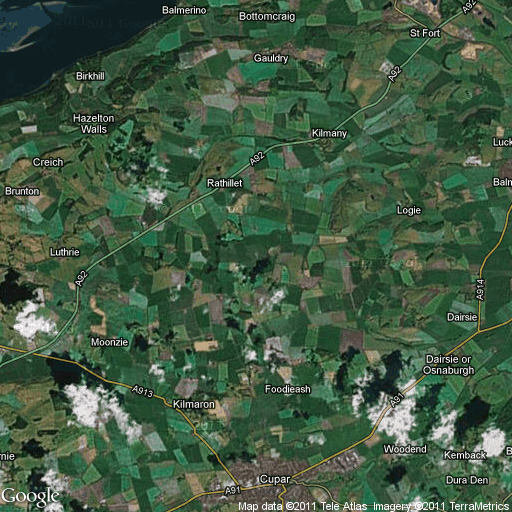

Supplement: Supplementary file 1 — Supplementary material 1 (zip 45620 KB) [file 10514_2015_9534_MOESM1_ESM.zip › SupplementaryMaterial/UAV/maptiles/atile_9_7.png]

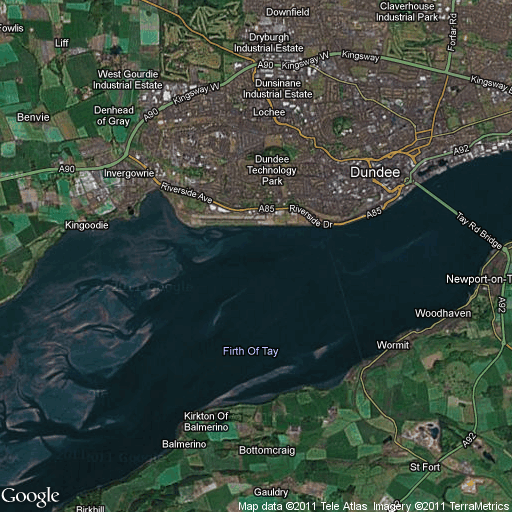

Supplement: Supplementary file 1 — Supplementary material 1 (zip 45620 KB) [file 10514_2015_9534_MOESM1_ESM.zip › SupplementaryMaterial/UAV/maptiles/atile_9_8.png]

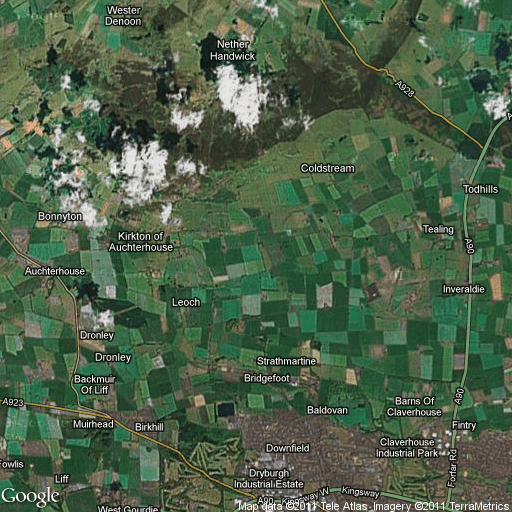

Supplement: Supplementary file 1 — Supplementary material 1 (zip 45620 KB) [file 10514_2015_9534_MOESM1_ESM.zip › SupplementaryMaterial/UAV/maptiles/atile_9_9.png]
