# Supplementary figures and images for: Combining temporal planning with probabilistic reasoning for autonomous surveillance missions (part 1 of 2)
Source: Auton Robots. 2015 Dec 28;41(1):181–203. doi: 10.1007/s10514-015-9534-0 (PMC7175604; doi:10.1007/s10514-015-9534-0)

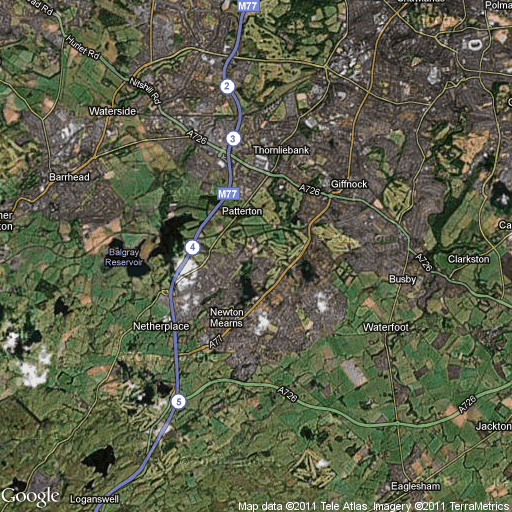

Supplement: Supplementary file 1 — Supplementary material 1 (zip 45620 KB) [file 10514_2015_9534_MOESM1_ESM.zip › SupplementaryMaterial/UAV/maptiles/atile_0_0.png]

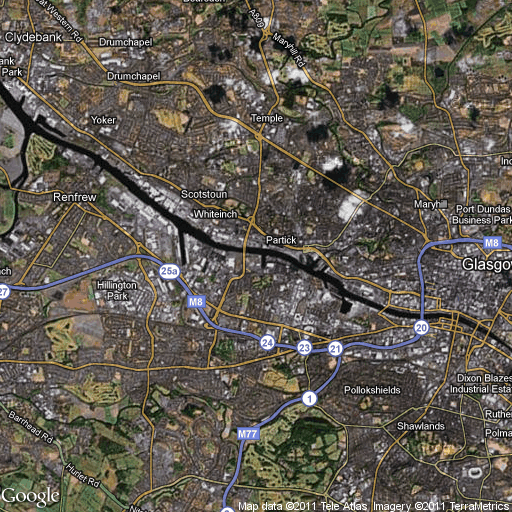

Supplement: Supplementary file 1 — Supplementary material 1 (zip 45620 KB) [file 10514_2015_9534_MOESM1_ESM.zip › SupplementaryMaterial/UAV/maptiles/atile_0_1.png]

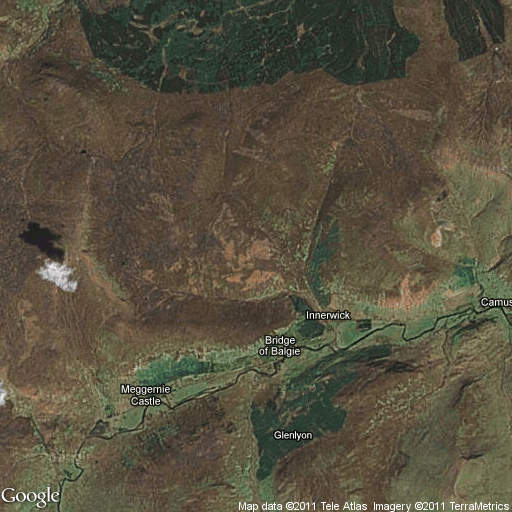

Supplement: Supplementary file 1 — Supplementary material 1 (zip 45620 KB) [file 10514_2015_9534_MOESM1_ESM.zip › SupplementaryMaterial/UAV/maptiles/atile_0_10.png]

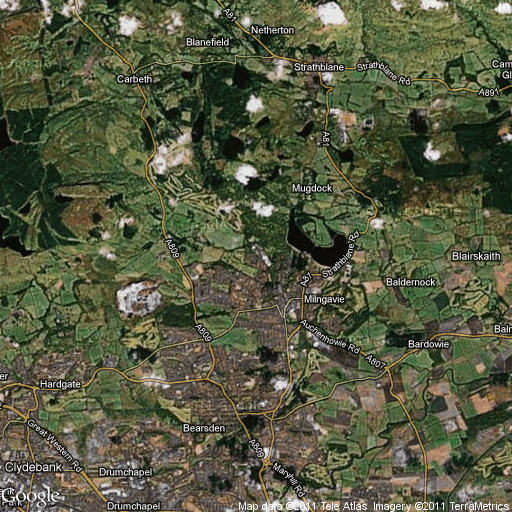

Supplement: Supplementary file 1 — Supplementary material 1 (zip 45620 KB) [file 10514_2015_9534_MOESM1_ESM.zip › SupplementaryMaterial/UAV/maptiles/atile_0_2.png]

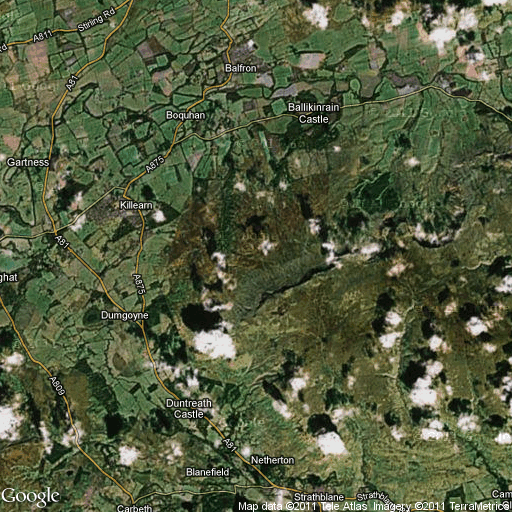

Supplement: Supplementary file 1 — Supplementary material 1 (zip 45620 KB) [file 10514_2015_9534_MOESM1_ESM.zip › SupplementaryMaterial/UAV/maptiles/atile_0_3.png]

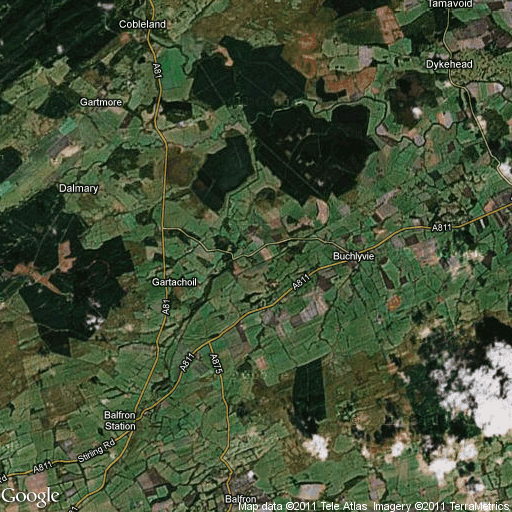

Supplement: Supplementary file 1 — Supplementary material 1 (zip 45620 KB) [file 10514_2015_9534_MOESM1_ESM.zip › SupplementaryMaterial/UAV/maptiles/atile_0_4.png]

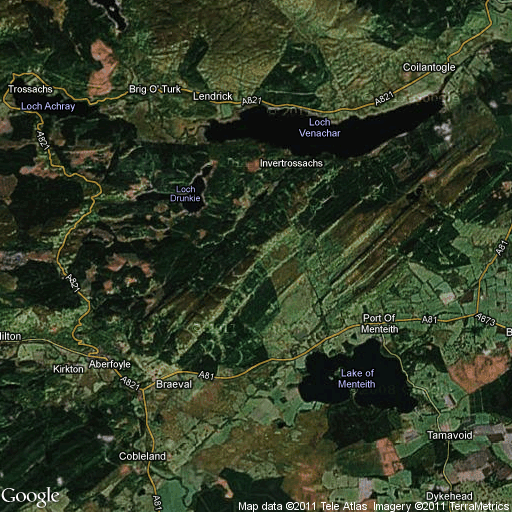

Supplement: Supplementary file 1 — Supplementary material 1 (zip 45620 KB) [file 10514_2015_9534_MOESM1_ESM.zip › SupplementaryMaterial/UAV/maptiles/atile_0_5.png]

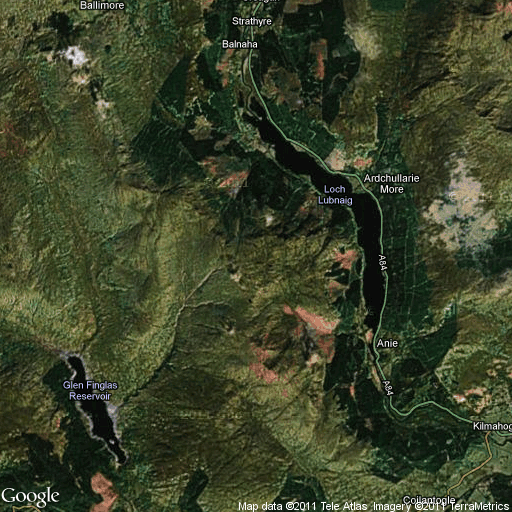

Supplement: Supplementary file 1 — Supplementary material 1 (zip 45620 KB) [file 10514_2015_9534_MOESM1_ESM.zip › SupplementaryMaterial/UAV/maptiles/atile_0_6.png]

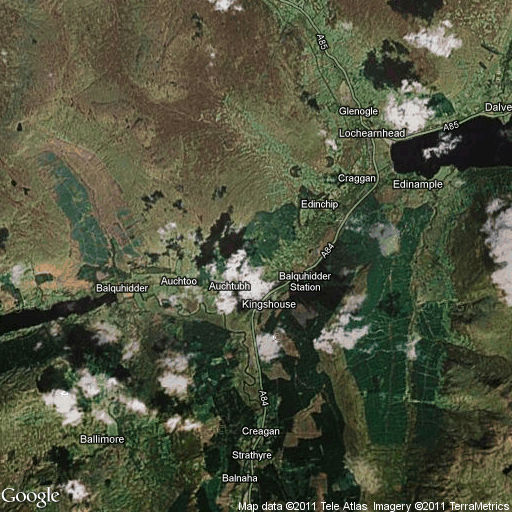

Supplement: Supplementary file 1 — Supplementary material 1 (zip 45620 KB) [file 10514_2015_9534_MOESM1_ESM.zip › SupplementaryMaterial/UAV/maptiles/atile_0_7.png]

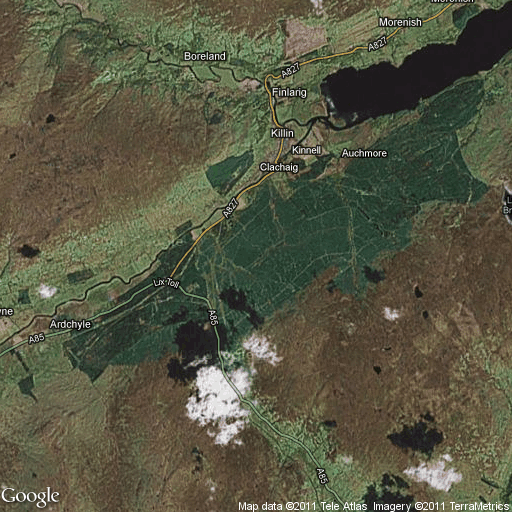

Supplement: Supplementary file 1 — Supplementary material 1 (zip 45620 KB) [file 10514_2015_9534_MOESM1_ESM.zip › SupplementaryMaterial/UAV/maptiles/atile_0_8.png]

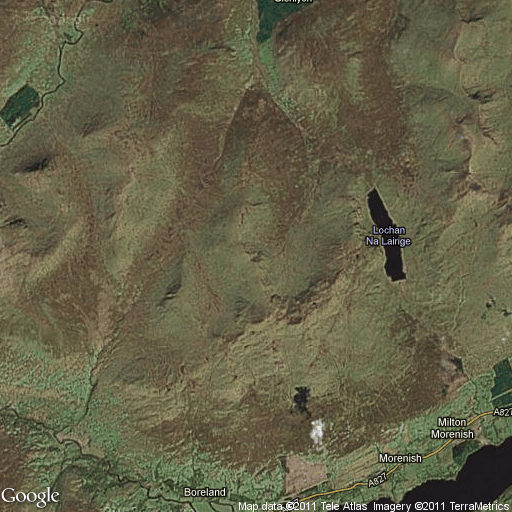

Supplement: Supplementary file 1 — Supplementary material 1 (zip 45620 KB) [file 10514_2015_9534_MOESM1_ESM.zip › SupplementaryMaterial/UAV/maptiles/atile_0_9.png]

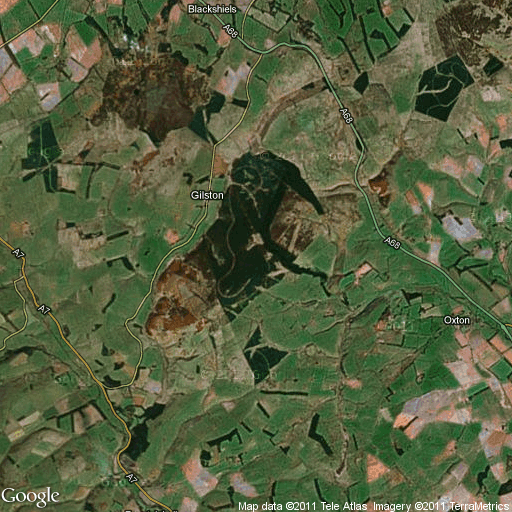

Supplement: Supplementary file 1 — Supplementary material 1 (zip 45620 KB) [file 10514_2015_9534_MOESM1_ESM.zip › SupplementaryMaterial/UAV/maptiles/atile_10_0.png]

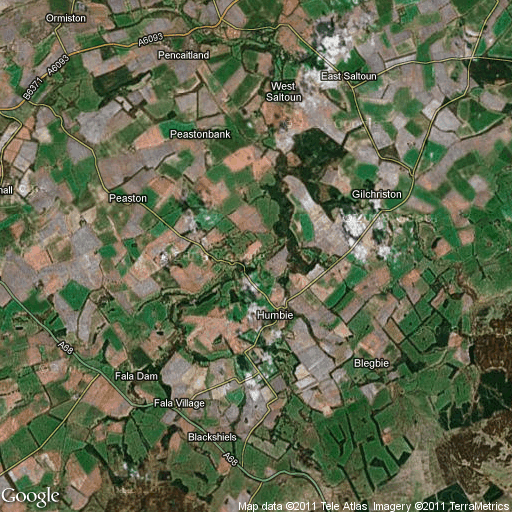

Supplement: Supplementary file 1 — Supplementary material 1 (zip 45620 KB) [file 10514_2015_9534_MOESM1_ESM.zip › SupplementaryMaterial/UAV/maptiles/atile_10_1.png]

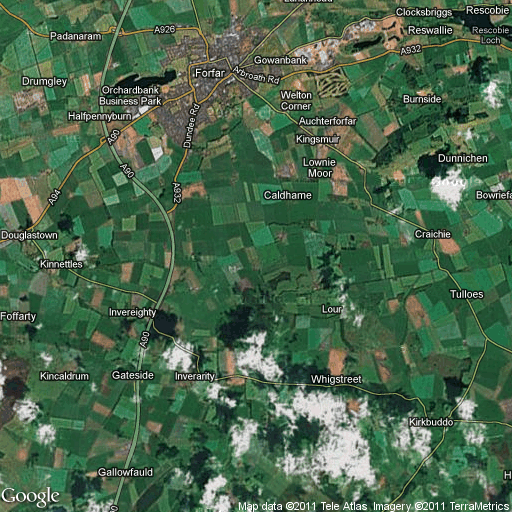

Supplement: Supplementary file 1 — Supplementary material 1 (zip 45620 KB) [file 10514_2015_9534_MOESM1_ESM.zip › SupplementaryMaterial/UAV/maptiles/atile_10_10.png]

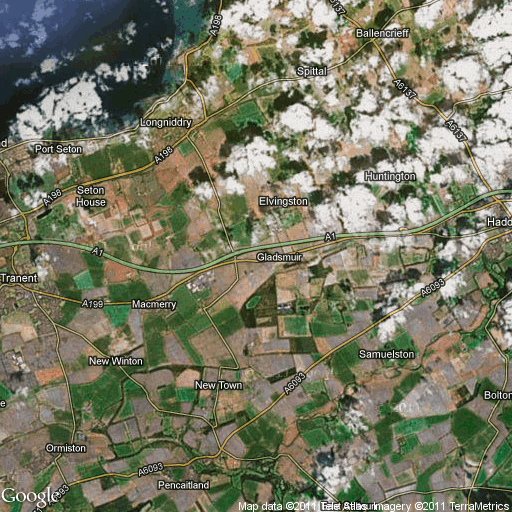

Supplement: Supplementary file 1 — Supplementary material 1 (zip 45620 KB) [file 10514_2015_9534_MOESM1_ESM.zip › SupplementaryMaterial/UAV/maptiles/atile_10_2.png]

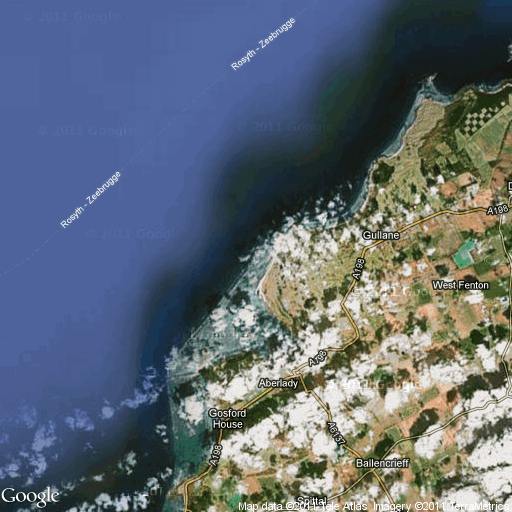

Supplement: Supplementary file 1 — Supplementary material 1 (zip 45620 KB) [file 10514_2015_9534_MOESM1_ESM.zip › SupplementaryMaterial/UAV/maptiles/atile_10_3.png]

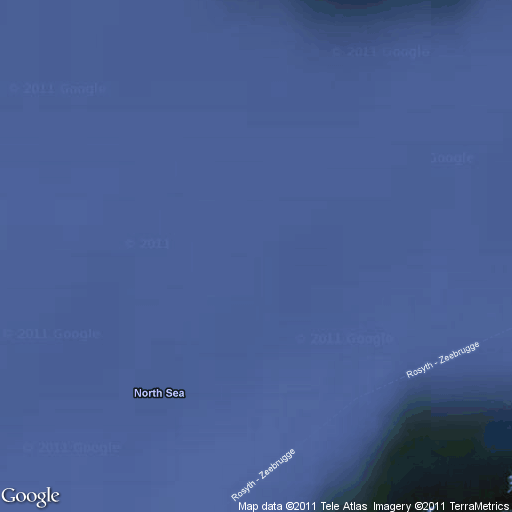

Supplement: Supplementary file 1 — Supplementary material 1 (zip 45620 KB) [file 10514_2015_9534_MOESM1_ESM.zip › SupplementaryMaterial/UAV/maptiles/atile_10_4.png]

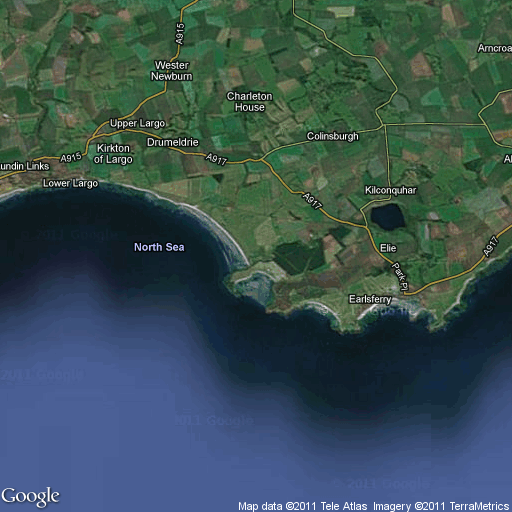

Supplement: Supplementary file 1 — Supplementary material 1 (zip 45620 KB) [file 10514_2015_9534_MOESM1_ESM.zip › SupplementaryMaterial/UAV/maptiles/atile_10_5.png]

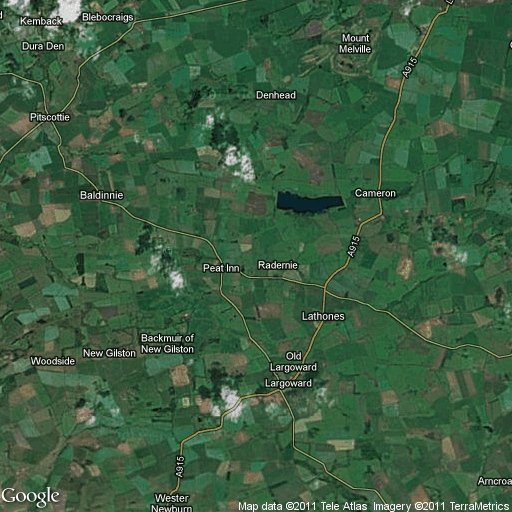

Supplement: Supplementary file 1 — Supplementary material 1 (zip 45620 KB) [file 10514_2015_9534_MOESM1_ESM.zip › SupplementaryMaterial/UAV/maptiles/atile_10_6.png]

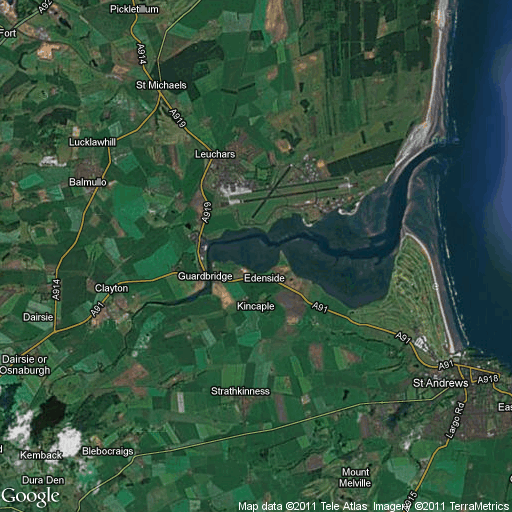

Supplement: Supplementary file 1 — Supplementary material 1 (zip 45620 KB) [file 10514_2015_9534_MOESM1_ESM.zip › SupplementaryMaterial/UAV/maptiles/atile_10_7.png]

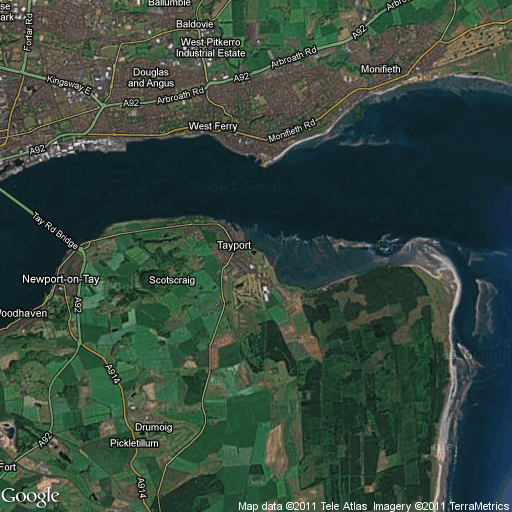

Supplement: Supplementary file 1 — Supplementary material 1 (zip 45620 KB) [file 10514_2015_9534_MOESM1_ESM.zip › SupplementaryMaterial/UAV/maptiles/atile_10_8.png]

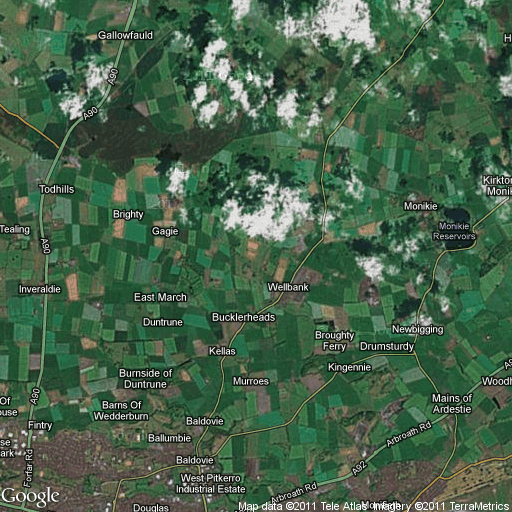

Supplement: Supplementary file 1 — Supplementary material 1 (zip 45620 KB) [file 10514_2015_9534_MOESM1_ESM.zip › SupplementaryMaterial/UAV/maptiles/atile_10_9.png]

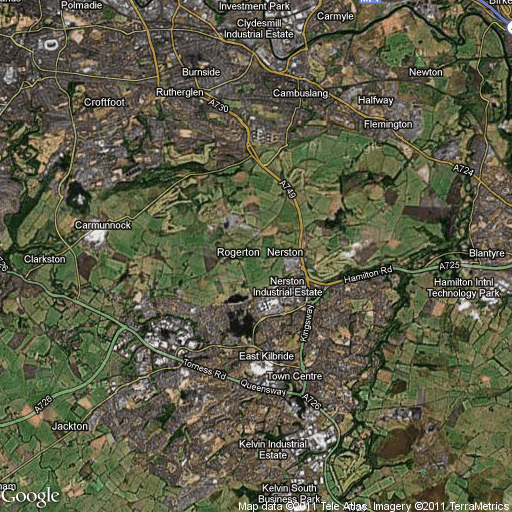

Supplement: Supplementary file 1 — Supplementary material 1 (zip 45620 KB) [file 10514_2015_9534_MOESM1_ESM.zip › SupplementaryMaterial/UAV/maptiles/atile_1_0.png]

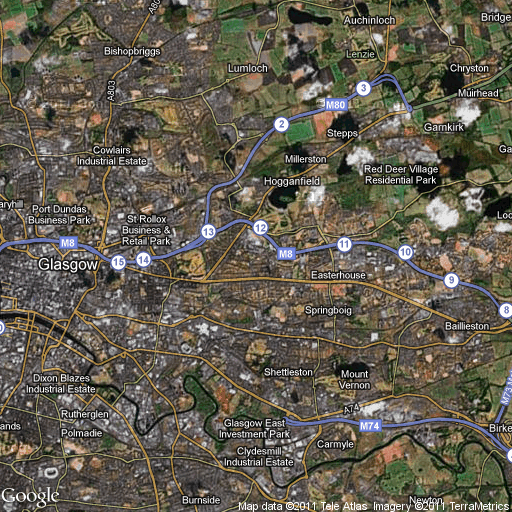

Supplement: Supplementary file 1 — Supplementary material 1 (zip 45620 KB) [file 10514_2015_9534_MOESM1_ESM.zip › SupplementaryMaterial/UAV/maptiles/atile_1_1.png]

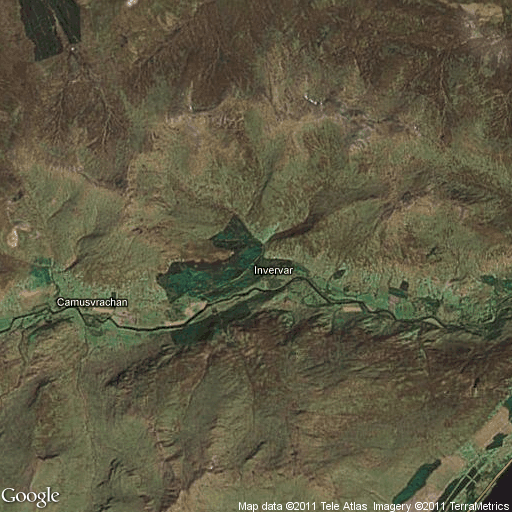

Supplement: Supplementary file 1 — Supplementary material 1 (zip 45620 KB) [file 10514_2015_9534_MOESM1_ESM.zip › SupplementaryMaterial/UAV/maptiles/atile_1_10.png]

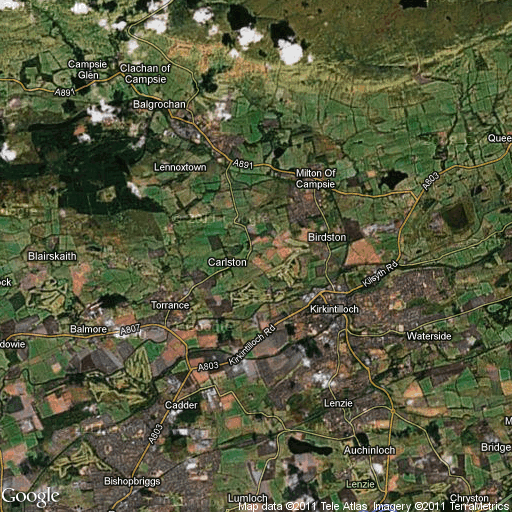

Supplement: Supplementary file 1 — Supplementary material 1 (zip 45620 KB) [file 10514_2015_9534_MOESM1_ESM.zip › SupplementaryMaterial/UAV/maptiles/atile_1_2.png]

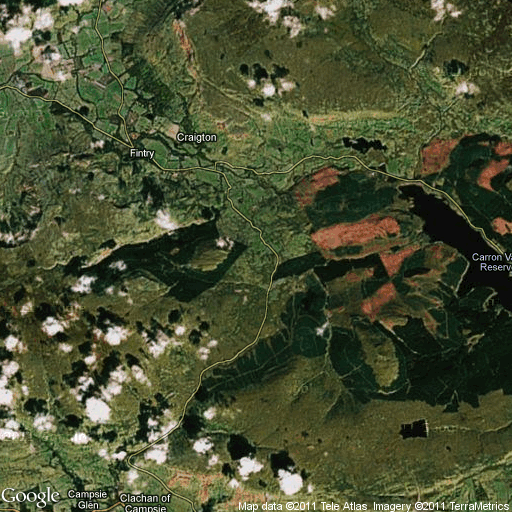

Supplement: Supplementary file 1 — Supplementary material 1 (zip 45620 KB) [file 10514_2015_9534_MOESM1_ESM.zip › SupplementaryMaterial/UAV/maptiles/atile_1_3.png]

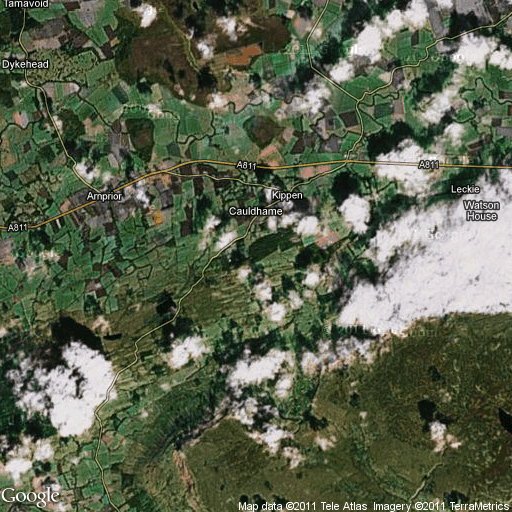

Supplement: Supplementary file 1 — Supplementary material 1 (zip 45620 KB) [file 10514_2015_9534_MOESM1_ESM.zip › SupplementaryMaterial/UAV/maptiles/atile_1_4.png]

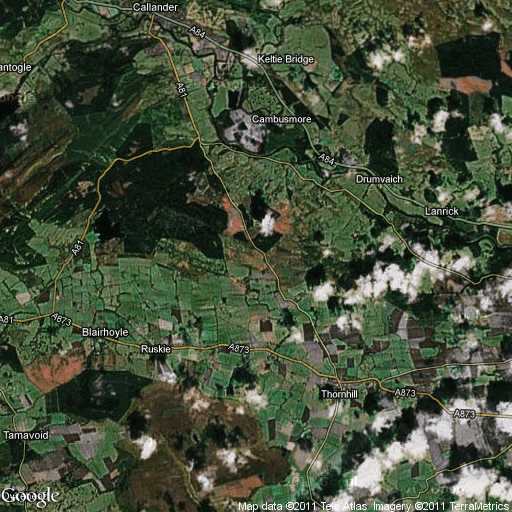

Supplement: Supplementary file 1 — Supplementary material 1 (zip 45620 KB) [file 10514_2015_9534_MOESM1_ESM.zip › SupplementaryMaterial/UAV/maptiles/atile_1_5.png]

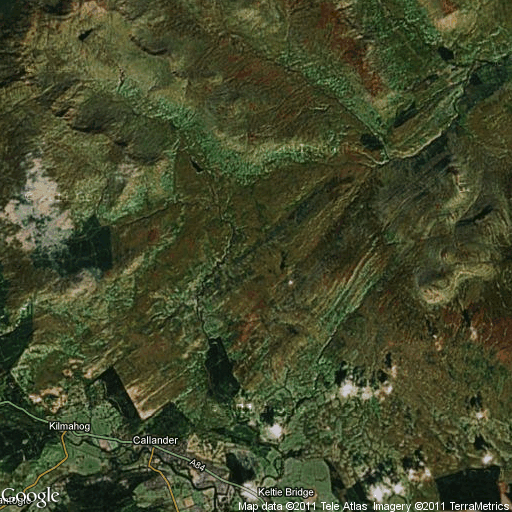

Supplement: Supplementary file 1 — Supplementary material 1 (zip 45620 KB) [file 10514_2015_9534_MOESM1_ESM.zip › SupplementaryMaterial/UAV/maptiles/atile_1_6.png]

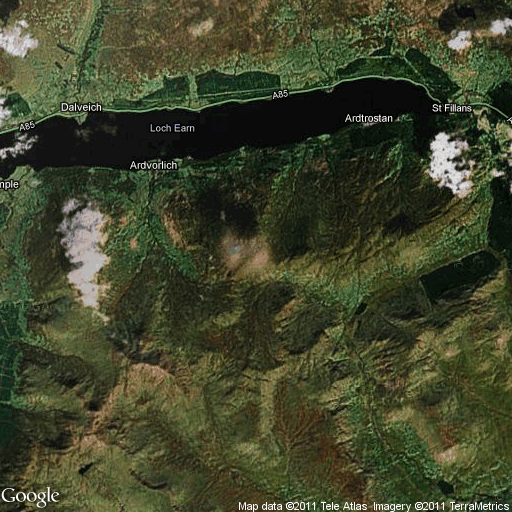

Supplement: Supplementary file 1 — Supplementary material 1 (zip 45620 KB) [file 10514_2015_9534_MOESM1_ESM.zip › SupplementaryMaterial/UAV/maptiles/atile_1_7.png]

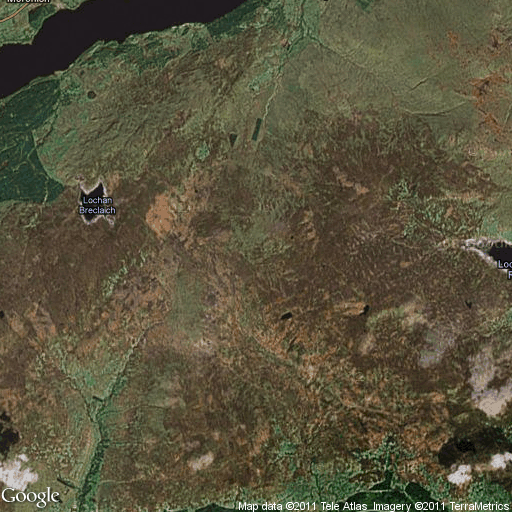

Supplement: Supplementary file 1 — Supplementary material 1 (zip 45620 KB) [file 10514_2015_9534_MOESM1_ESM.zip › SupplementaryMaterial/UAV/maptiles/atile_1_8.png]

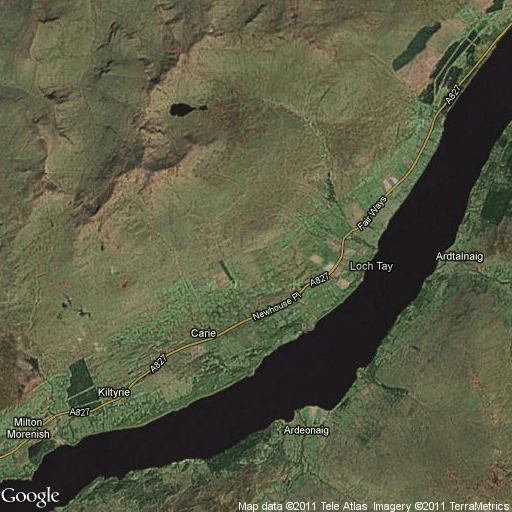

Supplement: Supplementary file 1 — Supplementary material 1 (zip 45620 KB) [file 10514_2015_9534_MOESM1_ESM.zip › SupplementaryMaterial/UAV/maptiles/atile_1_9.png]

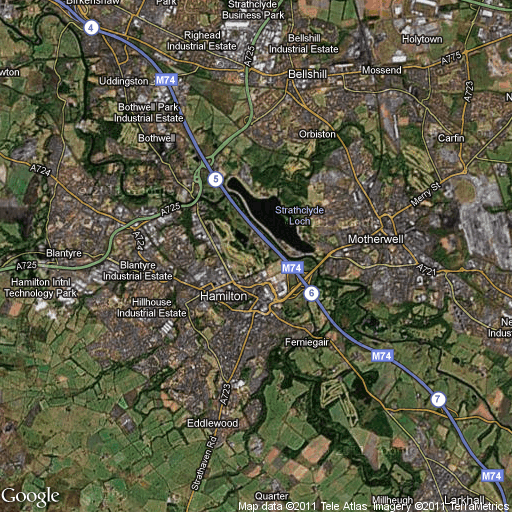

Supplement: Supplementary file 1 — Supplementary material 1 (zip 45620 KB) [file 10514_2015_9534_MOESM1_ESM.zip › SupplementaryMaterial/UAV/maptiles/atile_2_0.png]

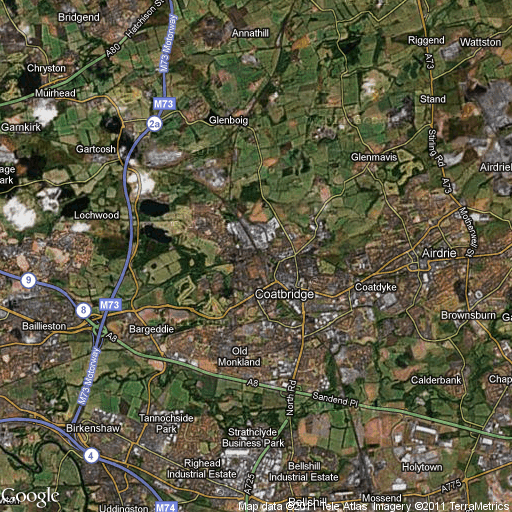

Supplement: Supplementary file 1 — Supplementary material 1 (zip 45620 KB) [file 10514_2015_9534_MOESM1_ESM.zip › SupplementaryMaterial/UAV/maptiles/atile_2_1.png]

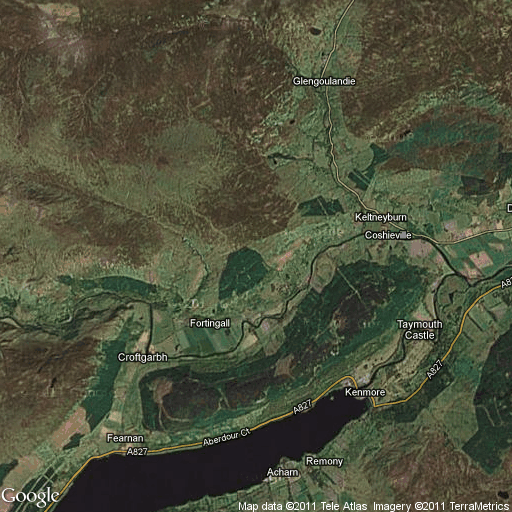

Supplement: Supplementary file 1 — Supplementary material 1 (zip 45620 KB) [file 10514_2015_9534_MOESM1_ESM.zip › SupplementaryMaterial/UAV/maptiles/atile_2_10.png]

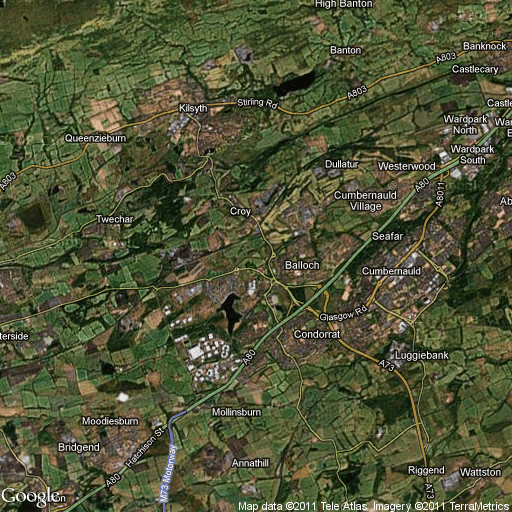

Supplement: Supplementary file 1 — Supplementary material 1 (zip 45620 KB) [file 10514_2015_9534_MOESM1_ESM.zip › SupplementaryMaterial/UAV/maptiles/atile_2_2.png]

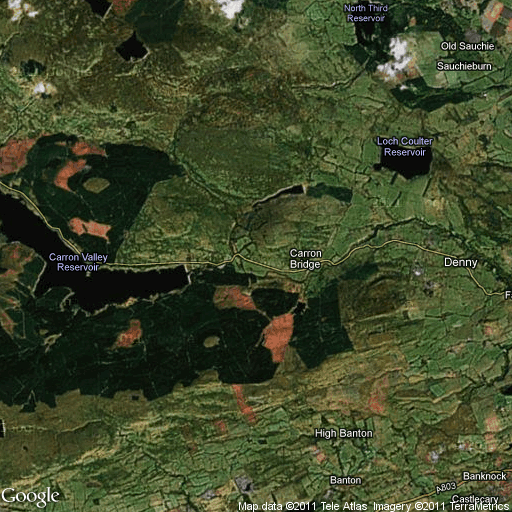

Supplement: Supplementary file 1 — Supplementary material 1 (zip 45620 KB) [file 10514_2015_9534_MOESM1_ESM.zip › SupplementaryMaterial/UAV/maptiles/atile_2_3.png]

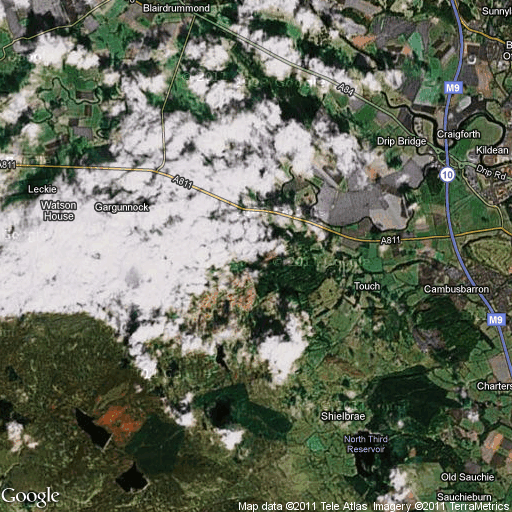

Supplement: Supplementary file 1 — Supplementary material 1 (zip 45620 KB) [file 10514_2015_9534_MOESM1_ESM.zip › SupplementaryMaterial/UAV/maptiles/atile_2_4.png]

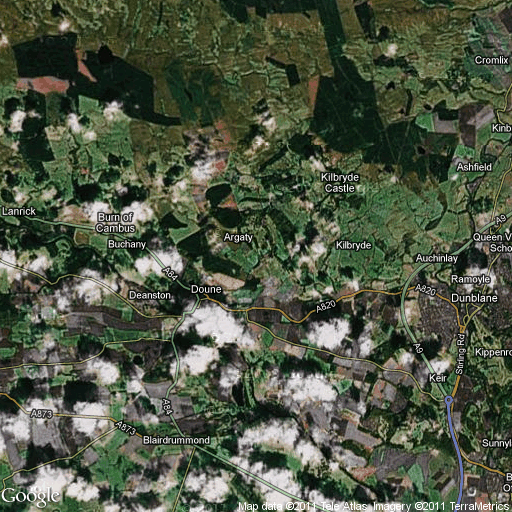

Supplement: Supplementary file 1 — Supplementary material 1 (zip 45620 KB) [file 10514_2015_9534_MOESM1_ESM.zip › SupplementaryMaterial/UAV/maptiles/atile_2_5.png]

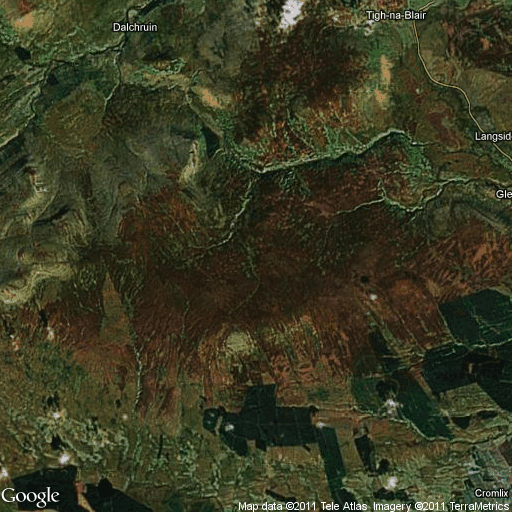

Supplement: Supplementary file 1 — Supplementary material 1 (zip 45620 KB) [file 10514_2015_9534_MOESM1_ESM.zip › SupplementaryMaterial/UAV/maptiles/atile_2_6.png]

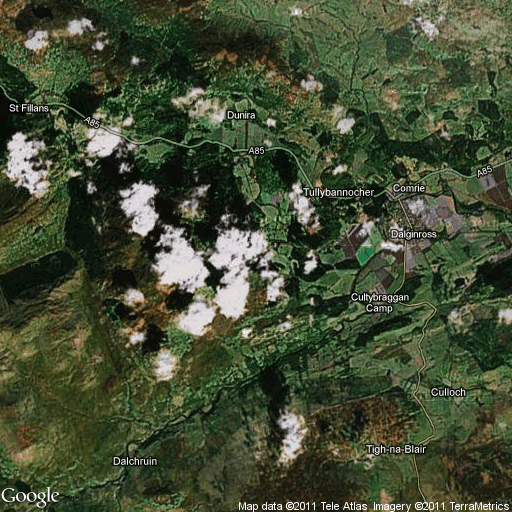

Supplement: Supplementary file 1 — Supplementary material 1 (zip 45620 KB) [file 10514_2015_9534_MOESM1_ESM.zip › SupplementaryMaterial/UAV/maptiles/atile_2_7.png]

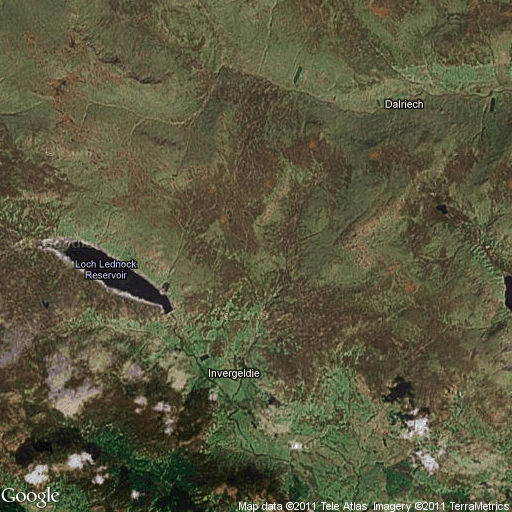

Supplement: Supplementary file 1 — Supplementary material 1 (zip 45620 KB) [file 10514_2015_9534_MOESM1_ESM.zip › SupplementaryMaterial/UAV/maptiles/atile_2_8.png]

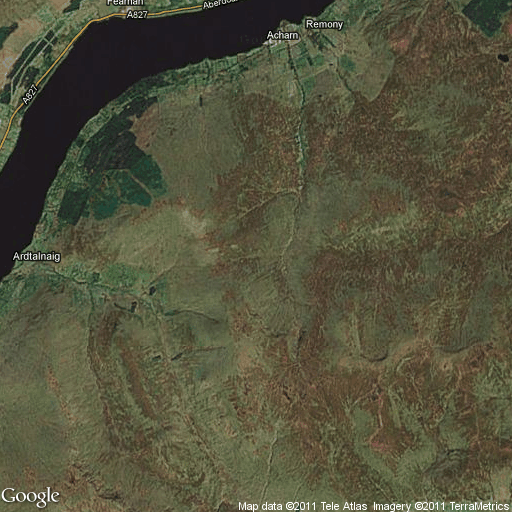

Supplement: Supplementary file 1 — Supplementary material 1 (zip 45620 KB) [file 10514_2015_9534_MOESM1_ESM.zip › SupplementaryMaterial/UAV/maptiles/atile_2_9.png]

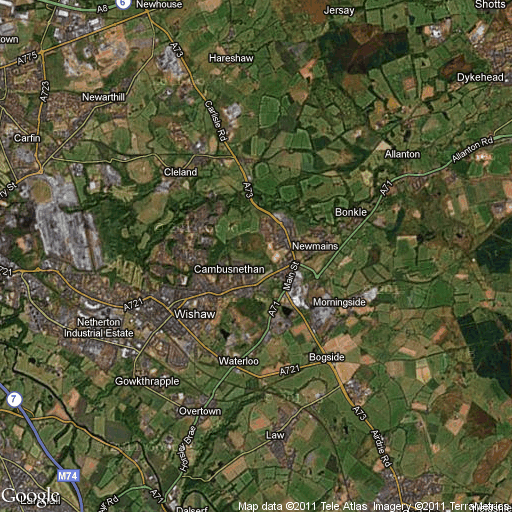

Supplement: Supplementary file 1 — Supplementary material 1 (zip 45620 KB) [file 10514_2015_9534_MOESM1_ESM.zip › SupplementaryMaterial/UAV/maptiles/atile_3_0.png]

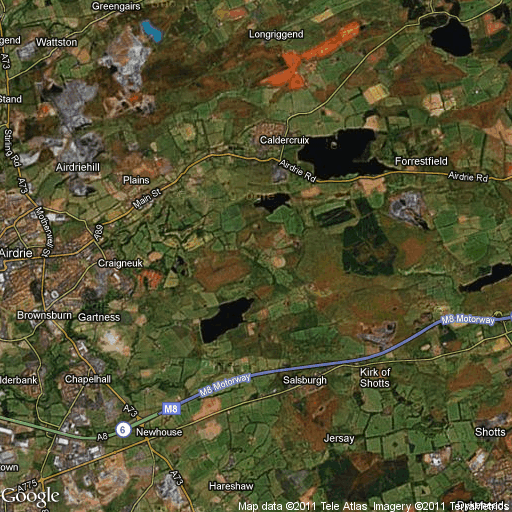

Supplement: Supplementary file 1 — Supplementary material 1 (zip 45620 KB) [file 10514_2015_9534_MOESM1_ESM.zip › SupplementaryMaterial/UAV/maptiles/atile_3_1.png]

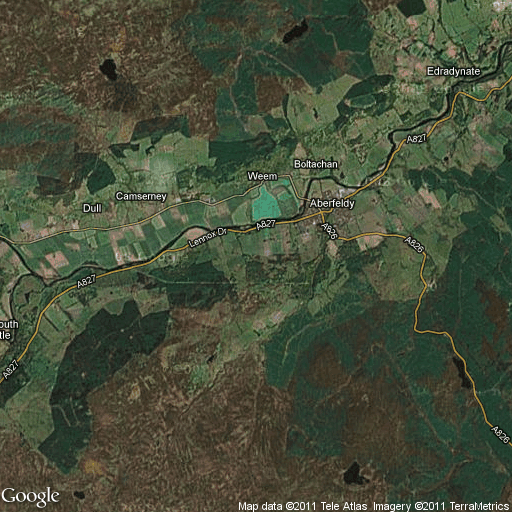

Supplement: Supplementary file 1 — Supplementary material 1 (zip 45620 KB) [file 10514_2015_9534_MOESM1_ESM.zip › SupplementaryMaterial/UAV/maptiles/atile_3_10.png]

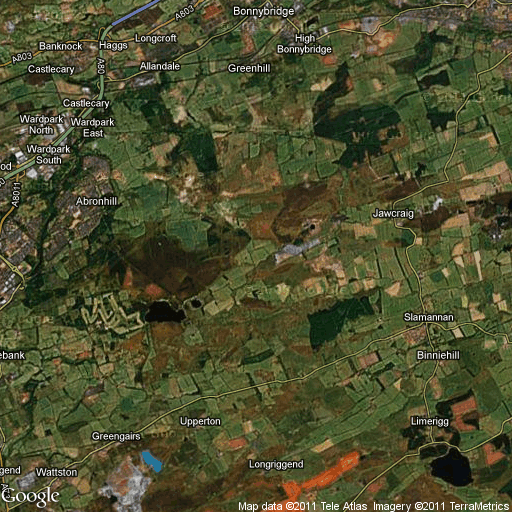

Supplement: Supplementary file 1 — Supplementary material 1 (zip 45620 KB) [file 10514_2015_9534_MOESM1_ESM.zip › SupplementaryMaterial/UAV/maptiles/atile_3_2.png]

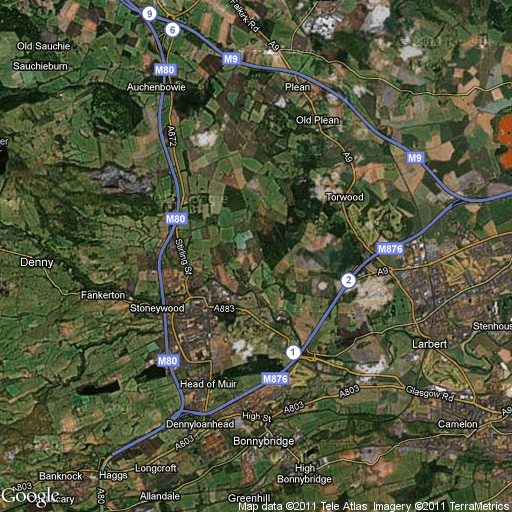

Supplement: Supplementary file 1 — Supplementary material 1 (zip 45620 KB) [file 10514_2015_9534_MOESM1_ESM.zip › SupplementaryMaterial/UAV/maptiles/atile_3_3.png]

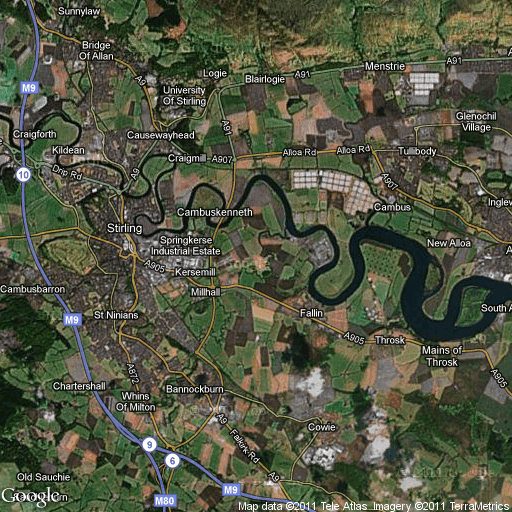

Supplement: Supplementary file 1 — Supplementary material 1 (zip 45620 KB) [file 10514_2015_9534_MOESM1_ESM.zip › SupplementaryMaterial/UAV/maptiles/atile_3_4.png]

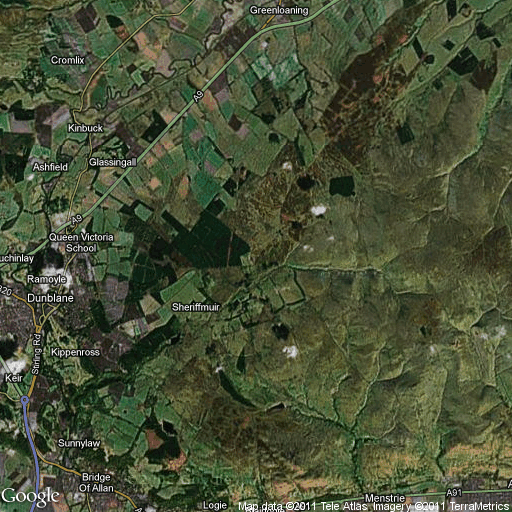

Supplement: Supplementary file 1 — Supplementary material 1 (zip 45620 KB) [file 10514_2015_9534_MOESM1_ESM.zip › SupplementaryMaterial/UAV/maptiles/atile_3_5.png]

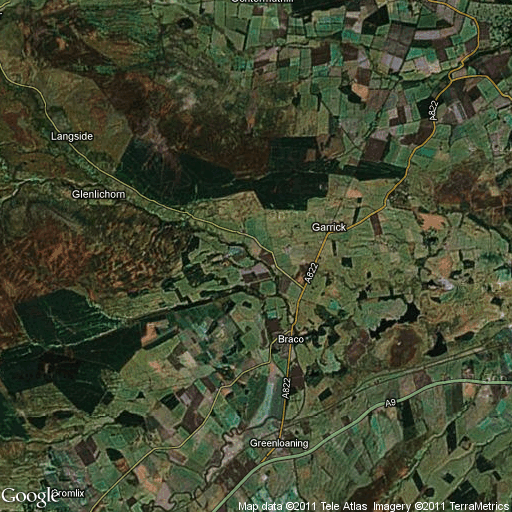

Supplement: Supplementary file 1 — Supplementary material 1 (zip 45620 KB) [file 10514_2015_9534_MOESM1_ESM.zip › SupplementaryMaterial/UAV/maptiles/atile_3_6.png]

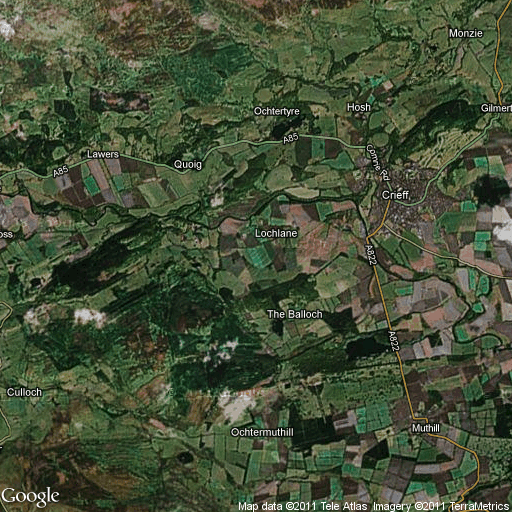

Supplement: Supplementary file 1 — Supplementary material 1 (zip 45620 KB) [file 10514_2015_9534_MOESM1_ESM.zip › SupplementaryMaterial/UAV/maptiles/atile_3_7.png]

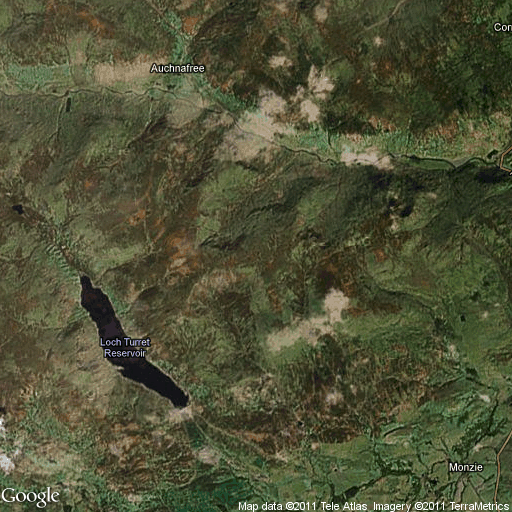

Supplement: Supplementary file 1 — Supplementary material 1 (zip 45620 KB) [file 10514_2015_9534_MOESM1_ESM.zip › SupplementaryMaterial/UAV/maptiles/atile_3_8.png]

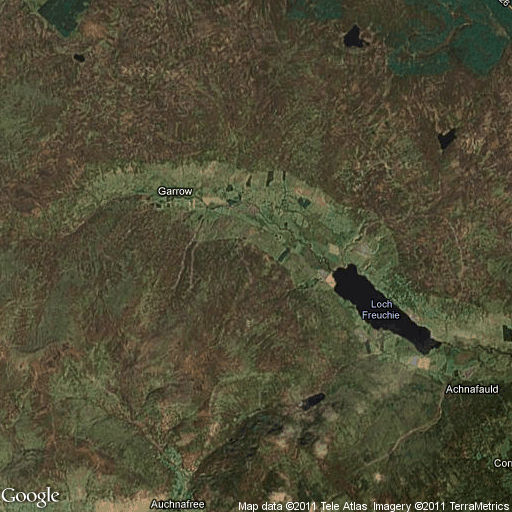

Supplement: Supplementary file 1 — Supplementary material 1 (zip 45620 KB) [file 10514_2015_9534_MOESM1_ESM.zip › SupplementaryMaterial/UAV/maptiles/atile_3_9.png]

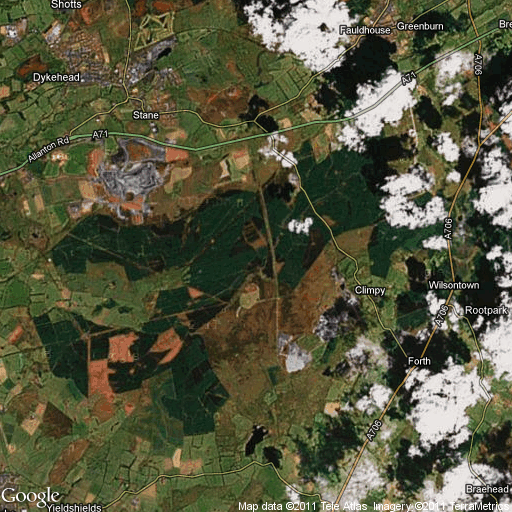

Supplement: Supplementary file 1 — Supplementary material 1 (zip 45620 KB) [file 10514_2015_9534_MOESM1_ESM.zip › SupplementaryMaterial/UAV/maptiles/atile_4_0.png]

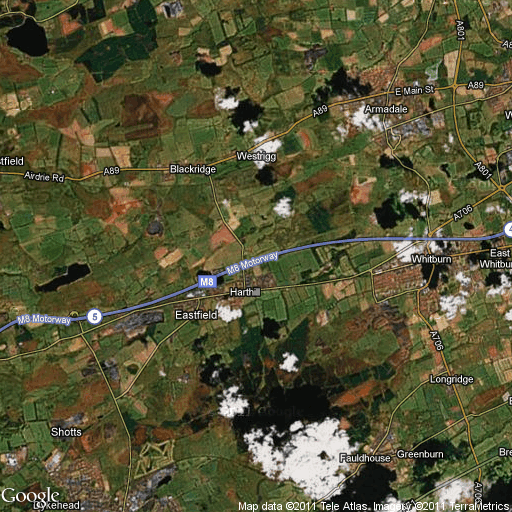

Supplement: Supplementary file 1 — Supplementary material 1 (zip 45620 KB) [file 10514_2015_9534_MOESM1_ESM.zip › SupplementaryMaterial/UAV/maptiles/atile_4_1.png]

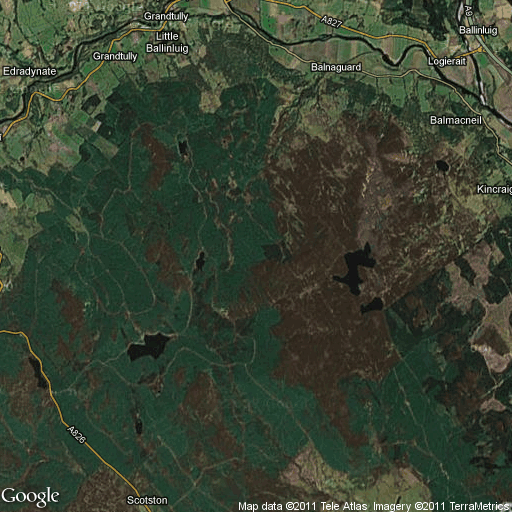

Supplement: Supplementary file 1 — Supplementary material 1 (zip 45620 KB) [file 10514_2015_9534_MOESM1_ESM.zip › SupplementaryMaterial/UAV/maptiles/atile_4_10.png]

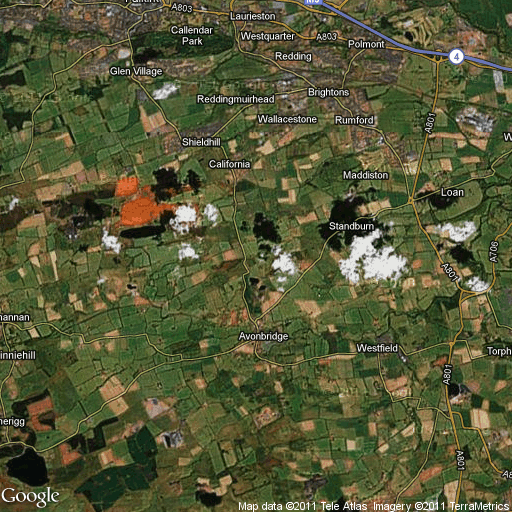

Supplement: Supplementary file 1 — Supplementary material 1 (zip 45620 KB) [file 10514_2015_9534_MOESM1_ESM.zip › SupplementaryMaterial/UAV/maptiles/atile_4_2.png]

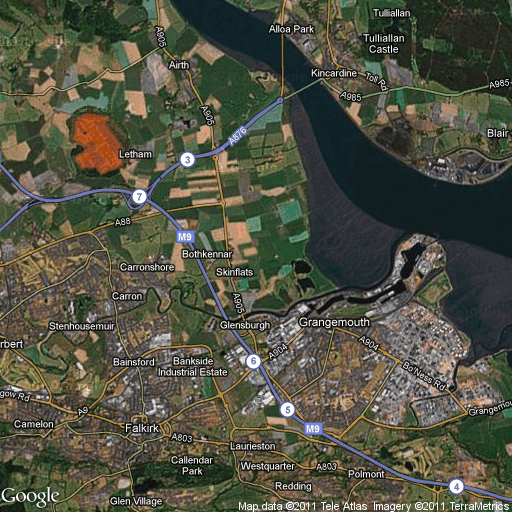

Supplement: Supplementary file 1 — Supplementary material 1 (zip 45620 KB) [file 10514_2015_9534_MOESM1_ESM.zip › SupplementaryMaterial/UAV/maptiles/atile_4_3.png]

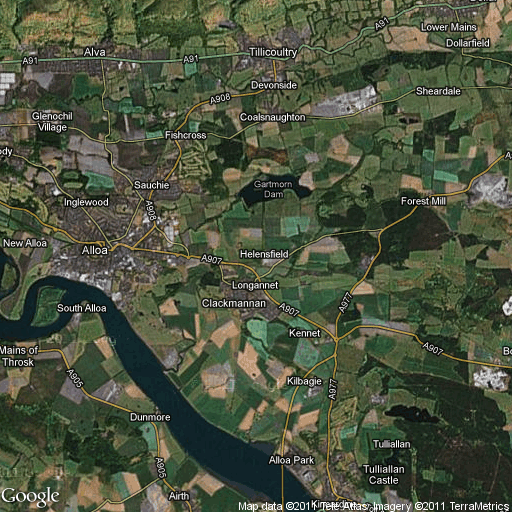

Supplement: Supplementary file 1 — Supplementary material 1 (zip 45620 KB) [file 10514_2015_9534_MOESM1_ESM.zip › SupplementaryMaterial/UAV/maptiles/atile_4_4.png]

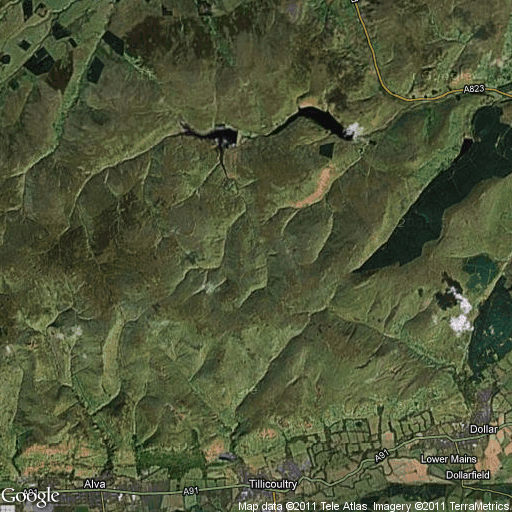

Supplement: Supplementary file 1 — Supplementary material 1 (zip 45620 KB) [file 10514_2015_9534_MOESM1_ESM.zip › SupplementaryMaterial/UAV/maptiles/atile_4_5.png]

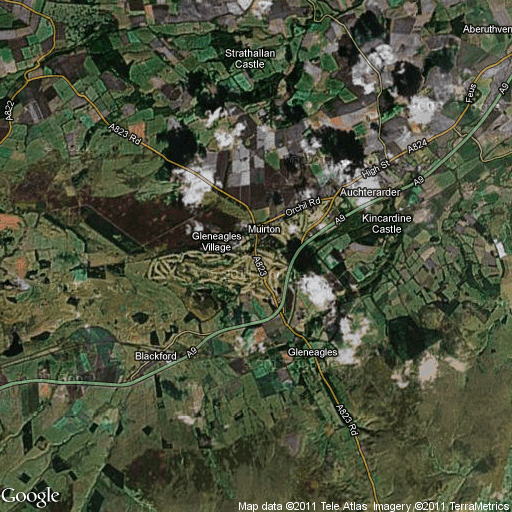

Supplement: Supplementary file 1 — Supplementary material 1 (zip 45620 KB) [file 10514_2015_9534_MOESM1_ESM.zip › SupplementaryMaterial/UAV/maptiles/atile_4_6.png]

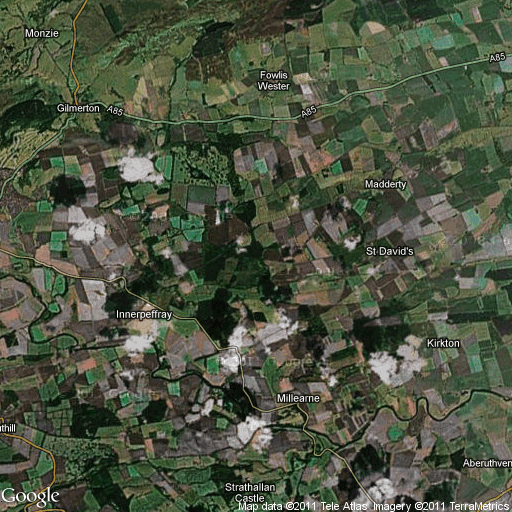

Supplement: Supplementary file 1 — Supplementary material 1 (zip 45620 KB) [file 10514_2015_9534_MOESM1_ESM.zip › SupplementaryMaterial/UAV/maptiles/atile_4_7.png]

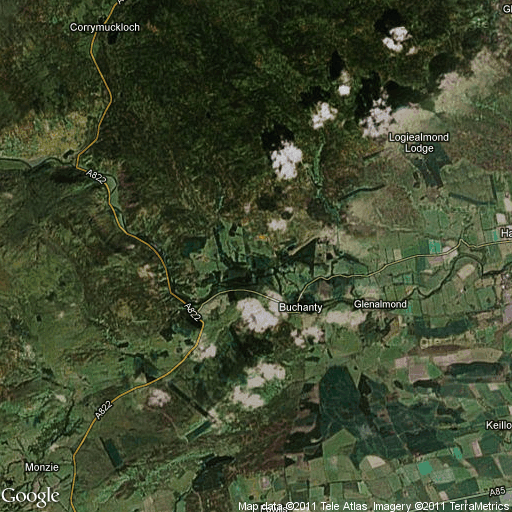

Supplement: Supplementary file 1 — Supplementary material 1 (zip 45620 KB) [file 10514_2015_9534_MOESM1_ESM.zip › SupplementaryMaterial/UAV/maptiles/atile_4_8.png]

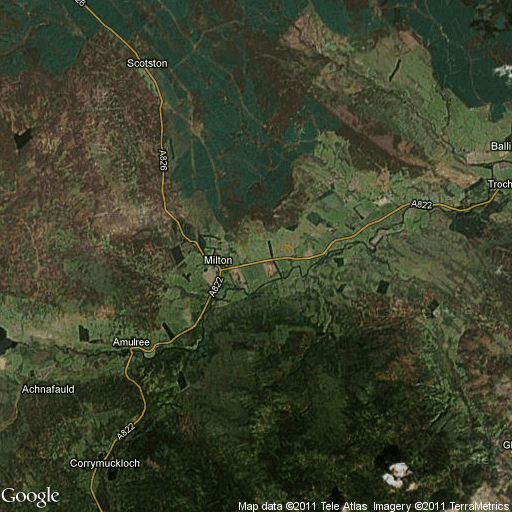

Supplement: Supplementary file 1 — Supplementary material 1 (zip 45620 KB) [file 10514_2015_9534_MOESM1_ESM.zip › SupplementaryMaterial/UAV/maptiles/atile_4_9.png]

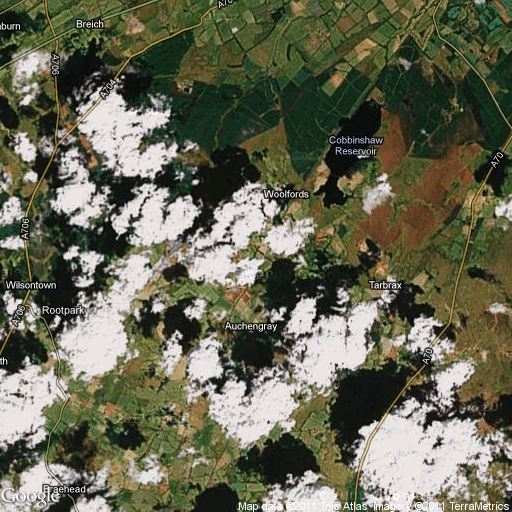

Supplement: Supplementary file 1 — Supplementary material 1 (zip 45620 KB) [file 10514_2015_9534_MOESM1_ESM.zip › SupplementaryMaterial/UAV/maptiles/atile_5_0.png]

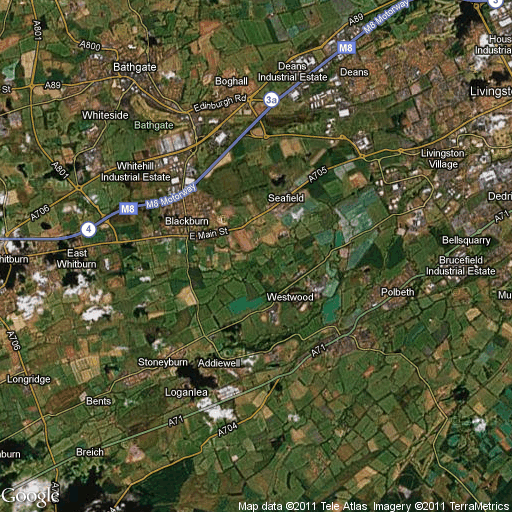

Supplement: Supplementary file 1 — Supplementary material 1 (zip 45620 KB) [file 10514_2015_9534_MOESM1_ESM.zip › SupplementaryMaterial/UAV/maptiles/atile_5_1.png]

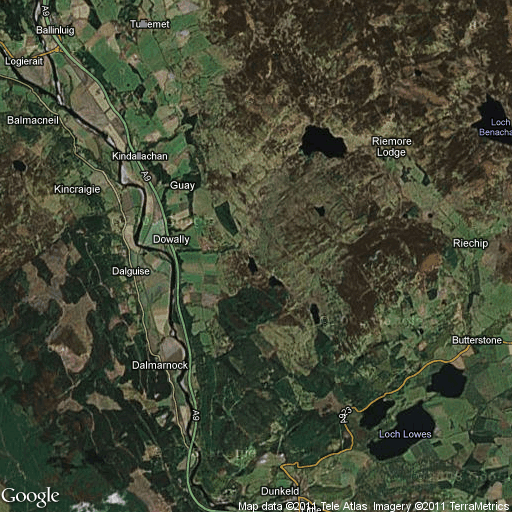

Supplement: Supplementary file 1 — Supplementary material 1 (zip 45620 KB) [file 10514_2015_9534_MOESM1_ESM.zip › SupplementaryMaterial/UAV/maptiles/atile_5_10.png]

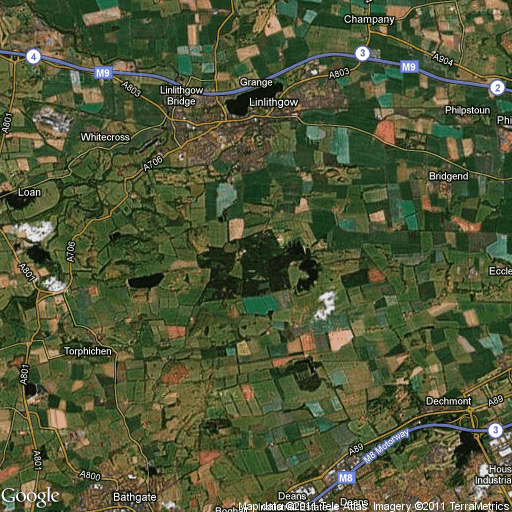

Supplement: Supplementary file 1 — Supplementary material 1 (zip 45620 KB) [file 10514_2015_9534_MOESM1_ESM.zip › SupplementaryMaterial/UAV/maptiles/atile_5_2.png]

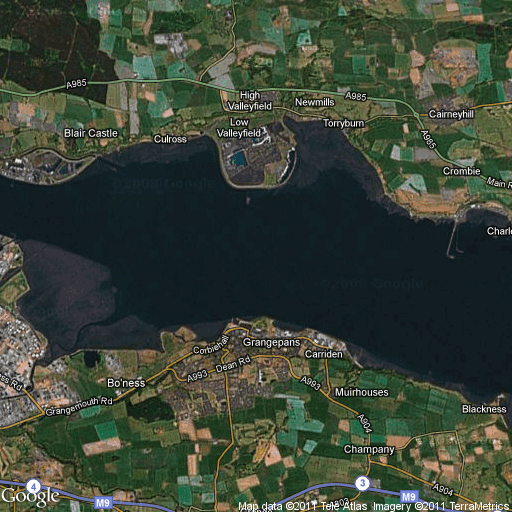

Supplement: Supplementary file 1 — Supplementary material 1 (zip 45620 KB) [file 10514_2015_9534_MOESM1_ESM.zip › SupplementaryMaterial/UAV/maptiles/atile_5_3.png]

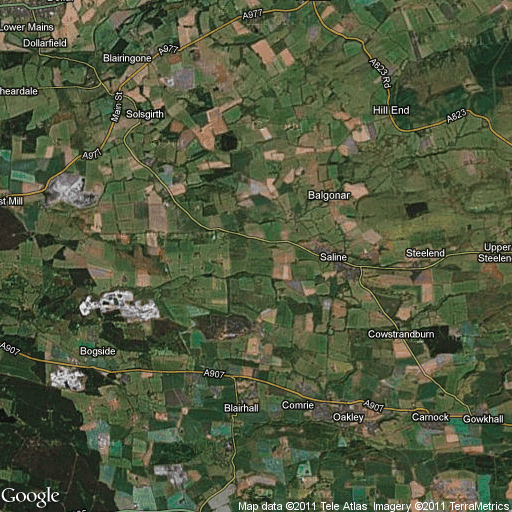

Supplement: Supplementary file 1 — Supplementary material 1 (zip 45620 KB) [file 10514_2015_9534_MOESM1_ESM.zip › SupplementaryMaterial/UAV/maptiles/atile_5_4.png]

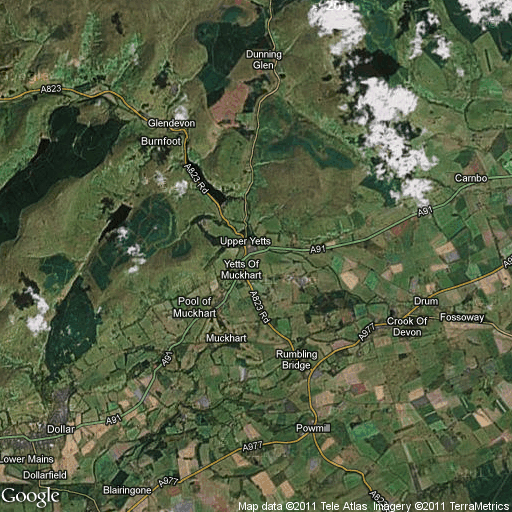

Supplement: Supplementary file 1 — Supplementary material 1 (zip 45620 KB) [file 10514_2015_9534_MOESM1_ESM.zip › SupplementaryMaterial/UAV/maptiles/atile_5_5.png]

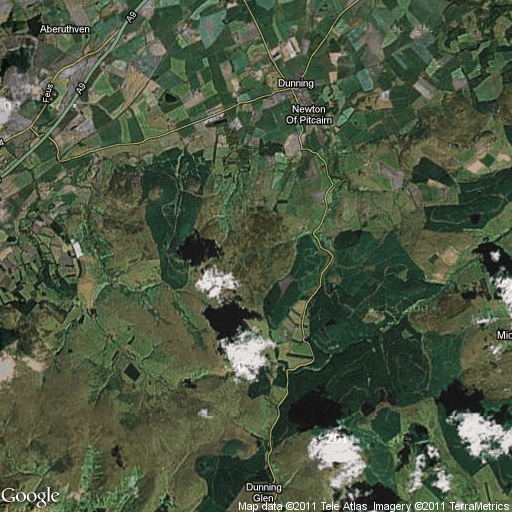

Supplement: Supplementary file 1 — Supplementary material 1 (zip 45620 KB) [file 10514_2015_9534_MOESM1_ESM.zip › SupplementaryMaterial/UAV/maptiles/atile_5_6.png]

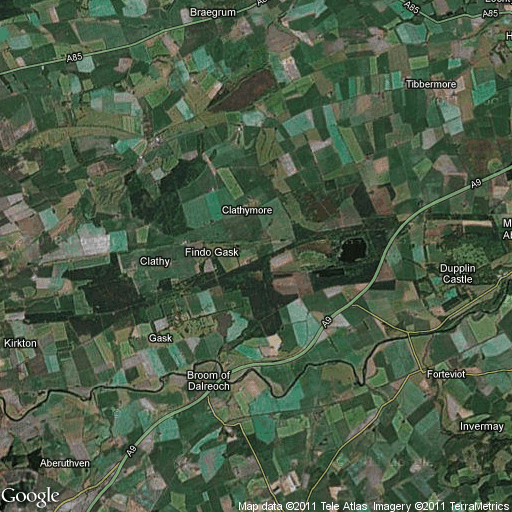

Supplement: Supplementary file 1 — Supplementary material 1 (zip 45620 KB) [file 10514_2015_9534_MOESM1_ESM.zip › SupplementaryMaterial/UAV/maptiles/atile_5_7.png]

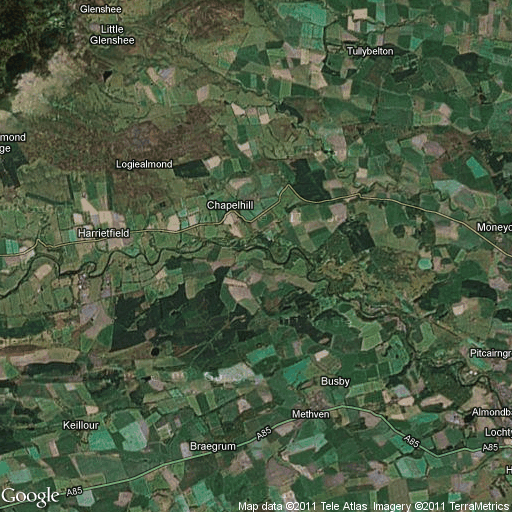

Supplement: Supplementary file 1 — Supplementary material 1 (zip 45620 KB) [file 10514_2015_9534_MOESM1_ESM.zip › SupplementaryMaterial/UAV/maptiles/atile_5_8.png]

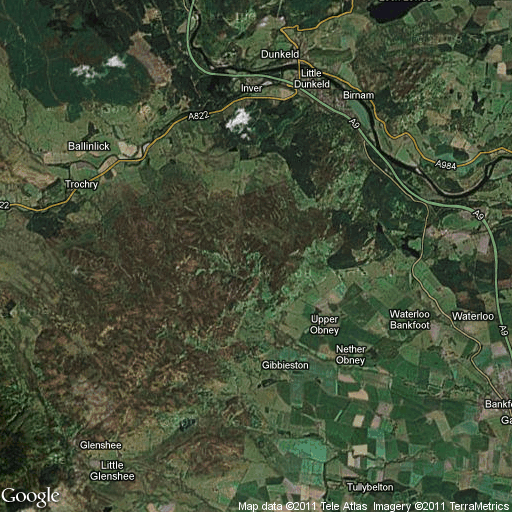

Supplement: Supplementary file 1 — Supplementary material 1 (zip 45620 KB) [file 10514_2015_9534_MOESM1_ESM.zip › SupplementaryMaterial/UAV/maptiles/atile_5_9.png]

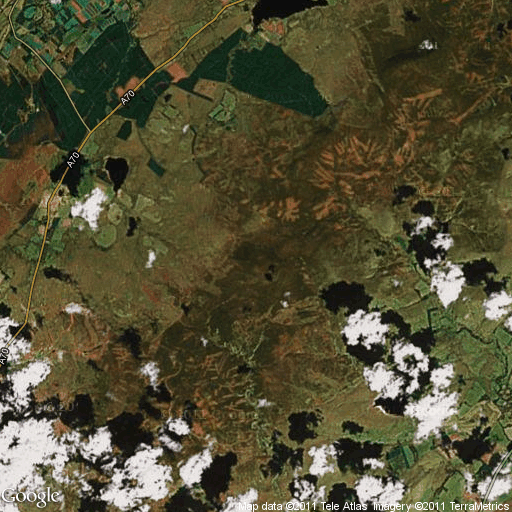

Supplement: Supplementary file 1 — Supplementary material 1 (zip 45620 KB) [file 10514_2015_9534_MOESM1_ESM.zip › SupplementaryMaterial/UAV/maptiles/atile_6_0.png]

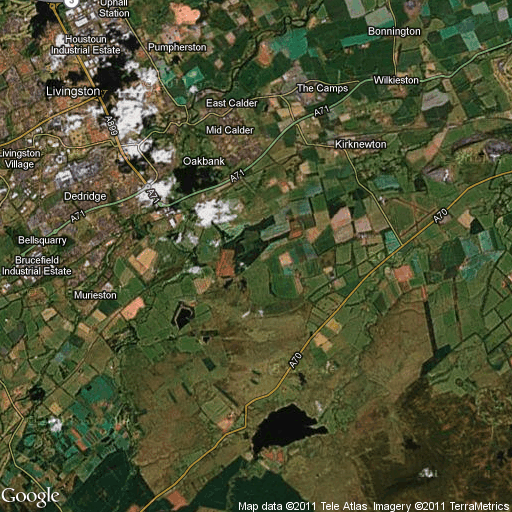

Supplement: Supplementary file 1 — Supplementary material 1 (zip 45620 KB) [file 10514_2015_9534_MOESM1_ESM.zip › SupplementaryMaterial/UAV/maptiles/atile_6_1.png]

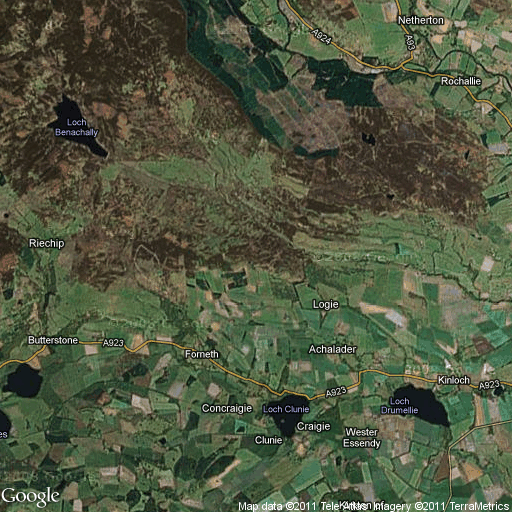

Supplement: Supplementary file 1 — Supplementary material 1 (zip 45620 KB) [file 10514_2015_9534_MOESM1_ESM.zip › SupplementaryMaterial/UAV/maptiles/atile_6_10.png]

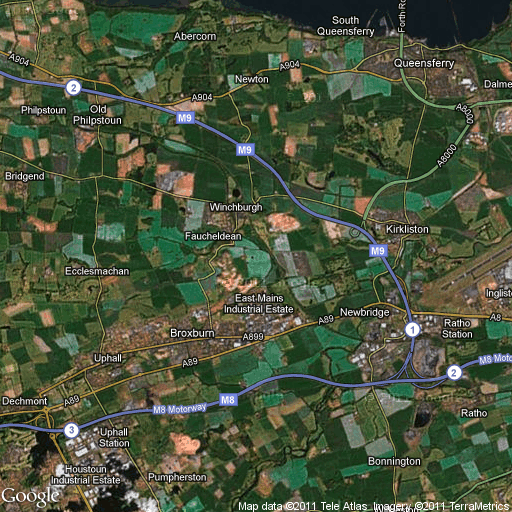

Supplement: Supplementary file 1 — Supplementary material 1 (zip 45620 KB) [file 10514_2015_9534_MOESM1_ESM.zip › SupplementaryMaterial/UAV/maptiles/atile_6_2.png]

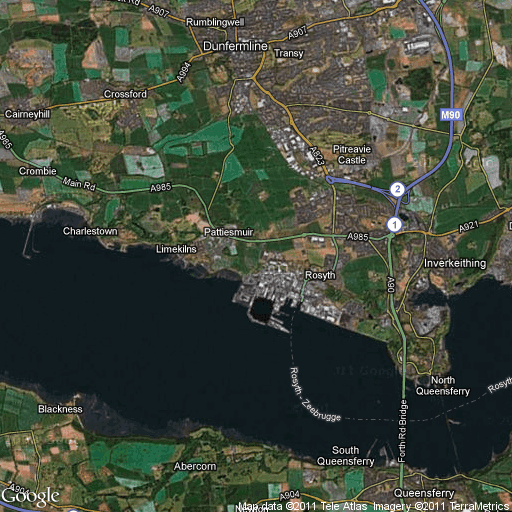

Supplement: Supplementary file 1 — Supplementary material 1 (zip 45620 KB) [file 10514_2015_9534_MOESM1_ESM.zip › SupplementaryMaterial/UAV/maptiles/atile_6_3.png]

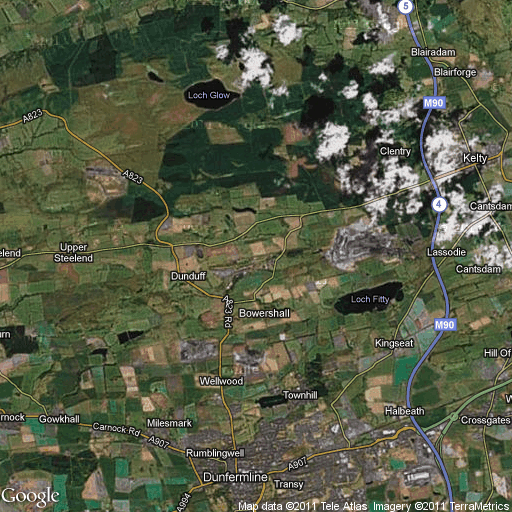

Supplement: Supplementary file 1 — Supplementary material 1 (zip 45620 KB) [file 10514_2015_9534_MOESM1_ESM.zip › SupplementaryMaterial/UAV/maptiles/atile_6_4.png]

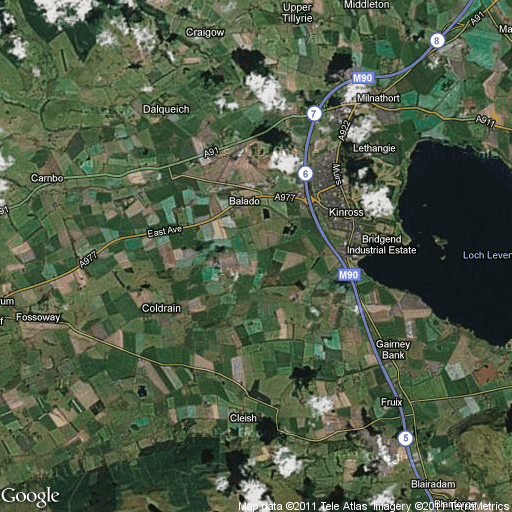

Supplement: Supplementary file 1 — Supplementary material 1 (zip 45620 KB) [file 10514_2015_9534_MOESM1_ESM.zip › SupplementaryMaterial/UAV/maptiles/atile_6_5.png]

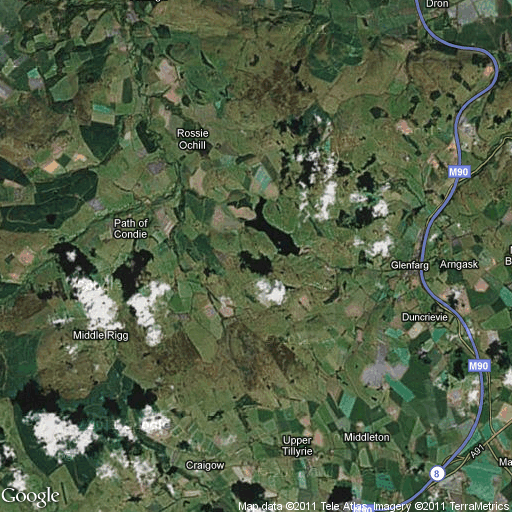

Supplement: Supplementary file 1 — Supplementary material 1 (zip 45620 KB) [file 10514_2015_9534_MOESM1_ESM.zip › SupplementaryMaterial/UAV/maptiles/atile_6_6.png]

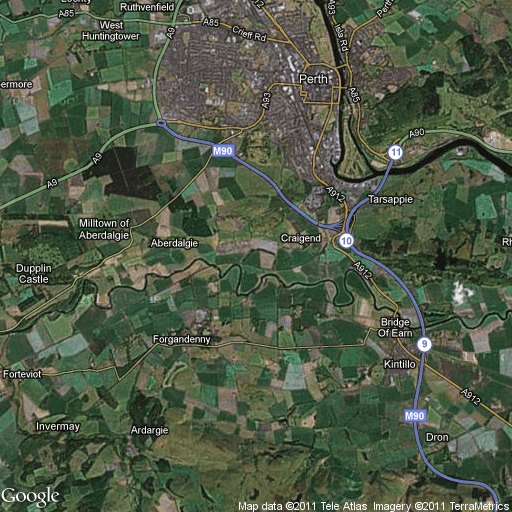

Supplement: Supplementary file 1 — Supplementary material 1 (zip 45620 KB) [file 10514_2015_9534_MOESM1_ESM.zip › SupplementaryMaterial/UAV/maptiles/atile_6_7.png]

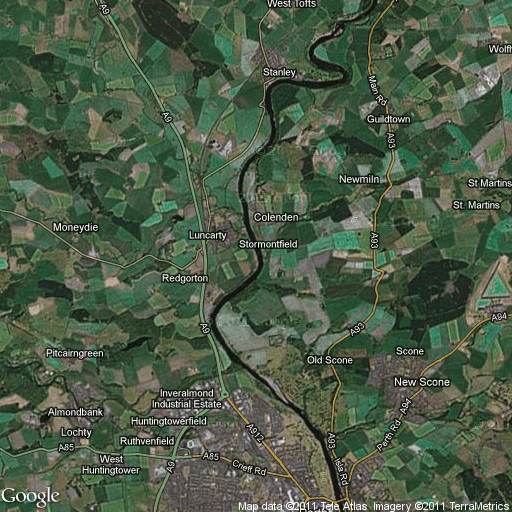

Supplement: Supplementary file 1 — Supplementary material 1 (zip 45620 KB) [file 10514_2015_9534_MOESM1_ESM.zip › SupplementaryMaterial/UAV/maptiles/atile_6_8.png]

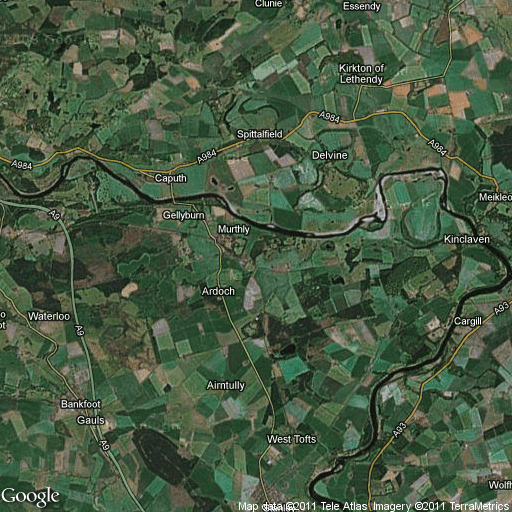

Supplement: Supplementary file 1 — Supplementary material 1 (zip 45620 KB) [file 10514_2015_9534_MOESM1_ESM.zip › SupplementaryMaterial/UAV/maptiles/atile_6_9.png]

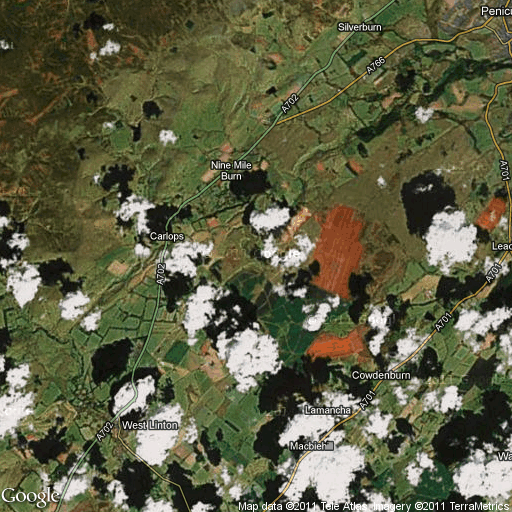

Supplement: Supplementary file 1 — Supplementary material 1 (zip 45620 KB) [file 10514_2015_9534_MOESM1_ESM.zip › SupplementaryMaterial/UAV/maptiles/atile_7_0.png]

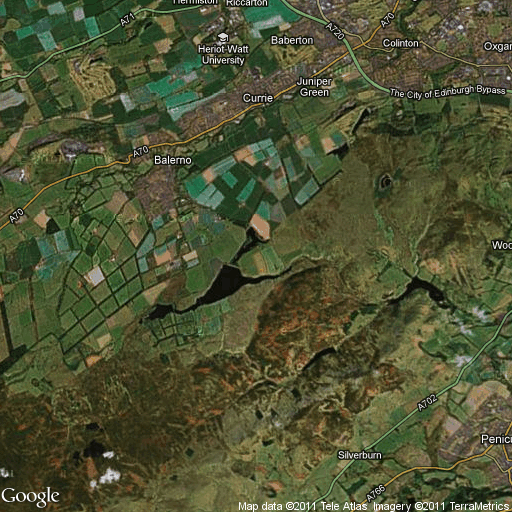

Supplement: Supplementary file 1 — Supplementary material 1 (zip 45620 KB) [file 10514_2015_9534_MOESM1_ESM.zip › SupplementaryMaterial/UAV/maptiles/atile_7_1.png]

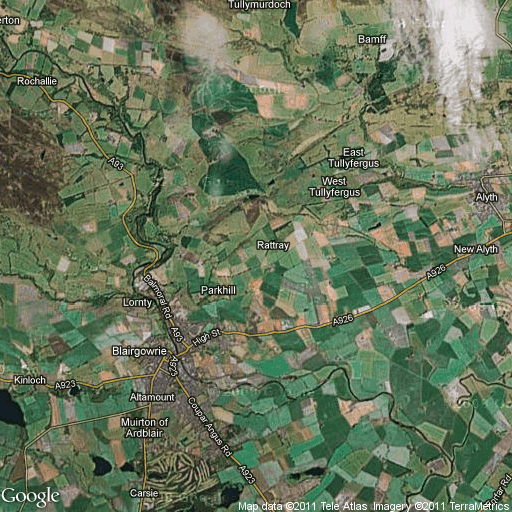

Supplement: Supplementary file 1 — Supplementary material 1 (zip 45620 KB) [file 10514_2015_9534_MOESM1_ESM.zip › SupplementaryMaterial/UAV/maptiles/atile_7_10.png]

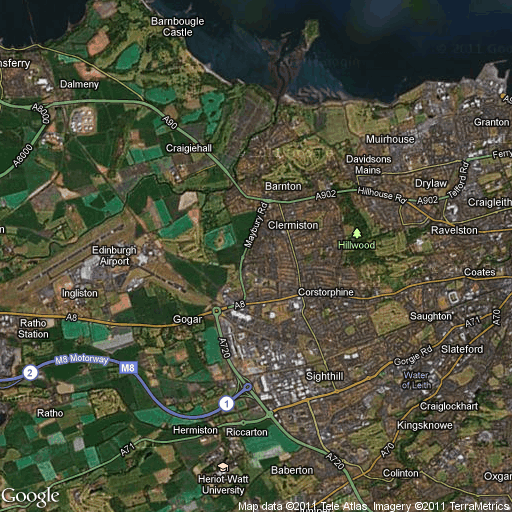

Supplement: Supplementary file 1 — Supplementary material 1 (zip 45620 KB) [file 10514_2015_9534_MOESM1_ESM.zip › SupplementaryMaterial/UAV/maptiles/atile_7_2.png]

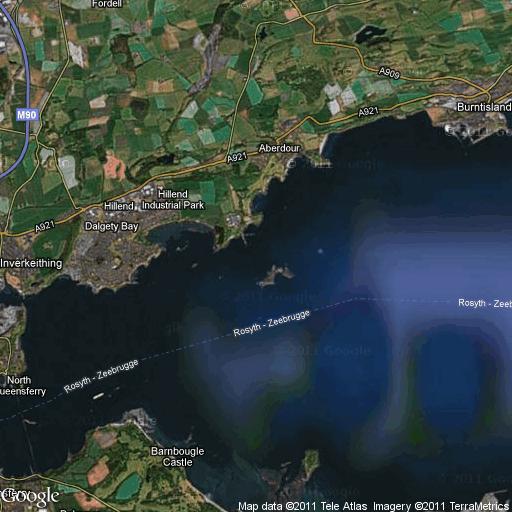

Supplement: Supplementary file 1 — Supplementary material 1 (zip 45620 KB) [file 10514_2015_9534_MOESM1_ESM.zip › SupplementaryMaterial/UAV/maptiles/atile_7_3.png]

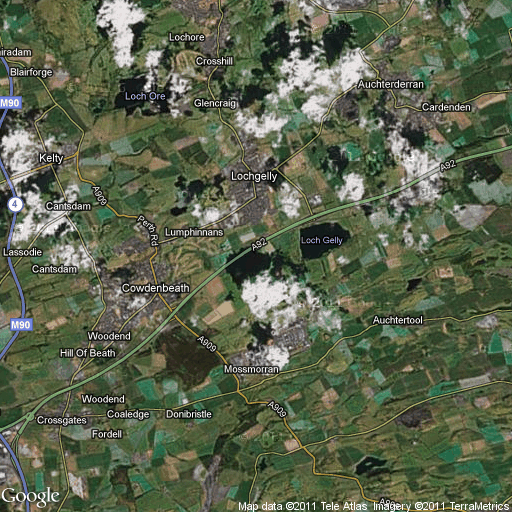

Supplement: Supplementary file 1 — Supplementary material 1 (zip 45620 KB) [file 10514_2015_9534_MOESM1_ESM.zip › SupplementaryMaterial/UAV/maptiles/atile_7_4.png]

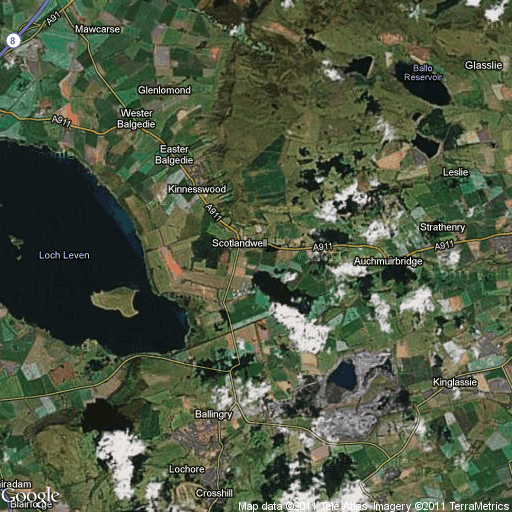

Supplement: Supplementary file 1 — Supplementary material 1 (zip 45620 KB) [file 10514_2015_9534_MOESM1_ESM.zip › SupplementaryMaterial/UAV/maptiles/atile_7_5.png]

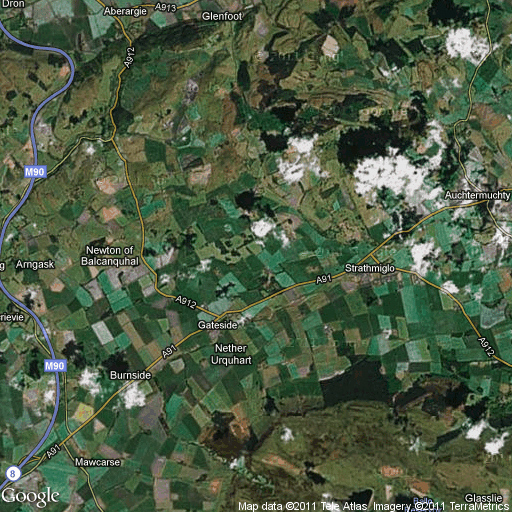

Supplement: Supplementary file 1 — Supplementary material 1 (zip 45620 KB) [file 10514_2015_9534_MOESM1_ESM.zip › SupplementaryMaterial/UAV/maptiles/atile_7_6.png]

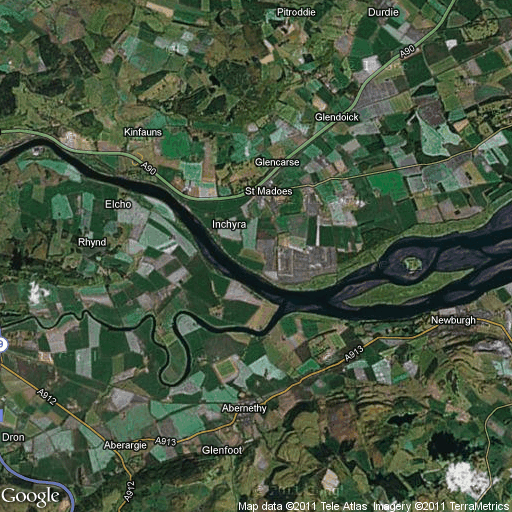

Supplement: Supplementary file 1 — Supplementary material 1 (zip 45620 KB) [file 10514_2015_9534_MOESM1_ESM.zip › SupplementaryMaterial/UAV/maptiles/atile_7_7.png]

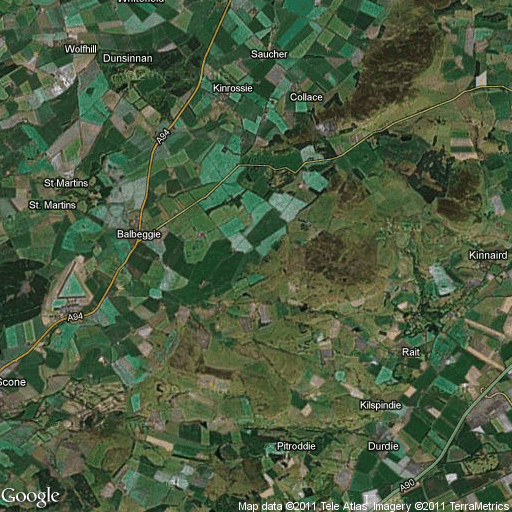

Supplement: Supplementary file 1 — Supplementary material 1 (zip 45620 KB) [file 10514_2015_9534_MOESM1_ESM.zip › SupplementaryMaterial/UAV/maptiles/atile_7_8.png]

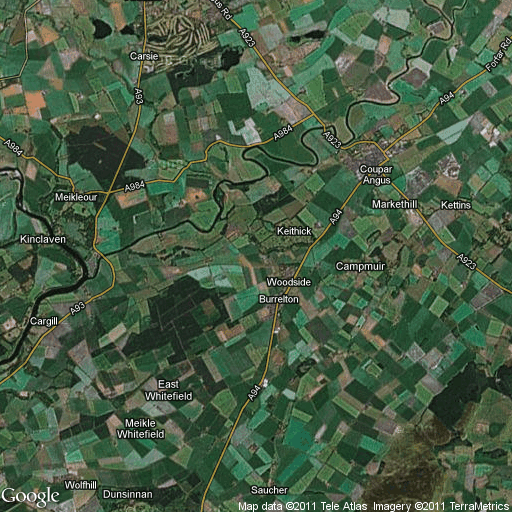

Supplement: Supplementary file 1 — Supplementary material 1 (zip 45620 KB) [file 10514_2015_9534_MOESM1_ESM.zip › SupplementaryMaterial/UAV/maptiles/atile_7_9.png]

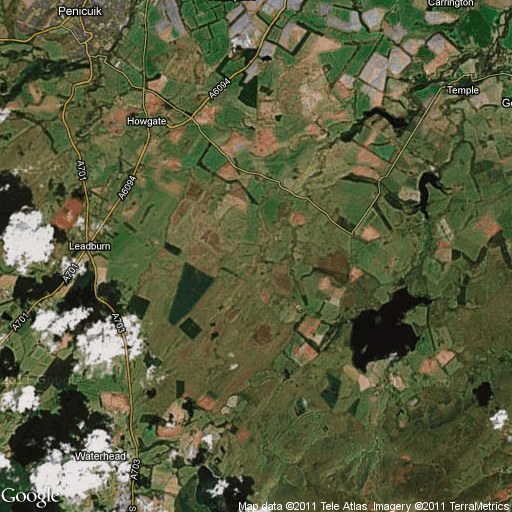

Supplement: Supplementary file 1 — Supplementary material 1 (zip 45620 KB) [file 10514_2015_9534_MOESM1_ESM.zip › SupplementaryMaterial/UAV/maptiles/atile_8_0.png]
